# Design, synthesis, molecular modelling and *in vitro* screening of monoamine oxidase inhibitory activities of novel quinazolyl hydrazine derivatives

**DOI:** 10.1098/rsos.200050

**Published:** 2020-04-22

**Authors:** Adel Amer, Abdelrahman H. Hegazi, Mohammed Khalil Alshekh, Hany E. A. Ahmed, Saied M. Soliman, Antonin Maniquet, Rona R. Ramsay

**Affiliations:** 1 Department of Chemistry, College of Science, Taibah University, Al-Madinah Al-Munawarah, Saudi Arabia; 2 Department of Chemistry, Faculty of Science, Alexandria University, Alexandria 21321, Egypt; 3 Pharmacognosy and Pharmaceutical Chemistry Department, Pharmacy College, Taibah University, Al-Madinha Al-Munawarah, Saudi Arabia; 4 Pharmaceutical Organic Chemistry Department, Faculty of Pharmacy, Al-Azhar University, Cairo, Egypt; 5 Biomedical Sciences Research Complex, University of St Andrews, Biomolecular Sciences Building, North Haugh, St Andrews KY16 9ST, UK

**Keywords:** monoamine oxidase inhibitors, antidepressant, quinazoline analogues, structure–activity relationships

## Abstract

A new series of N'-substituted benzylidene-2-(4-oxo-2-phenyl-1,4-dihydroquinazolin-3(2H)-yl)acetohydrazide (**5a–5h**) has been synthesized, characterized by FT-IR, NMR spectroscopy and mass spectrometry and tested against human monoamine oxidase (MAO) A and B. Only (4-hydroxy-3-methoxybenzylidene) substituted compounds gave submicromolar inhibition of MAO-A and MAO-B. Changing the phenyl substituent to methyl on the unsaturated quinazoline ring (**12a–12d**) decreased inhibition, but a less flexible linker (**14a–14d**) resulted in selective micromolar inhibition of hMAO-B providing insight for ongoing design.

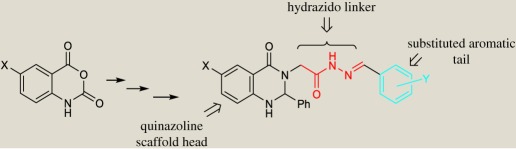

## 1. Introduction

Depression has been reported to be the fourth global burden of disease, with nearly 12% of the global disability-adjusted life years [1]. In the Kingdom of Saudi Arabia, improvements in socioeconomic status have been shown to be associated with increased chronic diseases including chronic mental diseases like depression [2–7]. Monoamine oxidases (MAO), enzymes containing covalently bound flavin adenine dinucleotide (FAD) as the cofactor, are located on the mitochondrial outer membrane where they oxidize various physiologically and pathologically important monoamines. These neurotransmitters and hormones include serotonin, noradrenaline and dopamine that function to regulate movement, emotion, reward, cognition, memory and learning [8–10].

The two isoforms of MAO (MAO-A and MAO-B) are characterized by different affinities for inhibitors and different specificities for substrates [11,12]. MAO-A preferentially metabolizes serotonin, adrenaline, and noradrenaline [13], whereas 2-phenylethylamine and benzylamine are predominantly metabolized by MAO-B [14]. Tyramine and dopamine are common substrates for both isoenzymes [15]. The therapeutic interest in monoamine inhibitors (MAOIs) covers two major categories: MAO-A inhibitors are used mostly in the treatment of mental disorders, in particular depression and anxiety [16–18], whereas MAO-B inhibitors are used in the treatment of Parkinson's disease and are of interest against Alzheimer's disease [19,20].

**Chart 1.**
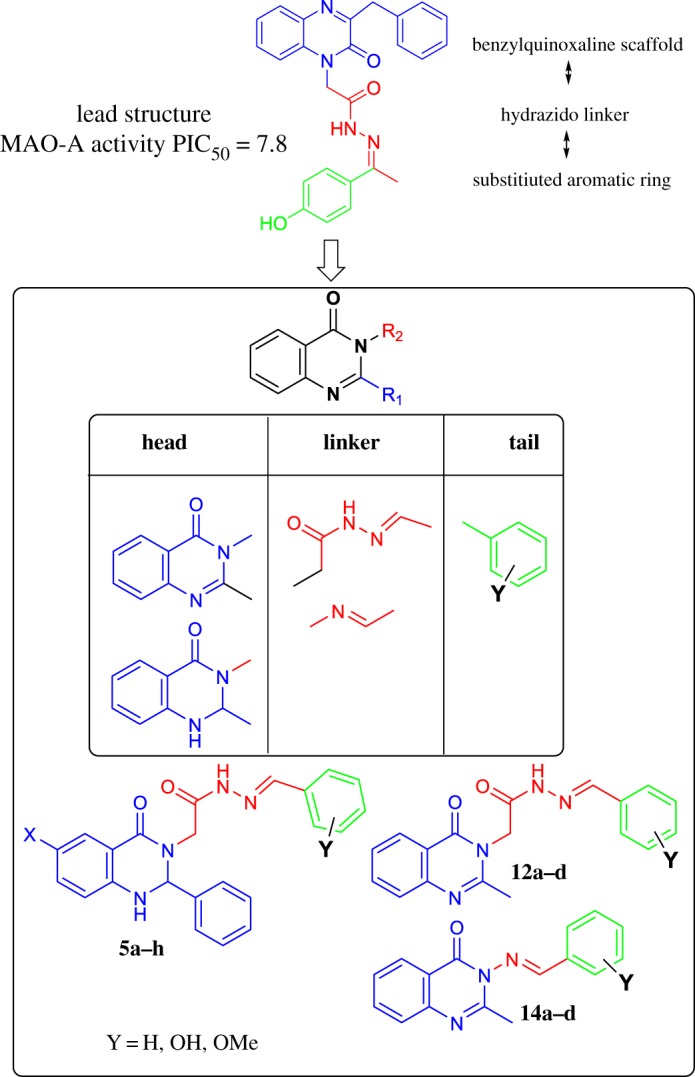
Planned modification and newly designed quinazolyl hydrazine derivative monoamine inhibitors.

A major goal of our group is to develop compounds capable of inhibiting monoamine oxidase A as a target for the treatment of depression and psychological disorders. We have designed and synthesized several series of compounds carrying 3-benzylquinoxaline and quinazoline scaffolds with selective activity towards MAO-A [21–25]. Based on the results, we decided to change the scaffold from benzyl quinoxaline to methyl and phenyl quinazolines as the prominent motif for discovery of variety of biologically active compounds. To further explore the structure–activity relationships of the new quinazoline class of MAO-A inhibitors, we have now synthesized and evaluated a series of N'-substituted benzylidene-2-(6-chloro-4-oxo-2-aryl-1,4-dihydroquinazolin-3(2H)-yl)acetohydrazides and N'-substituted benzylidene-2-(2-methyl-4-oxoquinazolin-3(4H)-yl)acetohydrazides for their ability to inhibit the enzymatic activity of both MAO isoforms. The rationale of work design was built upon different factors that include: (i) the presence of a hydrophobic nitrogen heterocyclic head (Quinazolyl moiety), (ii) the presence of the hydrazido functionality (–CO–NH–N=) connected to the N atom via a saturated carbon linker, and (iii) the presence of an electron-rich aromatic tail (Y = OH, OMe in type **5** compounds) (chart 1). We also prepared and examined a series of (*E*)-3-(substituted benzylideneamino)-2-methylquinazolin-4(3H)-ones which contain imino functionality (–HC=N–) linker (compounds **14**).

**Scheme 1.**
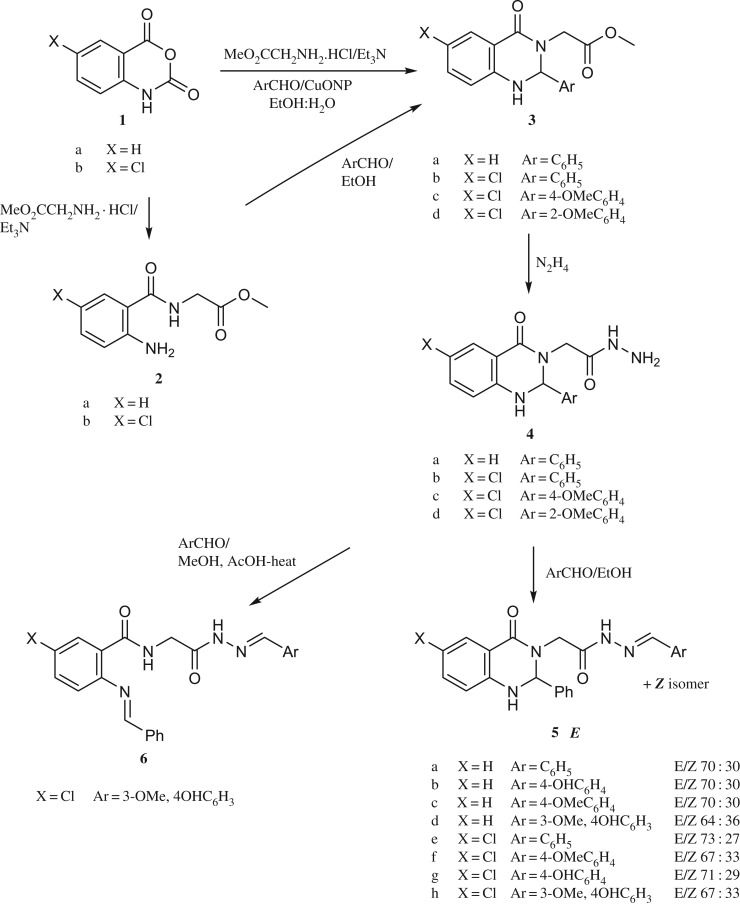
Synthesis of compounds **3**, **4**, **5** and **6**.

## 2. Results and discussion

### 2.1. Organic synthesis

**Figure 1.**
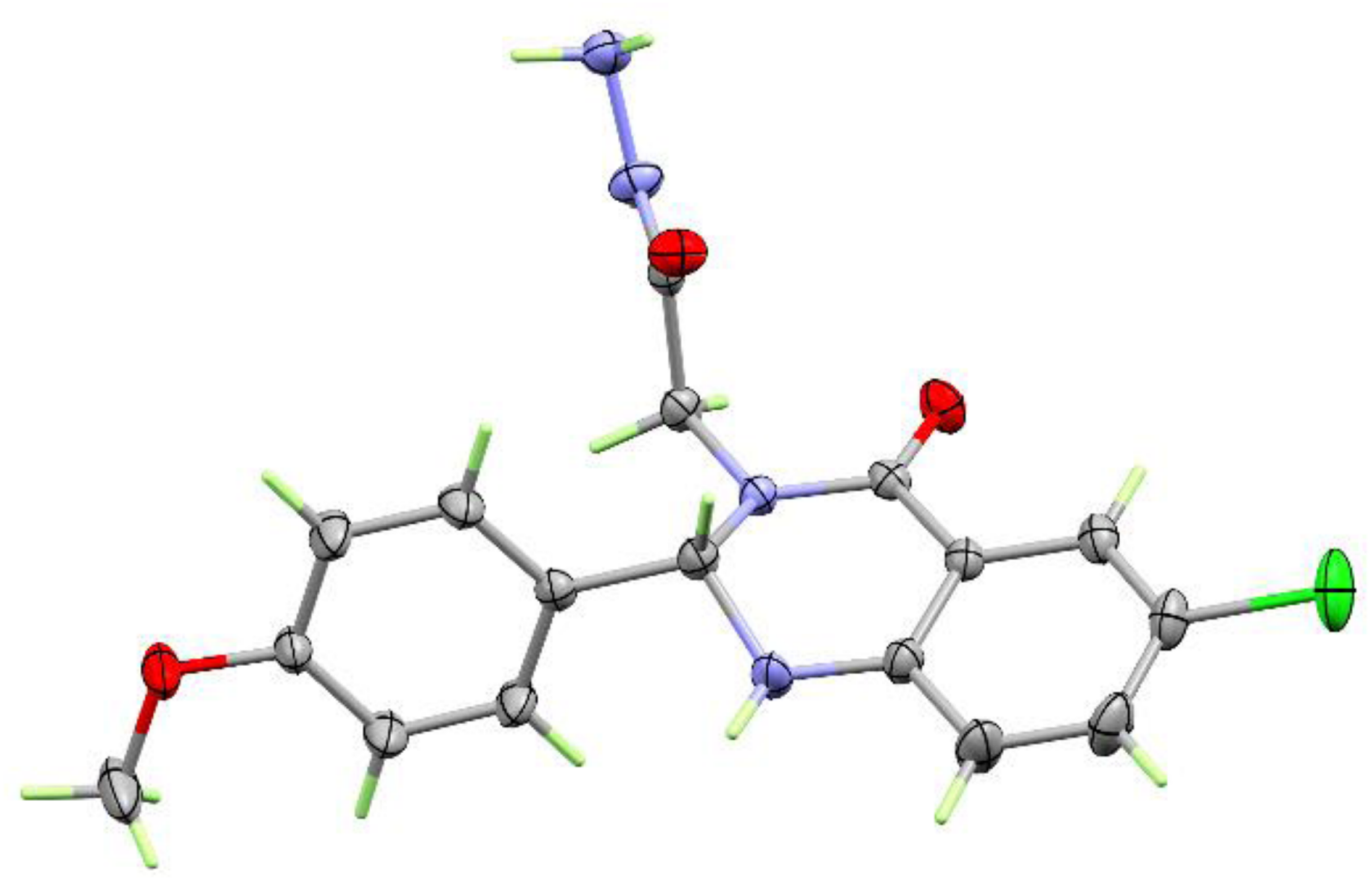
Perspective drawing of compound **4c**.

The synthetic route for the target compounds **5a–5h** is depicted in scheme 1. Allowing isatoic anhydride **1a–1b** to react with methyl glycine hydrochloride and triethylamine afforded the intermediate methyl (2-aminobenzoyl)glycinate **2a–2b**. The cyclocondensation of compound **2a–2b** and aromatic aldehyde in ethanol under reflux processed to furnish **3a–3d**. One-pot three components synthetic route to type **3** was achieved using metal oxides nanoparticles [26]. Allowing isatoic anhydride to react with methyl glycine hydrochloride, triethylamine and aromatic aldehyde in ethanol–water mixture in the presence of catalytic amount of copper oxide nanoparticles led to the formation of **3a–3d** in good yields. The treatment of the ester **3a–3d** with hydrazine hydrate afforded the corresponding hydrazides **4a–4d**. Their structures were confirmed by spectroscopic tools and secured by X-ray single crystal analysis of compound **4c** as a prototype (figure 1; electronic supplementary material, table S1).

**Figure 2.**
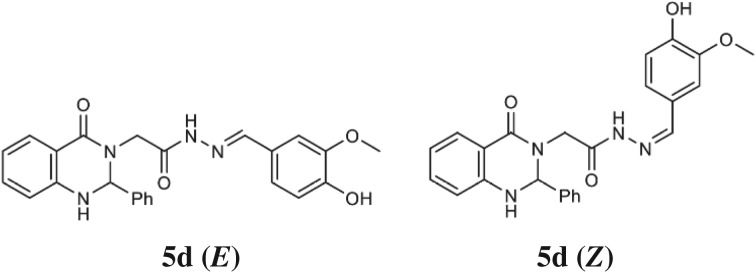
Structures of the *E/Z* isomers of **5d**.

Reaction of compounds **4a–4d** with different aromatic aldehydes in ethanolic solution and keeping the reaction mixture warm and stirring overnight furnished the desired products N'-benzylidene-2-(4-oxo-2-phenyl-1,4-dihydroquinazolin-3(2H)yl)acetohydrazide derivatives **5a–5h**. The NMR spectra of type **5** compounds reveal that they exist in two isomeric forms *E* and *Z* in dimethyl sulfoxide solution [27,28]. As a prototype, ^1^H NMR spectrum of **5d** (figure 2) showed two sets of two doublets in 64 : 36 ratio for the α-CH_2_ with coupling constant of 17.6 and 16.4 Hz at *δ* 3.64, 4.03 and 3.34, 4.53, respectively. The hydrazono N=CH appeared as singlets at *δ* 7.82 and 8.03. In ^13^C NMR, the α-C resonated at *δ* 45.42 and 46.32. These assignments were supported by the ^1^H–^1^H COSY NMR spectrum which confirmed the correlation assignments of α-CH_2_ and provided support to full peak assignments. The *sp*^3^CH resonated at *δ* 6.00 and correlated to two signals at *δ* 7.25 and 7.33 ppm of the NHs.

On the other hand, an attempt to accelerate the reaction **4b** and vanillin using few drops of acetic acid and heating the reaction mixture to reflux led to ring opening and the precipitation of **6**, as evident by NMR. The methylene protons showed one signal at *δ* 3.64 that correlates to carbon at *δ* 44.75 in heteronuclear single quantum coherence spectroscopy (HSQC) spectrum indicating the loss of the sp^3^CH chiral centre.

**Scheme 2.**
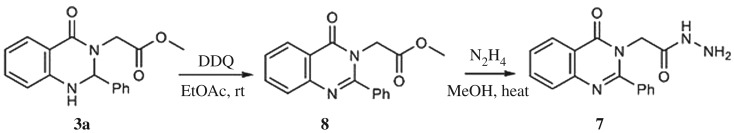
Synthetic route to compound **7**.

To synthesize the quinazolin-3(4H)-yl)acetohydrazide analogue **7** (scheme 2), the methyl 2-(4-oxo-2-phenyl-1,4-dihydroquinazolin-3(2H)-yl)acetate **3a** was oxidized with 2,3-dichloro-5,6-dicyano-1,4-benzoquinone (DDQ) to afford methyl 2-(4-oxo-2-phenylquinazolin-3(4H)-yl)acetate **8** [29]. Hydrazinolysis of **8** with hydrazine hydrate furnished **7** which was confirmed by spectral data and its melting point matches the literature [30].

**Scheme 3.**
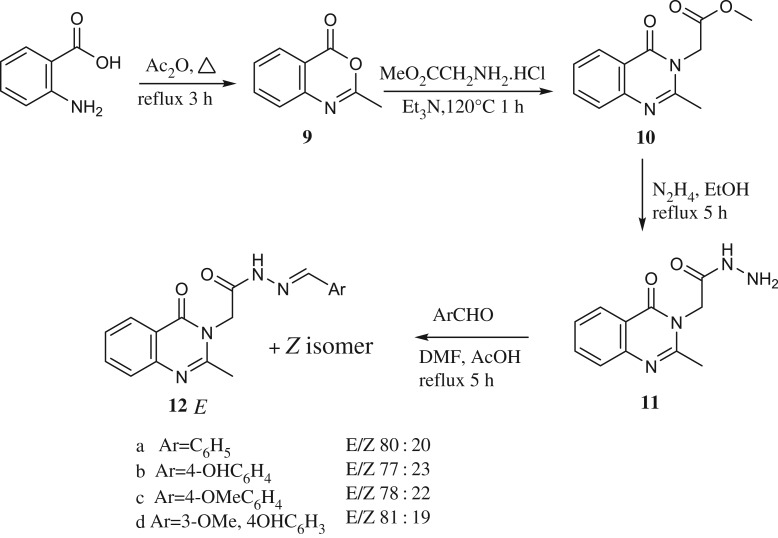
Synthesis of compounds **12**.

For the comparative study, compounds **12a–12d** were prepared using a method modified from the one reported earlier [31,32] (scheme 3) starting from 2-methyl-4H-benzo[d][1,3]oxazin-4-one **9** to **10** which upon hydrazinolysis furnished **11**. The reaction of compound **11** with aromatic aldehydes in dimethylformamide and few drops of acetic acid furnished N'-substituted benzylidene-2-(2-methyl-4-oxoquinazolin-3(4H)-yl)acetohydrazide **12a–12d** in good yield. According to the obtained NMR spectra, these compounds exist as a mixture of *E* and *Z* (see Experimental section) and not as reported earlier as a single isomers [31,32].

**Scheme 4.**
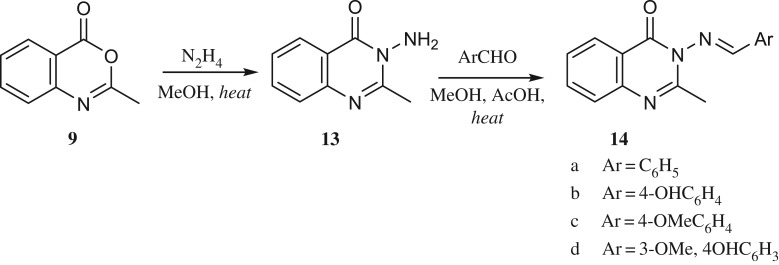
Synthetic route to compounds **14**.

The reaction sequence that led to the synthesis of (*E*)-3-(substituted benzylideneamino)-2-methylquinazolin-4(3H)-one **14a–14d** is shown in scheme 4. Thus, compound **9** was converted to **13** by reacting with hydrazine hydrate [33], which upon condensation with various aromatic aldehydes furnished **14a–14d** [34–38]. Their structures were found in agreement with the assigned molecular structure and confirmed by IR, NMR spectroscopy and mass spectrometry.

### 2.2. MAO inhibition

All compounds were screened for inhibition of the activity of membrane-bound human MAO-A and MAO-B at 10 µM in the presence of tyramine at 2 × *K*_M_ (0.8 mM for MAO-A and 0.32 mM for MAO-B) (table 1). Apart from **5d**, **5h**, **11** and **12b**, which gave equal inhibition of MAO-A and MAO-B in this screen, the compounds were slightly selective for MAO-B (see electronic supplementary material, figure S1). For the compounds that gave more than 30% inhibition, the IC_50_ was determined using seven concentrations of each compound (0.01–300 µM) with tyramine substrate at 2 × *K*_M_. The MAO-A and MAO-B IC_50_ values for these selected compounds are given in table 1 accompanied by the maximum extent of the inhibition observed. Incomplete inhibition of activity could arise either from indirect effects on these enzymes or from the compounds acting as substrates at the highest concentrations. Only compounds **5a–5h**, **12d** and **14a–14d** can be said to be classical inhibitors with **5a, 5h** and **12d** inhibiting both MAO-A and MAO-B. By contrast, **14a–14d** were highly MAO-B selective. The best inhibitors of MAO-A were **5d and 5h** with submicromolar IC_50_ values (0.27 ± 0.06 and 0.31 ± 0.06 µM, respectively). These compounds were slightly less effective against MAO-B activity (IC_50_ values 0.75 and 0.44 µM, respectively). Having both 3-OCH_3_ and 4-OH substituents on the tail aryl group was important for high affinity, as seen also in **5h** and **12d**.

**Table 1.** Inhibition of MAO activity.

compound	% inhibition at 10 μM^a^		IC_50_ (μM)^b^	
	hMAO-A	hMAO-B	hMAO-A	hMAO-B
**3a**	−6	38	none	5.28 ± 1.44 (40%)
**3b**	10	34		
**3c**	−3	10		
**3d**	0	27		
**4b**	6	31		
**4c**	0	27		
**4d**	0	28		
**5a**	19	55	6.2 ± 0.95	10.0 ± 1.1
**5b**	6	36	none	83
**5d**	71	70	0.27 ± 0.06	0.75 ± 0.18
**5e**	10	19		
**5g**	22	36	4.34 ± 1.31	11.0 ± 4.0
**5h**	70	72	0.31 ± 0.11	0.44 ± 0.1
**6**	none	12	(rate increases)^c^	9.0 ± 2.3
**11**	15	19		>100
**13**	−8	23		
**12a**	6	26		
**12b**	16	16		>100
**12c**	5	21		
**12d**	27	52	1.41 ± 0.47 (32%)	3.62 ± 0.69
**14a**	5	40		6.84 ± 0.76 (77%)
**14b**	14	52		4.64 ± 0.41 (77%)
**14c**	8	44		6.75 ± 1.06 (73%)
**14d**	11	44		5.93 ± 1.02 (77%)
safinamide	11	90		0.08 [39]
harmine	100	17	0.009 ± 0.001 (100%)	11.6 ± 3.1

^a^Observed inhibition (% = 100 × rate with inhibitor/rate without inhibitor). Values are average of two independent experiments with less than 15% difference.

^b^IC_50_ values ± s.d. for two independent experiments with 22 points each. The maximum inhibition (%) is given in parentheses if less than 95%.

^c^This compound (**6**) is oxidized by MAO-A so is a substrate (*K*_M_ = 35.5 µM) inducing H_2_O_2_ production in the absence of tyramine.

There was no difference in the inhibition without or with 30 min preincubation of MAO with the compounds. This and the restoration of full activity upon dilution (×100) indicated that the inhibition was reversible. For both MAO-A (with **5h** or **12d**) and MAO-B (with **12d** or **14b**), increasing the substrate concentration in the assay increased the rate of reaction, suggesting competitive inhibition.

**Figure 3.**
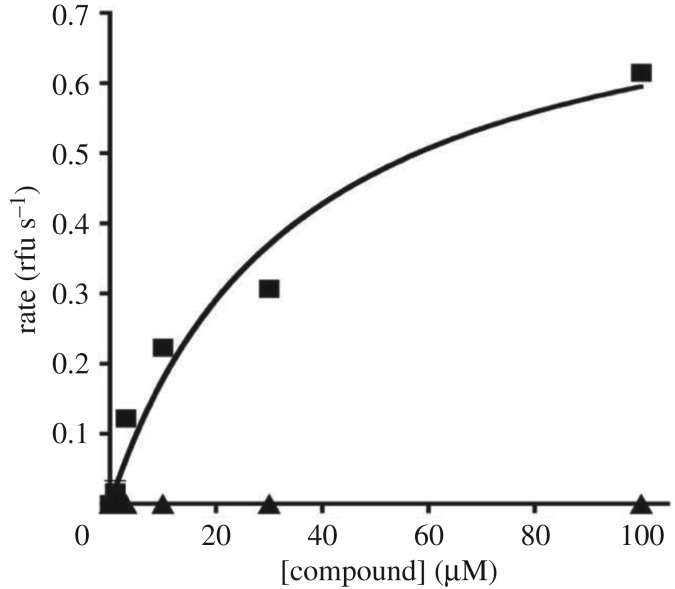
Oxidation of **6** (squares) but not **12d** (triangles) by MAO-A. The concentration of compound was varied in the standard assay coupled to the production of H_2_O_2_ but in the absence of the substrate tyramine.

Compound **6** in the screening assay with 10 µM inhibitor gave an increased rate of appearance of fluorescence indicating faster production of the product H_2_O_2_ (not the slower rate observed with other compounds that inhibit MAO activity). When incubated with MAO-A in the absence of the normal substrate, **6** (but not **12d**) gave a measurable rate of H_2_O_2_ production, as shown in figure 3. Thus, **6** binds to and is oxidized by MAO-A. The *V*_max_ for oxidation of **6** by MAO-A was 77% of the *V*_max_ for tyramine under the same conditions and the *K*_M_ was 35.5 ± 7.8 µM.

### 2.3. Molecular docking simulation

The energy minimum structures of human MAO-A and MAO-B after removing covalent ligands (clorgyline for 2BXR and deprenyl for 2BYB) were used as the models (electronic supplementary material, figure S2).

The MAO-A binding site formed of a single cavity extends from the flavin ring to the cavity-shaping loop consisting of residues 210–216. The volume of this cavity is estimated to be approximately 550 Å^3^ and is lined by aliphatic and aromatic residues, which are quite hydrophobic. In addition, two cysteine residues are located near the entry of the catalytic site that share in the interaction. The MAO-A active site lacks the MAO-B constriction allowing bulkier molecules to approach the aromatic cage of FAD, Tyr407 and Tyr444 [40].

The active site of hMAO-B is small in volume compared to MAO-A but shows the same hydrophobic nature. The MAO-B active site entrance is surrounded by hydrophobic amino acid residues (Leu171, Tyr326, Phe168, Ile198 and Ile199), followed by a narrow hydrophobic tunnel leading to the aromatic cage lined by the isoalloxazine of FAD, Tyr398 and Tyr435 [40].

**Figure 4.**
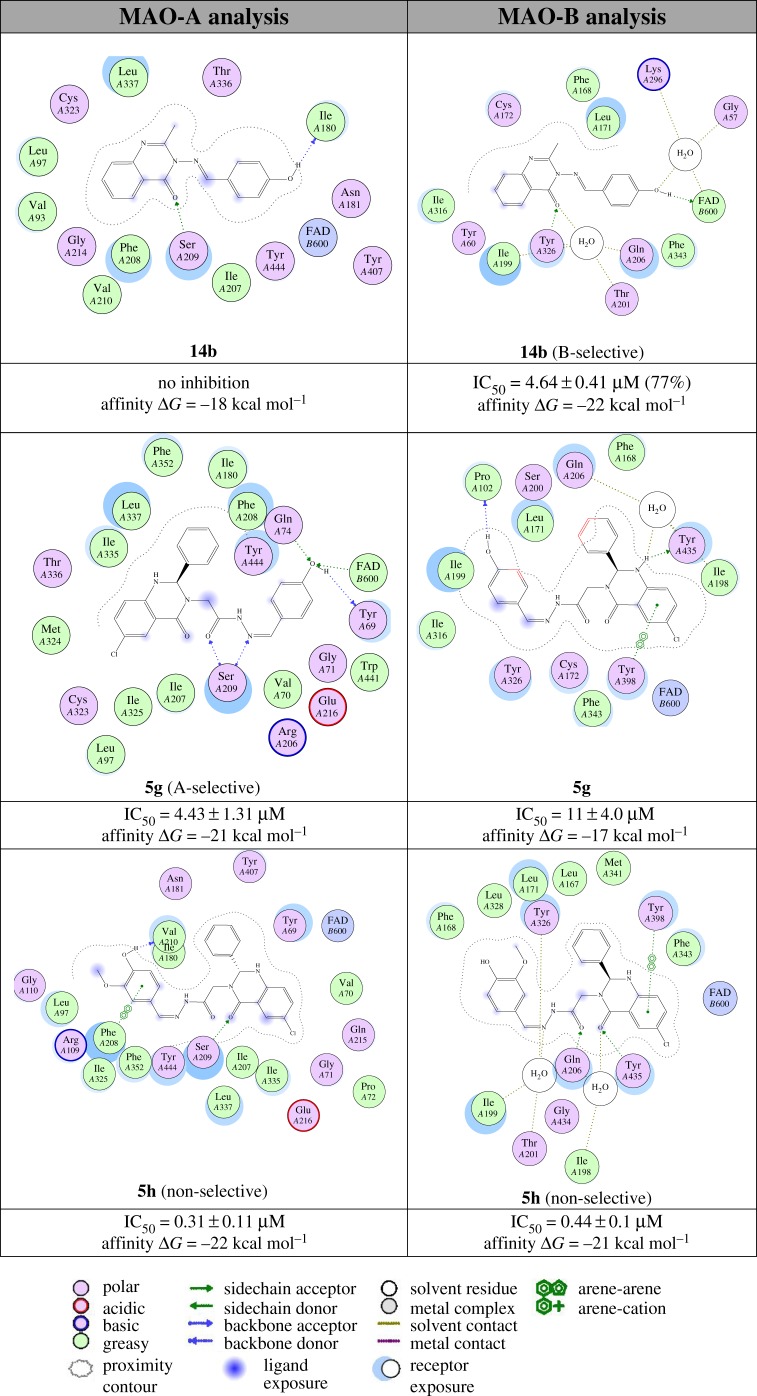
Interactions of selective and non-selective inhibitors with MAO-A and MAO-B.

The interactions of the phenylquinazoline head, acetohydrazide linker and aromatic tail with amino acid residues in the active sites of MAO-A and MAO-B are shown in figure 4 and listed in electronic supplementary material, table S1. The selective **14b** (E-form) derivative is well tolerated in the MAO-B pocket, binds close to the FAD and forms stable direct hydrogen bonds *via* the OH and indirectly to two residues Gln57 (2.3 Å) and Lys296 (2.5 Å) (figure 4, top). The scaffold binds to the residues Gln206, Tyr326, Thr201 and Ile199. The methyl group is well tolerated through hydrophobic interaction with Phe168 and Leu171. By contrast, the docking of **14b** (E-form) in the MAO-A formed unfavourable interactions in the pocket. The binding analysis of the MAO-A selective **5g** (Z-form) showed favourable interaction in the pocket with stable hydrogen bonding interactions through the terminal tail OH with Tyr69, FAD and Gln74 (figure 4, middle). It also binds through the acetohydrazide linker to the Ser209 by dual hydrogen bonds (2.72 and 2.48 Å). The phenylquinazoline head had hydrophobic interaction with the pocket formed of Phe352, Ile180, Leu337 and Phe208 residues. By contrast, MAO-B with **5g** (Z-form) showed serious clashes with both head and tail parts, and reversed placement compared to **14b**. The non-selective compound **5h** revealed the head phenylquinoxaline close to the FAD and good interaction behaviour towards the both targets (figure 4, bottom). All molecular placement data of active compounds within the binding pockets of MAO-A compared to MAO-B are summarized in table 2.

**Table 2.** Detailed docking data of representative compounds with MAO-A and MAO-B.

ID	fragment	MAO-A	MAO-B	interaction type
**14b**	methyl quinazoline head	hydrophobic interaction with terminal pocket formed of Gly214, Phe208, Val93, Leu97, Lys323 and Thr336	hydrophobic interaction with terminal pocket formed of Tyr80, Ile316, Cys172 and Ile199	hydrophobic and hydrogen bonding
		hydrogen bonding through the (*C=O*) to Ser209 with 2.2 Å	Bidirectional hydrogen bonding through the (*C=O*) to Tyr325 with 2.4 Å and H_2_O-Thr201, with 4.6 Å	
	Imino linker	Vdw interaction with terminal pocket formed of Ser209	Vdw interaction with terminal pocket formed of Leu171	Vdw interaction
	aromatic ring tail	aromatic stacking tolerated in cage formed of Phe208 and FAD	hydrophobic interaction with terminal pocket formed of Gly206 and Phe343	hydrophobic and hydrogen bonding
		hydrogen bonding through the (*p-*OH) to Ile180 with 2.7 Å	bidirectional hydrogen bonding through the (*p-OH*) to FAD with 2.6 Å and H_2_O-Gly57, with 4.5 Å	
**5g**	Phenyl dihydroquinazoline head	hydrophobic interaction with terminal pocket formed of Leu337, Ile207, Ile325 and Phe208	aromatic stacking tolerated in cage formed of Tyr398	hydrophobic and hydrogen bonding
			hydrogen bonding through (NH) fragment to Tyr435 with distance 2.3 Å	
	acetohydrazide linker	bidirectional hydrogen bonding through the (C=O) and (=N) to Ser209 with 1.95 and 2.20 Å	hydrophobic interaction with terminal pocket formed of Tyr326 and Cys172	hydrophobic and hydrogen bonding
	aromatic ring tail	bidirectional hydrogen bonding through the (*p-*OH) to Gly74 and Tyr69 with 1.85, 2.9 Å	hydrogen bonding through the (*p*-OH) to Pro102 with 2.51 Å	hydrophobic and hydrogen bonding
		hydrophobic interaction with terminal pocket formed of Gly71, Tyr69, Tyr444 and FAD	hydrophobic interaction with terminal residue Ile199, Leu171 and Ile316	
**5h**	Phenyl dihydroquinazoline head	hydrophobic interaction with terminal pocket formed of Tyr69, Ile207, Ile335, Tyr407 and Val70	hydrophobic interaction with terminal pocket formed of Phe343, Tyr435 and FAD	hydrophobic interaction
		hydrogen bonding through the (*C=O*) to Ser209 with 2.7 Å	hydrogen bonding through the (*C=O*) to Tyr435 with 2.3 Å and H_2_O-Ile198 with 4.9 Å	hydrophobic and hydrogen bonding
			aromatic stacking tolerated in cage formed of Tyr398	
	acetohydrazide linker	aromatic stacking tolerated in cage formed of Phe208	hydrogen bonding through the (*C=O*) to Gln206 with 3.1 Å	hydrophobic and hydrogen bonding
	aromatic ring tail	hydrogen bonding through the (*p-*OH) to Val210 with 2.4 Å	hydrophobic interaction with terminal pocket formed of Phe168, Leu328 and Tyr326	hydrophobic and hydrogen bonding
		Vdw interaction with terminal pocket formed of Leu97		

### 2.4. SAR analysis

MAO-A is inhibited only by the quinazoline compounds of type **5** (**5a**, **5d**, **5g**, **5h**) and with only micromolar potency, in contrast with our earlier 3-benzylquinoxaline-based compounds found to have nM potency [24]. For MAO-B, compounds **5a**, **5d**, **5g**, **5h** and **12d** gave full inhibition with micromolar affinity. The **14** series all gave 75% inhibition in the dose–response curves and IC_50_ values about 6 μM. None of the series **14** compounds inhibited MAO-A, so these are selective MAO-B inhibitors. Interestingly, MAO-A and MAO-B were equally inhibited by the precursor **11**, and by **12b**, **5d** and **5h**, all with substituted aryl groups as the aromatic tail.

Considering first the phenylquinazoline head shown in the design strategy in chart 1 (type **5** compounds), the addition of Cl to the head group had no effect. However, switching the 2-methyl in **12** to the 2-phenyl in **5** improved inhibition of both MAO-A and MAO-B activity, from no inhibition to IC_50_ values below 10 μM (table 1). Changing the linker from the acetylhydrazide found in **5** and **12** to the imine found in **14** introduced selectivity for MAO-B. Both **5a** and **14a** have IC_50_ values below 10 µM for MAO-B but **5a** inhibits MAO-A with an IC_50_ of 4.4 µM, whereas **14a** does not inhibit MAO-A. In the third region, the addition of 4-OH and 3-OCH_3_ to the aromatic tail improves the IC_50_ by more than 10-fold (**5a** with MAO-B is 10 µM, but **5d** is 0.75 µM; 6.2 and 0.27 µM for MAO-A). By contrast, with only the 4-OH, compounds **5b** and **5g** are poor inhibitors. This difference might be due to the changed interactions in the active site (figure 4 and table 2).

## 3. Conclusion

Prior work showed that the 3-benzylquinoxaline and quinazoline scaffolds gave a good basis for selective inhibition of MAO-A. Here, compounds were prepared to explore different synthetic methodologies and study the influence of the heterocyclic head group, the hydrazido linker and the aromatic tail in the inhibition. The N'-substituted benzylidene-2-(6-chloro-4-oxo-2-aryl-1,4-dihydroquinazolin-3(2H)-yl)aceto-hydrazide series provided the most potent inhibitors, namely **5d** (IC_50_ = 0.25 µM, threefold selective for MAO-A) and **5h** (0.31 µM on MAO-A and 0.44 µM on MAO-B). Removing the aryl tail substituents resulted in decreased inhibition (**5a** IC_50_ = 6 µM). The shorter less flexible linker in (*E*)-3-(substituted benzylideneamino)-2-methylquinazolin-4(3H)-one compounds **14a–14d** resulted in inhibition selective for MAO-B with IC_50_ values in the micromolar range. Molecular docking revealed the importance of interactions near the FAD for good affinity.

## 4. Experimental section

### 4.1. General chemistry

Melting points were determined with a Mel-Temp apparatus and are uncorrected. Nuclear magnetic resonance spectra (^1^H NMR and ^13^C NMR spectra) were recorded using a Bruker 400 MHz spectrometer with the chemical shift values reported in *δ* units (part per million) and the coupling constant (*J*) in hertz. Infrared data were obtained using a Perkin–Elmer 1600 series Fourier transform instrument as KBr pellets. Mass spectra were recorded on Brucker MALDI microflex and Agilent 6320 Ion Trap equipped with an electrospray ionization interface mass spectrometer. Elemental analyses were performed on Perkin–Elmer 2400 elemental analyser, and the values found were within ±0.3% of the theoretical values. The follow-up of the reactions and checking the purity of the compounds were made by TLC on silica gel-protected aluminium sheets (Type 60 GF254, Merck) and the spots were detected by exposure to UV-lamp at *λ* 254 nm for few seconds. Compounds of type **12** [31,32] and **14** were prepared according to the reported procedure [34–38] (electronic supplementary material).

#### 4.1.1. General procedure for the preparation of methyl (2-aminobenzoyl)-glycinate **(2a–2b)**

In round bottom flask (500 ml), a mixture of glycine methyl ester hydrochloride (1.26 g, 10 mmol), triethylamine (1 ml) was dissolved in ethanol (300 ml) and heated in water both at 60–70°C for 7 min, isatoic anhydride (10 mmol) was added to the mixture (portion wise) over a period of 30 min with stirring at 80°C. The mixture was allowed to heat under reflux for extra 30 min. The solvent was evaporated and the residue was used in the next step without further purification.

##### 4.1.1.1. Methyl (2-aminobenzoyl)glycinate **(2a)** [25]

Yield: 69%; mp 75–77°C.

##### 4.1.1.2. Methyl (2-amino-5-chlorobenzoyl)glycinate **(2b)**

Yield: 71%; mp 155–157°C. IR (KBr): 3240, 3182 (NH_2_ and NH), 1718 (C=O, ester), 1670 (C=O, amide) cm^−1^.

#### 4.1.2. General procedure for the preparation of methyl 2-(4-oxo-2-phenyl-1,2-dihydroquinazolin-3(4H)-yl)acetate **(3a–3d)**

Method A: To the solution of methyl (2-amino-5-substituted benzoyl)glycinate 2 (10 mmol) in ethanol (50 ml) was added the appropriate aldehydes and four drops of acetic acid at room temperature. The reaction mixture was heated under reflux for about 8 h and followed by TLC (EtOAc : MeOH 7 : 3). The solvent was removed under vacuum and the crude product was recrystallized from ethanol, filtered and washed with ethanol to afford the pure product.

Method B: Isatoic anhydride (6.13 mmol), triethylamine (0.619 g, 6.13 mmol), glycine methyl ester hydrochloride (0.769 g, 6.13 mmol), the appropriate aldehyde (6.13 mmol) and CuO nanoparticles (0.042 g, 0.613 mmol) were mixed in aqueous ethanol (EtOH : H_2_O 15 : 5) (20 ml). The reaction mixture was heated under reflux for 8 h and followed by TLC (EtOAc : MeOH 7 : 3). The CuO nanoparticle catalyst was separated by filtration and the filtrate was concentrated and left to cool to room temperature. The product that precipitated out was filtered off and recrystallized from ethanol.

##### 4.1.2.1. Methyl-2-(4-oxo-2-phenyl-1,2-dihydroquinazolin-3(4H)-yl)acetate **(3a)**

Method A: yield: 79%; Method B: yield: 83%; mp 198–200°C [25]

##### 4.1.2.2. Methyl-2-(6-chloro-4-oxo-2-phenyl-1,2-dihydroquinazolin-3(4H)-yl)acetate **(3b)**

Method A: yield: 77%; Method B: yield: 91%: mp 162–163°C; IR (KBr): 3375 (NH), 1750 (C=O, ester), 1630 (C=O, amide) cm^−1^; ^1^H NMR (400 MHz: DMSO-d_6_): *δ* 3.60 (s, 3H, OCH_3_), 3.63 (d, 1H, J 17.2 Hz, α-CH), 4.41 (d, 1H, J 17.2 Hz, α-CH), 5. 98 (s, 1H, sp^3^ C-H), 6.74 (d, 1H, J 8 Hz, Ar-H), 7.31 (d, 1H, J 8 Hz, Ar-H), 7.40 (bs, 5H, Ar-H), 7.59 (bs, 1H, NH), 7.63 (s, 1H, Ar-H). ^13^C NMR (100 MHz: DMSO- d_6_): *δ* 46.32, 52.36, 71.95, 115.43, 116.89, 121.37, 127.07, 127.32, 129.23, 129.59, 133.99, 139.81, 146.39, 162.23, 169.46. Anal. Calcd for C_17_H_15_ClN_2_O_3_: C, 61.73; H, 4.57; N, 8.47. Found: C, 62.46; H, 4.61; N, 8.45.

##### 4.1.2.3. Methyl 2-(6-chloro-2-(4-methoxyphenyl)-4-oxo–1,2-dihydroquinazolin-3(4H)-yl)acetate **(3c)**

Method B: yield: 55%: mp 148–150°C. IR (KBr): 3300 (NH), 1710 (C=O, ester), 1630 (C=O, amide) cm^−1^. ^1^H NMR (400 MHz: DMSO-d_6_): *δ* 3.58 (d, 1H, J 17.2 Hz, α-CH), 3.60 (s, 3H, OCH_3_), 3.75 (s, 3H, OCH_3_), 4.34 (d, 1H, J 17.2 Hz, α-CH), 5.92 (s, 1H, sp^3^ C-H), 6.75 (d, 1H, J 8.4 Hz, Ar-H), 6.96 (d, 2H, J 8.2 Hz, Ar-H), 7.32 (d, 1H, J 8.4 Hz, Ar-H), 7.34 (d, 2H, J 8.2 Hz, Ar-H), 7.53 (bs, 1H, NH), 7.58 (s, 1H, Ar-H). ^13^C NMR (100 MHz: DMSOd_6_): *δ* 46.00, 52.35, 55.66, 71.72, 114.53, 115.37, 116.90, 121.54, 127.04, 129.08, 131.41, 134.06, 146.75, 160.29, 162.45, 169.47. Anal. Calcd for C_18_H_17_ClN_2_O_4_: C, 59.92; H, 4.75; N, 7.76. Found: C, 60.01; H, 4.61; N, 7.55.

##### 4.1.2.4. Methyl 2-(6-chloro-2-(2-methoxyphenyl)-4-oxo–1,2-dihydroquinazolin-3(4H)-yl)acetate **(3d)**

Method B: yield: 55%: mp 152–154°C. IR (KBr): 3300 (NH), 1751 (C=O, ester), 1639 (C=O, amide) cm^−1^. ^1^H NMR (400 MHz: DMSO-d_6_): *δ* 3.60 (s, 3H, OCH_3_), 3.62 (d, 1H, J 17.2 Hz, α-CH), 3.79 (s, 3H, OCH_3_), 4.41 (d, 1H, J 17.2 Hz, α-CH), 6.22 (s, 1H, sp^3^ C-H), 6.76 (d, 1H, J 7.6 Hz, Ar-H), 6.93 (t, 1H, J 7.6 Hz, Ar-H), 7.07 (d, 1H, J 7.6 Hz, Ar-H), 7.20 (d, 1H, J 7.6 Hz, Ar-H), 7.27 (d, 1H, J 7.6 Hz, Ar-H), 7.28 (bs, 1H, NH), 7.34 (t, 1H, J 7.6 Hz, Ar-H), 7,58 (s, 1H, Ar-H). ^13^C NMR (100 MHz: DMSO-d_6_): *δ* 44.82, 51.04, 54.84, 65.44, 111.04, 114.05, 115.60, 119.79, 119.94, 125.85, 126.12, 126.24, 129.62, 132.60, 145.40, 156.09, 161.23, 168.43. Anal. Calcd for C_18_H_17_ClN_2_O_4_: C, 59.92; H, 4.75; N, 7.76. Found: C, 60.14; H, 4.73; N, 7.62.

#### 4.1.3. General procedures for the preparation of 2-(6-substituted-4-oxo-2-phenyl-1,2 dihydroquinazolin-3(4H)-yl)acetohydrazide **(4a–4d)**

To a solution of compound **3** (3.1 mmol) in methanol (10 ml), hydrazine hydrate (80%) (0.4 ml) was added. The reaction mixture was heated under reflux for 3 h and monitored by TLC (EtOAc : MeOH 7 : 3). The solution was concentrated under vacuum and the product that precipitated out was filtrated off, recrystallized from ethanol and dried.

##### 4.1.3.1. 2-(4-Oxo-2-phenyl-1,2-dihydroquinazolin-3(4H-yl)acetohydrazide **(4a)**

Yield 77%; mp 215–218°C [25].

##### 4.1.3.2. 2-(6-Chloro-4-oxo-2-phenyl-1,2-dihydroquinazolin-3(4H)-yl)acetohydrazide **(4b)**

Yield 75%; mp 184–185°C; IR (KBr): 3320, 3300 (NH_2_ and NH), 1675 (C=O, amide) cm^−1^. ^1^H NMR (400 MHz: DMSO-d_6_): *δ* 3.18 (d, J 16 Hz, 1H, α-CH), 4.20 (s, 2H, NH_2_), 4.5 (d, J 16 Hz, 1H, α-CH), 5.96 (s, 1H, sp3 CH) 6.72 (d, J 8 Hz, 1H, Ar-H), 7.28 (d, J 8 Hz, 1H Ar-H), 7.39 (bs, 5H Ar-H), 7.52 (s, 1H, NH), 7.59 (s, 1H, Ar-H), 9.052 (s, 1H, NHCO). ^13^C NMR (100 MHz: DMSO-d_6_): 45.62, 71.98, 115.72, 116.75, 121.22, 127.08, 127.27, 129.2, 129.50, 133.73, 140.02, 146.16, 162.24, 167.45. Anal. Calcd for C_16_H_15_ClN_4_O_2_: C, 58.10; H, 4.57; N, 16.94. Found: C, 57.95; H, 4.61; N, 16.81.

##### 4.1.3.3. 2-(6-Chloro-2-(4-methoxyphenyl)-4-oxo-1,2-dihydroquiazolin-3(4H)-yl)acetohydrazide **(4c)**

Yield 86%; mp 159–160°C; IR (KBr): 3310, 3230 (NH_2_ and NH), 1690 (C=O, amide) cm^−1^. ^1^H NMR (400 MHz: DMSO-d_6_): *δ* 3.56 (d, J 17.2, 1H, α-CH), 3.60 (s, 3H, NH_2_ and NH), 3.75 (s, 3H, OCH_3_), 4.34 (d, J 17.2 HZ, 1H, α-CH), 5.92 (s, 1H, sp^3^ CH), 6.72 (d, J 8.4 Hz, 1H, Ar-H), 6.96 (d, J 8 Hz, 2H, Ar-H), 7.31 (d, J 8.4 Hz, 1H Ar-H), 7.34 (d, J 8 Hz, 2H, Ar-H), 7.49 (bs, 1H, NHCO), 7.58 (s, 1H, Ar-H). ^13^C NMR (DMSO-d_6_, 100 MHz): *δ* 51.26, 54.60, 70.72, 113.47, 114.39, 115.83, 120.31, 126.02, 127.84, 130.36, 132.85, 145.53, 159.29, 161.34, 168.44. Anal. Calcd for C_17_H_17_ClN_4_O_3_: C, 56.59; H, 4.75; N, 15.53. Found: C, 56.35; H, 4.72; N, 15.65.

##### 4.1.3.4. 2-(6-Chloro-2-(2-methoxyphenyl)-4-oxo-1,2-dihydroquinazolin-3(4H)-yl)acetohydrazide **(4d)**

Yield 87%; mp 149–151°C. IR (KBr): 3310, 3200 (NH_2_ and NH), 1680 (C=O, amide) cm^−1^. ^1^H NMR (400 MHz: DMSO-d_6_): *δ* 3.60 (s, 3H, NH, NH_2_), 3.62 (d, J 17.2 Hz, 1H, α-CH), 3.79 (s, 3H, OCH_3_), 4.35 (d, J 17.2 Hz, 1H,α-CH), 6.22 (s, 1H, sp^3^ CH), 6.76 (d, J 8 Hz, 1H, Ar-H), 6.93 (t, 2H, J 7.2 Hz, Ar-H), 7.07 (d, J 8 Hz, 1H, Ar-H), 7.20 (d, J 7.2 Hz, 1H, Ar-H), 7.26–7.28 (m, 2H, Ar-H, NHCO), 7.34 (t, J 8 Hz, 1H, Ar-H), 7.57 (s, 1H, Ar-H). ^13^C NMR (DMSO-d_6_, 100 MHz): *δ* 52.30, 56.21, 66.75, 111.94, 115.13, 116.84, 120.86, 121.03, 126.91, 127.19, 127.31, 130.75, 133.80, 146.70, 157, 162.25, 169.41. Anal. Calcd for C_17_H_17_ClN_4_O_3_: C, 56.59; H, 4.75; N, 15.53. Found: C, 56.43; H, 4.75; N, 15.57.

#### 4.1.4. General procedures for the preparation of N'-substituted benzylidene-2-(4-oxo-2-phenyl-1,4-dihydroquinazolin-3(2H)-yl)acetohydrazide **(5a–5h)**

To a solution of compound **4** (0.25 g, 6.85 mmol) in ethanol (20 ml), the appropriate aldehyde (6.85 mmol) was added and the mixture was stirred for 24 h at temperature between 25 and 60°C and monitored by TLC. When the reaction is over, the mixture was concentrated and left to cool to room temperature. The product that precipitated out was filtrated off, recrystallized from ethanol and dried.

##### 4.1.4.1. N'-Benzylidene-2-(4-oxo-2-phenyl-1,2-dihydroquinazolin-3(4H)-yl)acetohydrazide **(5a)**

Yield: 88%; mp 223–225°C. IR (KBr): 3304 (NH), 1689, 1640 (C=O, amide) cm^−1^. Isomer E (70%): ^1^H NMR (400 MHz: DMSO-d_6_): *δ* 3.66 (d, 1H, J = 17.2 Hz, α-CH), 5.05 (d, 1H, J = 17.2 Hz, α-CH), 5.94 (s, 1H, sp^3^ C-H), 6.70–6.72 (m, 2H, Ar-H), 7.25–7.69 (m, 13H, Ar-H and NH), 7.93 (s, 1H, CH = N), 11.49 (s, 1H, NHCO). ^13^C NMR (100 MHz: DMSO-d_6_): *δ* 45.47, 72.22, 114.73, 114.79, 117.67, 127.19, 127.58, 128.11, 129.20, 129.29, 130.34, 133.94, 134.35, 140.29, 143.95, 147.04, 147.74, 163.56, 169.54. Isomer Z (30%): ^1^H NMR (400 MHz: DMSO-d_6_): *δ* 3.12 (d, 1H, J = 16.8 Hz, α-CH), 4.37 (d, 1H, J = 16.8 Hz, α-CH), 5.92 (s, 1H, sp^3^ C-H), 6.70–6.72 (m, 2H, Ar-H), 7.25–7.69 (m, 13H, Ar-H and NH), 8.15 (s, 1H, CH = N), 11.44 (s, 1H, NHCO). ^13^C NMR (100 MHz: DMSO-d_6_): *δ* 46.40, 72.33, 114.57, 114.68, 117.59, 127.40, 127.45, 127.50, 128.09, 129.17, 129.48, 130.50, 133.89, 134.06, 134.64, 147.53, 147,59, 163.59, 164.84. MS-MALDI: m/e 385.06 [M+1]. Anal. Calcd for C_23_H_20_N_4_O_2_: C, 71.86; H, 5.24; N, 14.57. Found: C, 71.02; H, 4.95; N, 14.41.

##### 4.1.4.2. N'-(4-Hydroxybenzylidene)-2-(4-oxo-2-phenyl-1,2-dihydroquinazolin-3(4H)-yl)acetohydrazide **(5b)**

Yield: 84%; mp 230–232°C. IR (KBr): 3275 (br) (OH and NH), 1685, 1650 (C=O, amide) cm^−1^. Isomer E (70%): ^1^H NMR (400 MHz: DMSO-d_6_): *δ* 3.60 (d, 1H, J 17.2 Hz, α-CH), 5.03 (d, IH, J 17.2 Hz, α-CH) 5.99 (s, 1H, sp^3^ C-H), 6.71, 6.78, 7.27, 7.37, 7.67 (m, d, m, d, d, 14H, Ar-H and NH) 7.82 (s, 1H, CHN), 9.89 (s, 1H, OH), 11.28 (s, IH, NHCO). ^13^C NMR (100 MHz: DMSO-d_6_): *δ* 45.43, 72.23, 114.71, 114.80, 116.14, 117.64, 125.38, 127.57, 128.10, 128.91, 129.20, 129.47, 133.92, 140.32, 144.24, 147.72, 159.65, 163.55, 169.18. Isomer Z (30%): ^1^H NMR (400 MHz: DMSO-d_6_): *δ* 3.33 (d, IH, *J*=16.4 Hz, α-CH), 4.52 (d, IH, *J*=17.2 Hz, α-CH), 5.99 (s, 1H, sp^3^ C-H), 6.71, 6.82, 7.27, 7.44, 7.51, (m, d, m, d, d, 14H, Ar-H and NH), 8.03 (s, 1H, CHN), 9.93 (s, 1H, OH), 11.21 (s, 1H, NHCO). ^13^C NMR (100 MHz: DMSO-d_6_): *δ* 46.30, 72.29, 114.60, 114.74, 117.64, 125.59, 127.43, 128.10, 129.20, 129.26, 129.45, 134.04, 140.32, 144.24, 147.35, 147.57, 159.83, 164.44, 169.18. MS-ESI: m/e 399.6 [M+1–2]. Anal. Calcd for C_23_H_20_N_4_O_3_: C, 68.99; H, 5.03; N, 13.99. Found: C, 68.75; H, 4.79; N, 13.61.

##### 4.1.4.3. N'-(4-Methoxybenzylidene)-2-(4-oxo-2-phenyl-1,2-dihydroquinazolin-3(4H)-yl)acetohydrazide **(5c)**

Yield: 91%; mp 210–212°C. IR (KBr): 3300, 3290 (NH), 1690 (C=O, amide) cm^−1^. Isomer E (70%): ^1^H NMR (400 MHz: DMSO-d_6_): *δ* 3.60 (d, 1H, J 17.2 Hz, α-CH), 5.03 (d, IH, J 17.2 Hz, α-CH) 5.99 (s, 1H, sp^3^ C-H), 6.71, 6.78, 7.27, 7.37, 7.67, (m, d, m, d, d,14H, Ar-H and NH) 7.82 (s, 1H, CHN), 9.89 (s, 1H, OH), 11.28 (s, IH, NHCO). ^13^C NMR (100 MHz: DMSO-d_6_): *δ* 45.43, 72.23, 114.71, 114.80, 116.14, 117.64, 125.38, 127.57, 128.10, 128.91, 129.20, 129.47, 133.92, 140.32, 144.24, 147.72, 159.65, 163.55, 169.18. Isomer Z (30%): ^1^H NMR (400 MHz: DMSO-d_6_): *δ* 3.33 (d, IH, J 16.4 Hz, α-CH), 4.52 (d, IH, J 16.4 Hz, α-CH), 5.99 (s, 1H, sp^3^ C-H), 6.71, 6.82, 7.27, 7.44, 7.51, (m, d, m, d, d, 14H, Ar-H and NH), 8.03 (s, 1H, CHN), 9.93 (s, 1H, OH), 11.21 (s, IH, NHCO). ^13^C NMR (100 MHz: DMSO-d_6_): *δ* 46.30, 72.29, 114.60, 114.74, 117.64, 125.59, 127.43, 128.10, 129.20, 129.26, 129.45, 134.04, 140.32, 144.24, 147.35, 147.57, 159.83, 164.44, 169.18. Anal. Calcd for C_24_H_22_N_4_O_3_: C, 69.55; H, 5.35; N, 13.52. Found: C, 70.05; H, 5.42; N, 13.65.

##### 4.1.4.4. N'-(4-Hydroxy-3-methoxybenzylidene)-2-(4-oxo-2-phenyl-1,2-dihydroquinazolin-3(4H)yl)acetohydrazide **(5d)**

Yield: 89%; mp 252–255°C. IR (KBr): 3300, 3295, 3200 (OH and NH), 1700 (C=O, amide) cm^−1^. Isomer E (64%): ^1^H NMR (400 MHz: DMSO-d_6_): *δ* 3.64 (d,1H, J 17.6 Hz, α-CH), 3.76 (s, 3H, OCH_3_), 4.03 (d, 1H, J 17.6 Hz, α-CH), 6.00 (s, 1H, sp^3^ C-H), 6.70, 6.69, 7.00, 7.25–7.49, 7.68 (m, d, d, s, m, d, 13H, ArH and NH), 7.82 (s, 1H, CHN), 9.50 (s, 1H, OH), 11.32 (s, 1H, NHCO). ^13^C NMR (100 MHz: DMSO-d_6_): *δ* 45.42, 55.98, 72.25, 110.06, 114.73, 114.82, 116.03, 117.69, 121.40, 125.81, 127.62, 128.12, 129.17, 129.21, 129.48, 133.93, 140.25, 144.39, 147.77, 148.33, 149.14, 163.58, 169.25. Isomer Z (36%): ^1^H NMR (400 MHz: DMSO-d_6_): *δ* 3.34 (d, 1H, J 16.4 Hz, α-CH), 3.81 (s, 3H, OCH_3_), 4.53 (d, 1H, J 16.4 Hz, α-CH), 6.00 (s, 1H, sp^3^ C-H), 6.70, 6.83, 7.06, 7.25–7.49, 7.68 (m, d, d, m, d, 13H, Ar-H + NH), 8.03 (s, 1H, CHN), 9.55 (s, 1H, OH), 11.25 (s, 1H, NHCO). ^13^C NMR (100 MHz: DMSO-d_6_): *δ* 46.32, 56.52, 72.35, 109.51, 114.62, 114.76, 115.88, 117.69, 122.41, 126.01, 127.62, 127.48, 128.12, 129.17, 129.21, 129.48, 134.06, 140.28, 147.60, 148.45, 149.38, 163.62, 164.49. MS-ESI: m/e 429.0 [M+1–2]. Anal. Calcd for C_24_H_22_N_4_O_4_: C, 66.97; H, 5.15; N, 13.02. Found: C, 67.05; H, 4.99; N, 13.14.

##### 4.1.4.5. N'-Benzylidene-2-(6-chloro-4-oxo-2-phenyl-1,4-dihydroquinazolin-3(2H)-yl)acetohydrazide **(5e)**

Yield: 92%; mp 266–270°C. IR (KBr): 3390, 3224 (NH), 1699, 1637 (C=O, amide) cm^−1^. Isomer E (73%): ^1^H NMR (400 MHz: DMSO-d_6_): *δ* 3.71 (d, 1H, J 17.2 Hz, α-CH), 5.04 (d, IH, J 17.2 Hz, α-CH), 6.02 (s, 1H, sp^3^ C-H), 6.75, 7.31, 7.27, 7.43, 7.57, 7,69 (d, d, m, m, d, 15H, Ar-H and NH), 7.99 (s, 1H, CHN), 11.51 (s, IH, NHCO). ^13^C NMR (100 MHz: DMSO-d_6_): *δ* 45.63, 72.11, 115.82, 116.79, 121.28, 127.20, 127.51, 129.26, 130.34, 133.73, 134.33, 139.96, 144.07, 146.45, 162.41, 169.27. Isomer Z (27%): ^1^H NMR (400 MHz: DMSO-d_6_): *δ* 3.63 (d, IH, I 16.4 Hz, α-CH), 4.52 (d, IH, J 16.42 Hz, α-CH), 6.02 (s, 1H, sp^3^ C-H), 6.75, 7.31, 7.27, 7.43, 7.57, 7,69 (d, d, m, m, d, 15H, Ar-H and NH), 8.15 (s, 1H, CH = N), 11.44 (s, 1H, NHCO). ^13^C NMR (100 MHz: DMSO-d_6_): *δ* 46.47, 72.23, 115.59, 116.79, 127.11, 127.40, 129.26, 129.59, 130.51, 133.85, 134.62, 146.32, 147.19, 162.44, 169.24. MS-ESI: m/e 417 [M+1–2]. Anal. Calcd for C_23_H_19_ClN_4_O_2_: C, 65.95; H, 4.57; N, 13.38. Found: C, 66.02; H, 4.70; N, 13.52.

##### 4.1.4.6. 2-(6-Chloro-4-oxo-2-phenyl-1,4-dihydroquinazolin-3(2H)-yl)-N'-(4-methoxybenzylidene)-acetohydrazide **(5f)**

Yield: 90%; mp 138–140°C. IR (KBr): 3290, 3275 (NH), 1695, 1650 (C=O, amide) cm^−1^. Isomer E (67%): ^1^H NMR (400 MHz: DMSO-d_6_): *δ* 3.68 (d, 1H, J 17.2 Hz, α-CH), 3.77 (s, 3H, CH_3_O), 5.03 (d, 1H, J 17.2 Hz, α-CH), 6.03 (s, 1H, sp^3^ C-H), 6.76 (d, 1H, J 8.8 Hz, ArH), 6.95 (d, 2H, J 8.4 Hz, ArH), 7.31 (dd, 1H, J 8.4 Hz, 2.4 Hz, ArH), 7.38–7.64 (m, 9H, ArH and NH), 8.09 (s, 1H, CH = N), 11.32 (s, 1H, NHCO). ^13^C NMR (100 MHz: DMSO-d_6_): *δ* 45.63, 55.10, 72.12, 114.74, 115.83, 116.78, 121.28, 126.94, 127.13, 127.52, 128.79, 129.26, 133.73, 139.96, 143.95, 146.45, 161.11, 162.42, 164.30, 169.02. Isomer Z (33%): ^1^H NMR (400 MHz: DMSO-d_6_): *δ* 3.41 (d, 1H, J 16.4 Hz, α-CH), 3.80 (s, 3H, OCH_3_), 4.52 (d, 1H, J 16.4 Hz, α-CH), 6.03 (s, 1H, sp^3^ C-H), 6.95 (d, 1H, J 8.4 Hz, ArH), 7.00 (d, 2H, J 8.4 Hz, ArH), 7.31 (dd, 1H, J 8.4 Hz, 2.4 Hz, ArH), 7.38–7.64 (m, 9H, ArH and NH), 7.88 (s, 1H, CH = N), 11.39 (s, 1H, NHCO). ^13^C NMR (100 MHz: DMSO-d_6_): *δ* 46.41, 56.53, 72.22, 114.74, 115.61, 116.81, 121.28, 126.94, 127.10, 127.40, 129.12, 129.60, 133.84, 139.96, 146.32, 147.08, 161.28, 162.45, 164.33, 169.02. Anal. Calcd for C_24_H_21_ClN_4_O_3_: C, 64.21; H, 4.72; N, 12.48. Found: C, 63.98; H, 4.84; N, 12.67.

##### 4.1.4.7. 2-(6-Chloro-4-oxo-2-phenyl-1,4-dihydroquinazolin-3(2H)-yl)-N'-(4-hydroxybenzylidene)acetohydrazide **(5g)**

Yield: 84%; mp 236–240°C. Isomer E (71%): ^1^H NMR (400 MHz: DMSO-d_6_): *δ* 3.64 (d, 1H, J 17.2 Hz, α-CH), 5.01 (d, 1H, J 17.2 Hz, α-CH), 6.01 (s, 1H, sp^3^ C-H), 6.74 (d, 1H, J 8.8 Hz, ArH), 6.78 (d, 2H, J 8.0 Hz, ArH), 7.31 (dd, 1H, J 8.8 Hz, 2.4 Hz, ArH), 7.37–7.57 (m, 9H, ArH and NH), 7.60 (d, 1H, J 2.4 Hz, ArH), 9.90 (br s, 1H, OH), 11.31 (s, 1H, NHCO). ^13^C NMR (100 MHz: DMSO-d_6_): *δ* 45.61, 72.07, 115.81, 116.14, 116.77, 121.24, 125.35, 127.10, 127.49, 128.94, 129.27, 133.73, 140.00, 144.35, 146.43, 159.68, 162.37, 164.15, 168.89. Isomer Z (29%): ^1^H NMR (400 MHz: DMSO-d_6_): *δ* 3.35 (d, 1H, J = 16.0 Hz, α-CH), 4.50 (d, 1H, J 16.0 Hz, α-CH), 6.01 (s, 1H, sp^3^ C-H), 6.78 (d, 1H, J 8.0 Hz, ArH), 6.82 (d, 2H, J 8.4 Hz, ArH), 7.31 (dd, 1H, J 8.8 Hz, 2.4 Hz, ArH), 7.37–7.57 (m, 9H, ArH and NH), 7.60 (d, 1H, J 2.4 Hz, ArH), 9.90 (br s, 1H, OH), 11.23 (s, 1H, NHCO). ^13^C NMR (100 MHz: DMSO-d_6_): *δ* 46.37, 72.15, 115.61, 116.14, 116.80, 121.24, 125.55, 127.25, 127.37, 129.27, 129.59, 133.84, 139.97, 146.29, 147.45, 159.85, 162.40, 164.15, 168.89. Anal. Calcd for C_23_H_19_ClN_4_O_3_: C, 63.52; H, 4.40; N, 12.88. Found: C, 63.76; H, 4.33; N, 13.15.

##### 4.1.4.8. 2-(6-Chloro-4-oxo-2-phenyl-1,4-dihydroquinazolin-3(2H)-yl)-N'-(4-hydroxy-3-methoxybenzylidene)-acetohydrazide **(5h)**

Yield: 93%; mp 240–242°C. Isomer E (67%): ^1^H NMR (400 MHz: DMSO-d_6_): *δ* 3.67 (d, 1H, J 17.6 Hz, α-CH), 3.76 (s, 3H, CH_3_O), 5.01 (d, 1H, J 17.6 Hz, α-CH), 6.01 (s, 1H, sp^3^ C-H), 6.74 (d, 1H, J 8.8 Hz, ArH), 6.78 (d, 1H, J 8.0 Hz, ArH), 6.99 (dd, 1H, J 8.4 Hz, 1.2 Hz, ArH), 7.11 (d, 1H, J 1.2 Hz, ArH), 7.31 (dd, 1H, J 8.8 Hz, 2.4 Hz, ArH), 7.36–7.60 (m, 8H, ArH and NH), 9.52 (br s, 1H, OH), 11.33 (s, 1H, NHCO). ^13^C NMR (100 MHz: DMSO-d_6_): *δ* 45.58, 55.98, 72.10, 110.09, 115.82, 116.02, 116.78, 121.26, 122.41, 125.77, 127.10, 127.54, 129.24, 129.60, 133.73, 139.92, 144.50, 146.47, 147.68, 148.33, 149.17, 162.40, 164.19, 168.95. Isomer Z (23%): ^1^H NMR (400 MHz: DMSO-d_6_): *δ* 3.35 (d, 1H, J 16.4 Hz, α-CH), 3.81 (s, 3H, CH_3_O), 4.50 (d, 1H, J 16.4 Hz, α-CH), 6.01 (s, 1H, sp^3^ C-H), 6.74 (d, 1H, J 8.8 Hz, ArH), 6.82 (d, 1H, J 8.0 Hz, ArH), 6.99 (dd, 1H, J 8.4 Hz, 1.2 Hz, ArH), 7.26 (d, 1H, J 1.2 Hz, ArH), 7.31 (dd, 1H, J 8.8 Hz, 2.4 Hz, ArH), 7.36–7.60 (m, 8H, ArH and NH), 9.52 (br s, 1H, OH), 11.25 (s, 1H, NHCO). ^13^C NMR (100 MHz: DMSO-d_6_): *δ* 46.37, 55.96, 72.20, 109.55, 115.62, 115.87, 116.81, 121.42, 122.41, 125.97, 127.10, 127.40, 129.29, 129.60, 133.84, 139.92, 144.50, 146.32, 147.68, 148.44, 149.40, 162.44, 164.19, 168.95. Anal. Calcd for C_24_H_21_ClN_4_O_4_: C, 62.00; H, 4.55; N, 12.05. Found: C, 61.81; H, 4.65; N, 12.13.

#### 4.1.5. 2-(((E)-benzylidene)amino)-5-chloro-N-(2-(2-((E)-4-hydroxy-3-methoxybenzylidene)hydrazinyl)-2-oxoethyl)benzamide **(6)**

Yield: 36%; mp 257–259°C. ^1^H NMR (400 MHz: DMSO-d_6_): *δ* 3.64 (s, 2H, CH_2_), 3.86 (s, 3H, CH_3_O), 6.99 (d, 1H, J 7.2 Hz, ArH), 7.18 (d, 1H, J 8.8 Hz, ArH), 7.30 (s, 1H, ArH), 7.36 (d, 1H, J 7.2 Hz, ArH), 7.42 (s, 1H, ArH), 7.55–7.60 (m, 6H, ArH), 7.63 (s, 1H, ArH), 7.71 (s, 1H, ArH), 8.74 (s, 1H, NHCH_2_), 8.80 (s, 1H, NHCO), 10.53 (s, 1H, OH). ^13^C NMR (100 MHz: DMSO-d_6_): *δ* 44.75, 56.05, 110.85, 116.05, 116.08, 123.10, 123.52, 124.63, 124.90, 127.53, 128.34, 130.40, 132.52, 136.62, 148.53, 148.58, 151.23, 151.83, 161.26, 166.88, 167.29, 171.30. Anal. Calcd for C_24_H_21_ClN_4_O_4_: C, 62.00; H, 4.55; N, 12.05. Found: C, 62.21; H, 4.71; N, 12.30.

#### 4.1.6. Methyl 2-(4-oxo-2-phenylquinazolin-3(4H)-yl)acetate **(8)**

To a solution of compound 3a (0.75 g, 2.54 mmol) in ethyl acetate (50 ml), 2,3-dichloro-5,6-dicyano-p-benzoquinone DDQ (0.691 g, 3.048 mmol) was added. The reaction mixture was stirred for 3 h, concentrated, and left to cool to room temperature. The product was purified by column chromatography using silica gel as stationary phase and ethyl acetate–methanol (8 : 2) as mobile phase. Yield: 89%; mp 115–116°C [Lit. [29] 150°C]. IR (KBr): 1750 (C=O, ester), 1688 (C=O, amide) cm^−1^. ^1^H NMR (400 MHz: DMSO-d_6_): *δ* 3.64 (s, 3H, OCH_3_), 4.66 (s, 2H,CH_2_), 7.57 (bs, 5H, Ar-H), 7.62 (t, 1H, J 8 Hz, Ar-H), 7.74 (d, 1H, J 8 Hz, Ar-H), 7.91 (t, 1H, J 8 Hz, Ar-H), 8.2 (d, 1H, J 8 Hz, Ar-H). ^13^C NMR (100 MHz: DMSO-d_6_): *δ* 47.86, 61.82, 120.32, 126.72, 127.90, 128.40, 129.17, 130.62, 134.95, 135.54, 147.35, 155.94, 161.62, 168.36, 168.87.

#### 4.1.7. 2-(4-oxo-2-phenylquinazolin-3(4H)-yl)acetohydrazide **(7)**

To a solution of compound **8** (0.294 g, 1 mmol) in methanol (10 ml), hydrazine hydrate (80%) (0.4 ml) was added. The reaction mixture was heated under reflux for 3 h and monitored by TLC (EtOAc : MeOH 7 : 3). The solution was concentrated and the product that precipitated out was filtrated off, recrystallized from ethanol and dried. Yield: 87%; mp 215–218°C. IR (KBr): 3350, 3300 (NH_2_ and NH), 1690 (C=O, amide) cm^−1^.

#### 4.1.8. Methyl 2-(2-methyl-4-oxoquinazolin-3(4H)-yl)acetate **(10)**

A mixture of compound **9** (1.03 g, 3 mmol), and triethylamine (0.636 g, 3 mmol), glycine methyl ester hydrochloride (0.803 g, 3 mmol). The reaction mixture was heated in an oil-bath at temperature of 120°C for 1 h, and the reaction was followed by TLC (EtOAc : MeOH 7 : 3). The crude residue was recrystallized from methanol and dried. Yield: 46%; mp 115–117°C [lit. 115°C [35]].

#### 4.1.9. 2-(2-Methyl-4-oxoquinazolin-3(4H)-yl)acetohydrazide **(11)**

To a solution of compound **10** (0.5 g, 2.15 mmol) in dimethylformamide (10 ml), hydrazine hydrate (80%, 0.25 ml) and two drops of anhydrous acetic acid were added. The reaction mixture was heated under reflux for 5 h and followed by TLC (EtOAc : MeOH 7 : 3). The reaction mixture was left to cool to room temperature and the product that separated out was filtered off and recrystallized from ethanol, yield= (79%), mp 256°C decomp. [lit. [35] 260°C]. IR (KBr): 3267, 3142 (NH_2_ and NH), 1670 (C=O, amide) cm^-1^. ^1^H NMR (400 MHz: DMSO-d_6_): *δ* 2.50 (s,3H,CH_3_), 3.41 (bs, 2H, NH_2_), 4.38 (s, 2H, CH_2_), 7.49 (t, 1H, J 7.6 Hz, Ar-H), 7.61 (d, J 7.6 Hz, 1H, Ar-H), 7.81 (t, J 7.6 Hz, 1H, Ar-H), 8.08 (d, J 7.6 Hz, 1H, Ar-H), 8.83, 9.44 (s, bs, 1H, NH). ^13^C NMR (100 MHz: DMSO-d_6_): *δ* 22.26, 44.20, 119.13, 125.56, 125.65, 125.82, 133.83, 146.51, 154.77, 160.50, 165.70.

#### 4.1.10. General procedures for the preparation of N'-substituted benzylidene-2-(2-methyl-4-oxoquinazolin-3(4H)-yl)acetohydrazide **(12a–12d)**

To a solution of compound **11** (1.624 g, 7.00 mmol) in dimethylformamide (10 ml), the appropriate aldehyde (7.00 mmol) and two drops of anhydrous acetic acid were added. The reaction mixture was heated under reflux for 5 h and the mixture was left to cool to room temperature. The product that precipitated out was filtrated off, recrystallized from ethanol and dried.

##### 4.1.10.1. N'-Benzylidene-2-(2-methyl-4-oxoquinazolin-3(4H)-yl)acetohydrazide **(12a)**

Yield: 64%; mp 242–245°C [lit. [32] 245°C]. IR (KBr): 3223 (NH), 1680 (C=O, amide) cm^−1^. Isomer E (80%): ^1^H NMR (400 MHz: DMSO-d_6_): *δ* 2.56 (s, 3H, CH_3_), 5.33 (s, 2H, CH_2_), 7.46–8.12 (m, 10H, Ar-H), 11.85 (s, 1H, NH). ^13^C NMR (100 MHz: DMSOd_6_): *δ* 23.31, 45.70, 120.08, 126.67, 126.84, 127.06, 127.45, 129.31, 130.59, 134.35, 135.01, 144.93, 147.66, 155.91, 161.69, 169.73. Isomer Z (20%): ^1^H NMR (400 MHz: DMSO-d_6_): *δ* 2.51 (s, 3H, CH_3_), 4.90 (s, 2H, CH_2_), 7.46–8.25 (m, 10H, Ar-H), 11.90 (s, 1H, NH). ^13^C NMR (100 MHz: DMSOd_6_): *δ* 23.46, 46.10, 120.10, 126.63, 126.87, 127.06, 127.62, 129.31, 130.66, 134.49, 135.04, 147.61, 147.75, 155.86, 164.00. MS-ESI: m/e 321.1 [M+1] (calcd for C_18_ H_16_N_4_O_2_).

##### 4.1.10.2. N'-(4-Hydroxybenzylidene)-2-(2-methyl-4-oxoquinazolin-3(4H)-yl)acetohydrazide **(12b)**

Yield: 86%; mp 192–195°C. IR (KBr): 3210, 3100 (br.) (OH and NH), 1680 (C=O, amide) cm^-1^. Isomer E (77%): ^1^H NMR (400 MHz: DMSO-d_6_): *δ* 2.56 (s, 3H, CH_3_), 5.30 (s, 2H, CH_2_), 6.83–6.87, 7.49–7.65, 7.80–7.84, 7.99–8.10–8.14 (m, m, t, s, m, 9H, Ar-H), 9.97 (s, 1H, OH), 11.65 (s, 1H, NH). ^13^C NMR (100 MHz: DMSO-d_6_): *δ* 23.30, 45.66, 116.18, 120.09, 125.38, 126.67, 126.80, 127.04, 129.20, 134.97, 145.21, 147.66, 155.93, 159.89, 161.69, 168.32. Isomer Z (23%): ^1^H NMR (400 MHz: DMSO-d_6_): *δ* 2.51 (s, 3H, CH_3_), 4.88 (s, 2H, CH_2_), 6.83–6.87, 7.49–7.65, 7.80–7.84, 7.99–8.10–8.14 (m, m, t, s, m, 9H, Ar-H), 9.97 (s, 1H, OH), 11.70 (s, 1H, NH). ^13^C NMR (100 MHz: DMSO-d_6_): *δ* 23.45, 46.08, 116.18, 120.12, 125.45, 126.64, 126.84, 129.40, 135.00, 147.61, 148.04, 155.87, 159.99, 163.57. MS-ESI: m/e 337.0 [M+1] (calcd for C_18_H_16_ N_4_O_3._). Anal. Calcd for C_18_H_16_N_4_O_3_: C, 64.28; H, 4.79; N, 16.66. Found: C, 64.50; H, 5.02; N, 16.84.

##### 4.1.10.3. N'-(4-Methoxybenzylidene)-2-(2-methyl-4-oxoquinazolin-3(4H)-yl)acetohydrazide **(12c)**

Yield: 77%; M.P.: >229°C decomp. [lit. [32] 230°C] IR (KBr): 3400 (NH), 1690 (C=O, amide) cm^−1^. Isomer E (78%): ^1^H NMR (400 MHz: DMSO-d_6_): *δ* 2.52 (s, 3H, CH_3_), 3.81 (s, 3H, OCH_3_), 5.31 (s, 2H, CH_2_), 7.02–7.07, 7.49–7.53, 7.62–7.71, 7.81–7.83, 8.03–8.19 (t, t, d, d, d, s, d, s, 9H, Ar-H), 11.72 (s, 1H, NH). ^13^C NMR (100 MHz: DMSOd_6_): *δ* 23.30, 45.67, 55.77, 114.80, 120.09, 126.66, 126.82, 127.04, 129.06, 130.40, 134.99, 144.80, 147.66, 155.93, 161.06, 161.31, 161.69, 163.63. Isomer Z (22%): ^1^H NMR (400 MHz: DMSO-d_6_): *δ* 2.56 (s, 3H, CH_3_), 3.83 (s, 3H, OCH_3_), 4.88 (s, 2H, CH_2_), 7.02–7.07, 7.49–7.53, 7.62–7.71, 7.81–7.83, 8.03–8.19 (t, t, d, d, d, s, d, s, 9H, Ar-H), 11.77 (s, 1H, NH). ^13^C NMR (100 MHz: DMSOd_6_): *δ* 23.40, 46.03, 55.84, 114.86, 120.12, 126.63, 126.85, 126.94, 129.23, 130.40, 135.02, 144.80, 147.61, 155.88, 161.06, 161.39, 162.14, 168.44. MS-ESI: m/e 350.7 [M+1].

##### 4.1.10.4. N'-(4-Hydroxy-3-methoxybenzylidene)-2-(2-methyl-4-oxoquinazolin-3(4H)-yl)acetohydrazide **(12d)**

Yield: 72%; mp 258–260°C. IR (KBr): 3294 (br), 3180 (br) (OH and NH), 1699, 1686 (C=O, amide) cm^-1^. Isomer E (81%): ^1^H NMR (400 MHz: DMSO-d_6_): *δ* 2.61 (s, 3H, CH_3_), 3.84 (s, 3H, OCH_3_), 5.31 (s, 2H, CH_2_), 6.85–8.23 (m, 8H, Ar-H), 9.47 (s, 1H, OH), 11.68 (s, 1H, NH). ^13^C NMR (100 MHz: DMSOd_6_): *δ* 23.32, 45.29, 110.0 7, 116.01, 120.08, 120.21, 121.98, 125.78, 126.63, 126.75, 126.86, 127.06, 134.89, 145.36, 147.58, 148.45, 149.42, 155.91, 166.79, 168.23. Isomer Z (19%): ^1^H NMR (400 MHz: DMSO-d_6_): *δ* 2.59 (s, 3H, CH_3_), 3.81(s, 3H, OCH_3_), 4.88 (s, 2H, CH_2_), 6.85–8.23 (m, 8H, Ar-H), 9.58 (s, 1H, OH), 11.76 (s, 1H, NH). ^13^C NMR (100 MHz: DMSOd_6_): *δ* 23.18, 45.73, 109.65, 115.91, 120.17, 122.49, 125.86, 126.65, 126.70, 126.83, 126.99, 135.00, 147.55, 148.45, 149.54, 166.15, 167.79. MS-ESI: m/e 336.4 [M+1]. Anal. Calcd for C_19_ H_18_ N_4_O_4_: C, 62.29; H, 4.95; N, 15.29. Found: C, 62.17; H, 5.01; N, 15.43.

### 4.2. MAO activity screening

The activity of MAO was determined using the coupled assay method of [41,42] in which the product, H_2_O_2_, acts with horseradish peroxidase to convert 10-acetyl-3,7-dihydroxyphenoxazine to the fluorescent resorufin. All compounds were screened for inhibition of the activity of membrane-bound human MAO-A and B at 10 μM in the presence of 2 × *K*_m_ tyramine (an assay concentration of 0.8 mM with MAO-A and 0.32 mM with MAO-B). For compounds that gave more than 30% inhibition, the IC_50_ was determined using seven concentrations of each compound (0.01–300 μM) with substrate at 2 × *K*_M_. The IC_50_ values are the mean and standard deviation for two separate experiments (16 and 22 points, respectively). The data were analysed using the equation for a three-parameter curve in Graphpad PRISM v. 4, and the % inhibition calculated from the top and bottom of the resulting curve.

#### 4.3. Docking studies

Molecular docking studies were carried out to understand the molecular binding modes of the active synthesized compounds towards two different biological targets the human MAO enzymes. The Protein Data Bank (PDB) crystallographic structures of human MAO-A (PDB ID: 2BXR) and human MAO-B (PDB ID: 2BYB) were prepared by the removal of the co-crystallized ligands [42]. Before screening the new compounds, the docking protocol was validated by running the simulation using these ligands, and low RMSD between docked and crystal conformations was obtained. AutoDock 3.0 [43] and MOE [44] softwares were used for all docking calculations. The AutoDockTools package was employed to generate the docking input files and to analyse the docking results. A grid box size of 90 × 90 × 90 points with a spacing of 0.375 Å between the grid points was generated that covered almost the entire protein surface. All non-polar hydrogens and crystallographic water molecules were removed prior to the calculations. The docking grid was centred on the mass centre of the bound drugs. Ligands were fully flexibly docked. In each case, 100 docked structures were generated using genetic algorithm searches. A default protocol was applied with an initial population of 50 randomly placed conformations, a maximum number of 2.5 × 10^5^ energy evaluations and a maximum number of 2.7 × 10^4^ generations. Heavy atom comparison root mean square deviations (RMSD values) were calculated and initial ligand binding modes were plotted. Protein–ligand interaction plots were generated using MOE 2012 [45].

## Supplementary Material

Supplementary Information

Reviewer comments

## Data accessibility

Our spectral data are attached with the submission as suggested by the editor Dr Laura Smith.
