# Supplementary material for: Design, synthesis, molecular modelling and in vitro screening of monoamine oxidase inhibitory activities of novel quinazolyl hydrazine derivatives
Source: R Soc Open Sci. 2020 Apr 22;7(4):200050. doi: 10.1098/rsos.200050 (PMC7211837; doi:10.1098/rsos.200050)
Supplement: Supplementary Information [file rsos200050supp1.docx]

**SUPPLEMENTARY INFORMATION FROM "Design, Synthesis, Molecular Modeling and in Vitro Screening of Monoamine Oxidase Inhibitory Activities of Novel Quinazolyl hydrazine Derivatives"**

Adel Amer^a,b^ *, Abdelrahman H. Hegazi**^a,b^**, Mohammed Khalil Alshekh^a^, Hany Emary Ali Ahmed^c,d^, Saied M. Soliman**^b,e^** , Antonin Maniquet^f^ and Rona R. Ramsay^f^ *

^a^Department of Chemistry, Faculty of Science, Taibah University, Saudi Arabia

^b^Department of Chemistry, Faculty of Science, Alexandria University, Alexandria 21321, Egypt

^c^Pharmacognosy and Pharmaceutical Chemistry Department, Pharmacy College, Taibah University, Al-Madinha Al-Munawaraha, Saudi Arabia

^d^Pharmaceutical Organic Chemistry Department, Faculty of Pharmacy, Al-Azhar University, Cairo, Egypt

^e^Department of Chemistry, College of Science & Arts, King Abdulaziz University, P.O. Box 344, Rabigh 21911, Saudi Arabia

^f^Biomedical Sciences Research Complex, University of St Andrews, Biomolecular Sciences Building, North Haugh, St Andrews KY16 9ST, UK

**Royal Society Open Science Journal**

**3-Amino-2-methylquinazolin-4(3H)-one 13.** A mixture of 2-methyl/propyl-4H-3,1-benzoxazin-4-one (1.750 g, 10 mmol) and 80% hydrazine hydrate (1.50 g, 10mmol) in methanol was refluxed for 3 hours and cooled. The separated solid was recrystallized from ethanol. Yield (79%); mp 138-140 °C [Lit.^33^ 139-141°C]. IR (KBr): 3325, 3210 (NH_2_), 1690 (C=O, amide) cm^-1^. ^1^H NMR (400 MHz: CDCl_3_): δ 2.70 (s, 3H, CH_3_), 4.94 (bs, 2H, NH_2_), 7.43 (t, J= 7.6 Hz, 1H, ArH), 7.62 (d, J= 7.6 Hz, 1H, ArH), 7.72 (t, J= 7.6 Hz, 1H, ArH), 8.20 (d, J= 7.6 Hz, 1H, ArH). ^13^C NMR (100 MHz: CDCl_3_): δ 22.23, 119.99, 126.35, 126.45, 126.87, 134.32, 146.90, 155.48, 161.56.

**General procedure for the preparation of (E)-3-(substituted benzylidene-amino)-2-methylquinazolin-4(3H)-one 14.** To a solution of compound **13** (0.25 g, 1.42 mmol), the appropriate aldehyde (1.42 mmol) in methanol (10 ml) was added 2 drops of anhydrous acetic acid. The reaction mixture was heated under reflux for 3 hours. The reaction mixture was left to cool to room temperature and the resulted solid was filtered off and recrystallized from ethanol.

**(E)-3-(benzylideneamino)-2-meth-quin-azolin-4(3H)-one 14a.** Compound **14a** was collected as white crystals: yield (91%), mp 186-187°C, [lit.^36^ 184-185°C]. IR (KBr): 1670 (C=O, amide) cm^-1^.^1^H NMR (400 MHz: DMSO-d6): δ 2.51 (s, 3H, CH_3_) , 7.52-7.68 (m, 5H, Ar-H ) 7.84 ( t, J = 7.9 HZ , 1H, Ar-H) , 7.98 ( d, J = 7.9 HZ , 2H , Ar-H ) , 8.17 ( d , J = 7.9 Hz , 1H , Ar-H) , 8.99 (s, 1H CH=N). ^13^C NMR (100 MHz: DMSO-d_6_): δ 22.39, 121.47, 126.84, 127.12, 127.25, 129.26, 129. 64, 132.80, 133.25, 134.86, 146.78, 153.87, 157.89, 169.84.

**(E)-3-((4-hydroxybenzylidene)amino)-2-methylquinazolin-4(3H)-one 14b.** Compound **14b** was collected as yellow crystals: yield (67 %), mp 210-212°C [lit.^36^ 210-214°C]. IR (KBr): 3080 (BB) (OH), 1680 (C=O, amide) cm^-1^. ^1^H NMR (DMSO-d_6_-400 MHz): δ 2.49 (s, 3H, CH_3_), 6.95 (d, J= 7.6 Hz, 2H, ArH), 7.51 (t, J= 7.6 Hz, 1H, Ar-H), 7.65 (d, J= 7.6 Hz, 1H, Ar-H), 7.80-7.84 (d & t (overlapped), 3H, Ar-H), 8.14 (d, J= 7.6 Hz, 1H, ArH), 8.72 (s, 1H, CH=N). ^13^C NMR (DMSO-d_6_-100 MHz): δ 22.62, 116.46, 121.46, 123.71, 127.16, 127.05, 131.48, 134.67, 146.81, 153.94, 157.99, 162.25, 169.81.

**(E)-3-((4-Methoxybenzylidene)amino)-2-methylquinazolin-4(3H)-one 14c.** Compound **14c** was collected as white crystals: yield (88%) , m,p.= 199-200°C [lit.^37^ 191°C]. IR (KBr): 1676 (C=O, amide) cm^-1^. ^1^H NMR (DMSO-d_6_-400 MHz): δ 2.51 (s, 3H, CH_3_), 3.87 (s, , 3H, OCH_3_), 7.14(d, J= 7.7 Hz, 2H, Ar-H), 7.53 (t, J= 7.20 Hz, 1H, Ar-H), 7.66 (d ,J=8Hz , 1H, Ar-H), 7.83 (t, J= 7.6 Hz, 1H, Ar-H), 7.92 (d, J= 8 Hz, 2H, Ar-H) , 8.15 (d, J=8 Hz , 1H , Ar-H), 8.83( s,1H,CH=N). ^13^C NMR (DMSO-d_6_, 100 MHz) : δ 22.64, 56.03 , 115.11, 121.45, 125.27, 126.75, 127.04, 127.19, 131.24 , 134.75 , 146.74, 153.87, 157.88 , 163.33 ,169.48.

**(E)-3-((4-hydroxy-3-methoxybenzyl-idene)amino)-2-methylquinazolin-4(3H)-one 14d.** Compound **14d** was collected as white crystals: yield (86 %); mp 123-125 °C, [lit.^38^ 122-124°C]. IR (KBr): 3010 (BB) (OH), 1680 (C=O, amide) cm^-1^. ^1^H NMR (400 MHz: DMSO-d_6_): δ 2.51 (s, 3H, CH_3_), 3.87 (s, 3H, OCH_3_), 6.95 (d, 1H, J= 8 Hz, ArH), 7.36 (d, 1H, J= 8 Hz, ArH), 7.52 (t, 1H, J= 8 Hz, ArH), 7.56 (s, 1H, ArH), 7.66 (d, 1H, J= 8 Hz, ArH), 7.82 (t, 1H, J= 8 Hz, ArH), 8.14 (d, 1H, J= 8 Hz, ArH), 8.71 (s, 1H, CH=N). ^13^C NMR (100 MHz: DMSO-d_6_): δ 22.67, 56.10, 110.98, 116.05, 121.46, 124.02, 125.06, 126.69, 127.01, 127.19, 134.68, 146.83, 148.60, 151.91, 153.94, 157.87, 169.97. MS-MALDI: m/e 110.11 [M+1].

Table S1. Crystal data and structure refinement for **4c**.

| Identification code | SA2AAT1RT | |
| --- | --- | --- |
| Chemical formula | C17H17ClN4O3 | |
| Formula weight | 360.79 g/mol | |
| Temperature | 293(2) K | |
| Wavelength | 0.71073 Å | |
| Crystal habit | clear colourless block | |
| Crystal system | triclinic | |
| Space group | P -1 | |
| Unit cell dimensions | a = 9.354(4) Å | α = 65.465(8)° |
|  | b = 9.403(4) Å | β = 79.165(8)° |
|  | c = 11.133(4) Å | γ = 75.063(10)° |
| Volume | 857.0(6) Å^3^ | |
| Z | 2 | |
| Density (calculated) | 1.398 g/cm^3^ | |
| Absorption coefficient | 0.247 mm^-1^ | |
| F(000) | 376 | |
| Theta range for data collection | 2.26 to 25.00° | |
| Index ranges | -11<=h<=11, -11<=k<=11, -11<=l<=13 | |
| Reflections collected | 17019 | |
| Independent reflections | 3020 [R(int) = 0.0388] | |
| Coverage of independent reflections | 99.9% | |
| Absorption correction | Multi-Scan | |
| Refinement method | Full-matrix least-squares on F^2^ | |
| Refinement program | SHELXL-2014/7 (Sheldrick, 2014) | |
| Function minimized | Σ w(F_o_^2^ - F_c_^2^)^2^ | |
| Data / restraints / parameters | 3020 / 1 / 240 | |
| Goodness-of-fit on F^2^ | 1.050 | |
| Final R indices | 2354 data; I>2σ(I) | R1 = 0.0389, wR2 = 0.0937 |
|  | all data | R1 = 0.0548, wR2 = 0.1036 |
| Weighting scheme | w=1/[σ^2^(F_o_^2^)+(0.0449P)^2^+0.3483P] where P=(F_o_^2^+2F_c_^2^)/3 | |
| Extinction coefficient | 0.0200(30) | |
| Largest diff. peak and hole | 0.207 and -0.251 eÅ^-3^ | |
| R.M.S. deviation from mean | 0.038 eÅ^-3^ | |


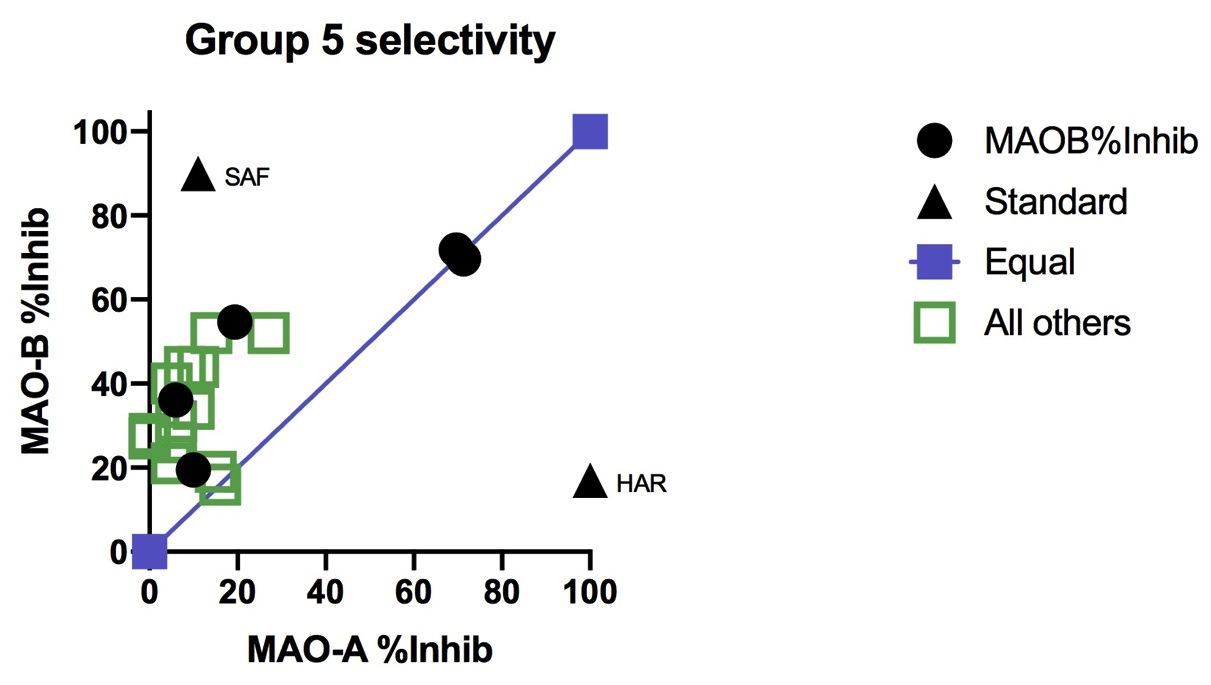


Series **5**

Figure S1. Selectivity for MAO –B over MAO-A. Series **5** compounds are shown as black circles, all other compounds as open squares. Triangles indicate the % inhibition by standard inhibitors, harmine for MAO –A and safinamide for MAO-B

**MAO-A Analysis** MAO-B Analysis


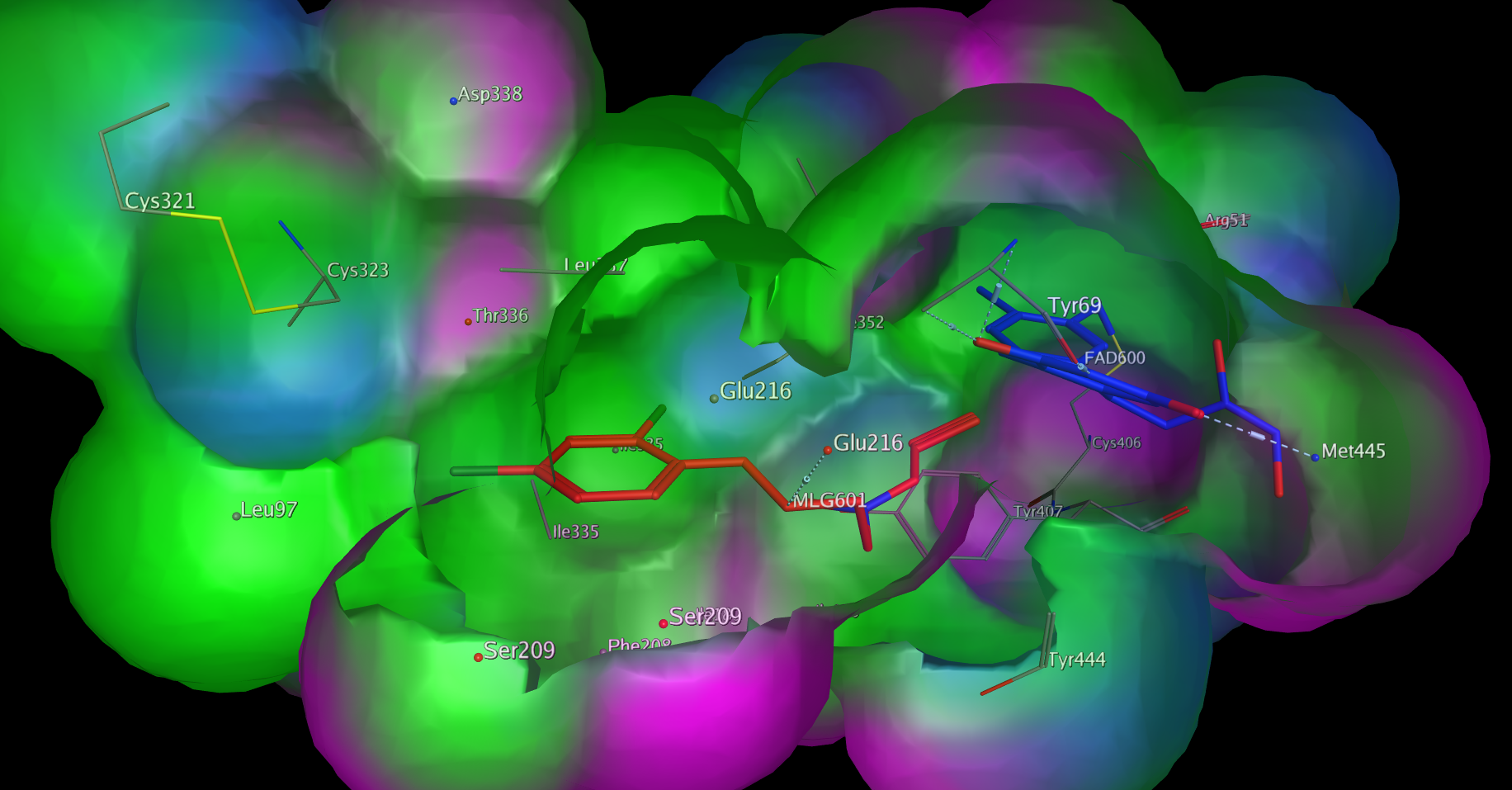

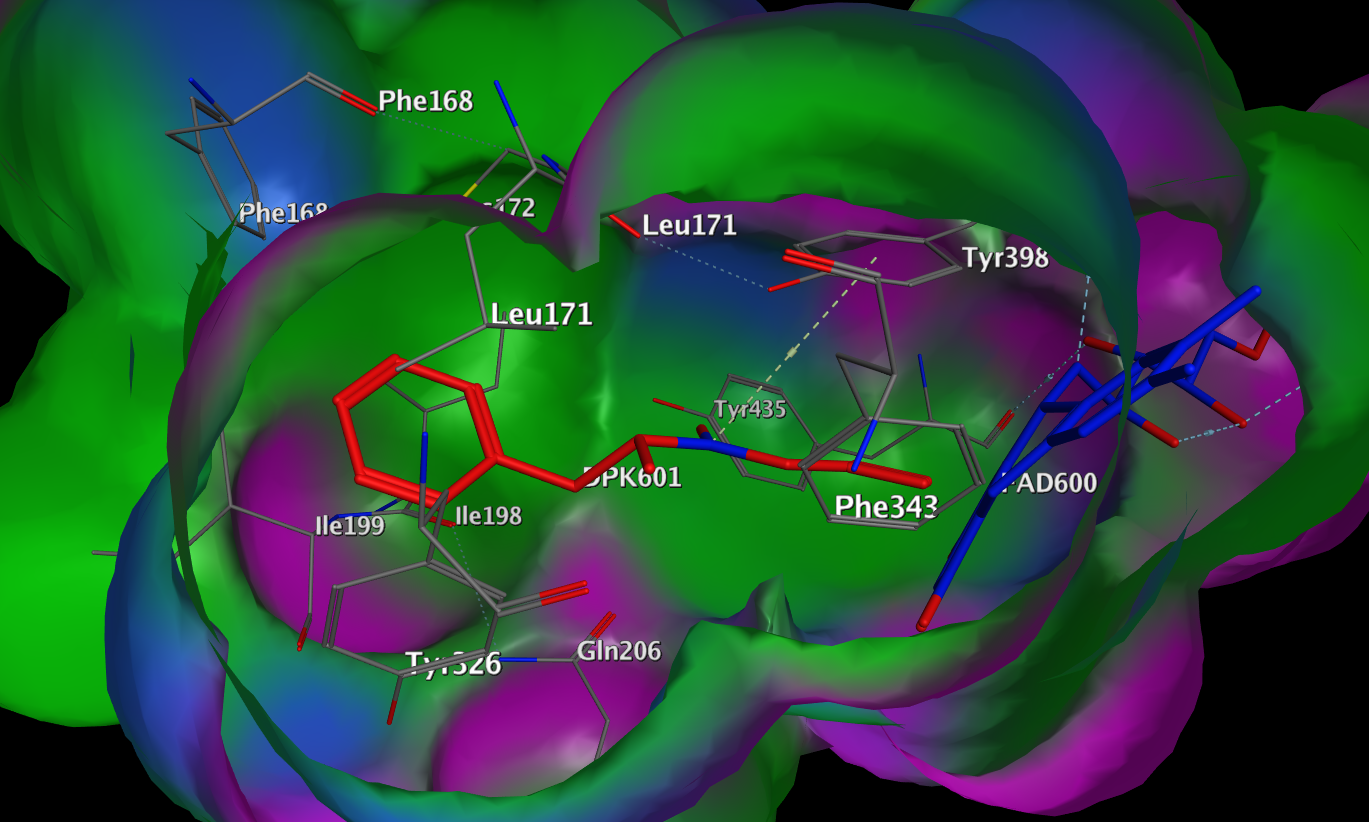


Clorgyline bound (2BXR) Deprenyl bound (2BYB)

Figure S2. Docking of ligands removed from the MAO crystal structures. Structures were prepared for docking as described in the Experimental Section. A. Clorgyline docked into MAO-A (2BXR); B. Deprenyl docked into MAO-B (2BYB).


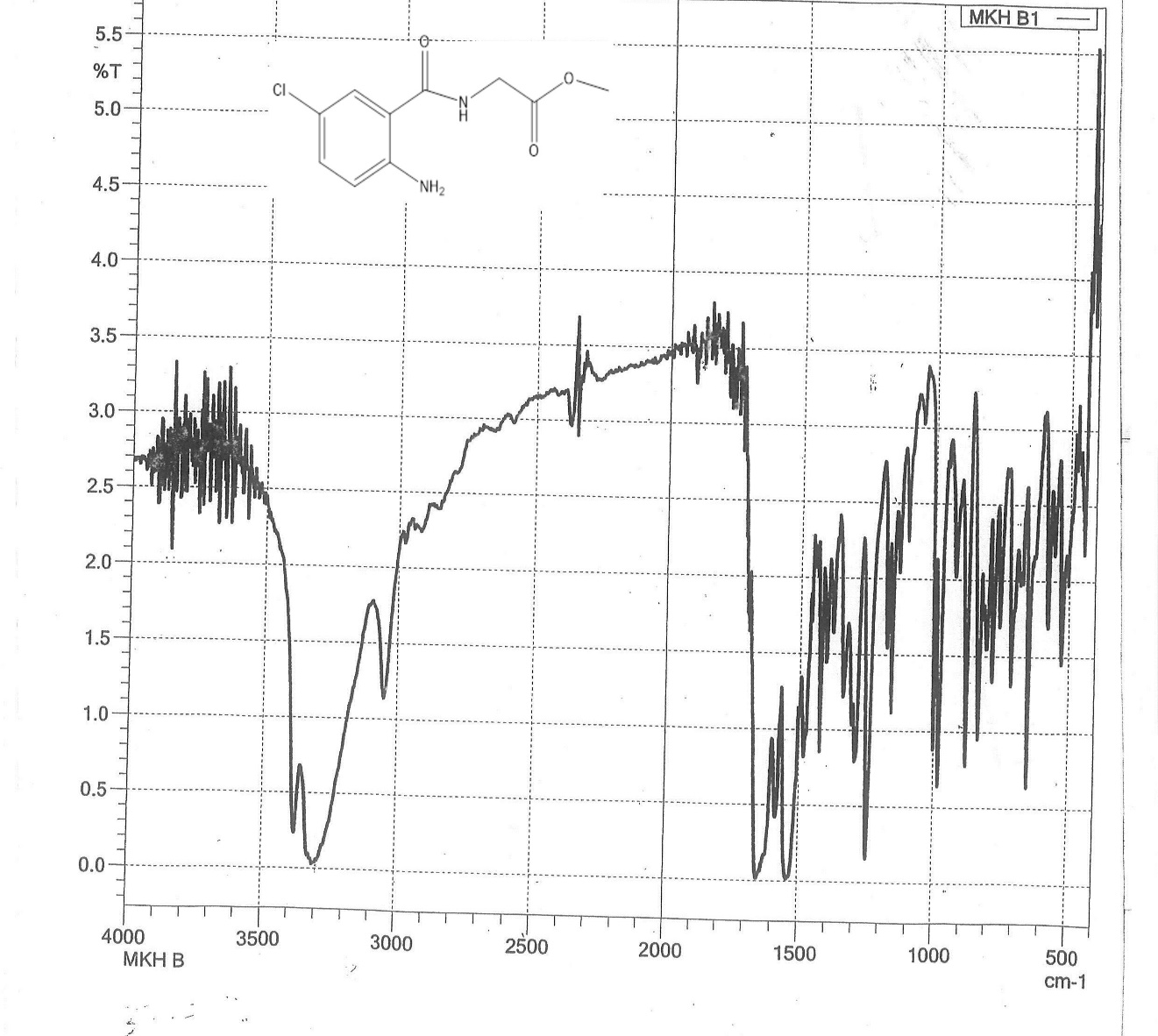


Figure S3. IR spectra of compound (2b)


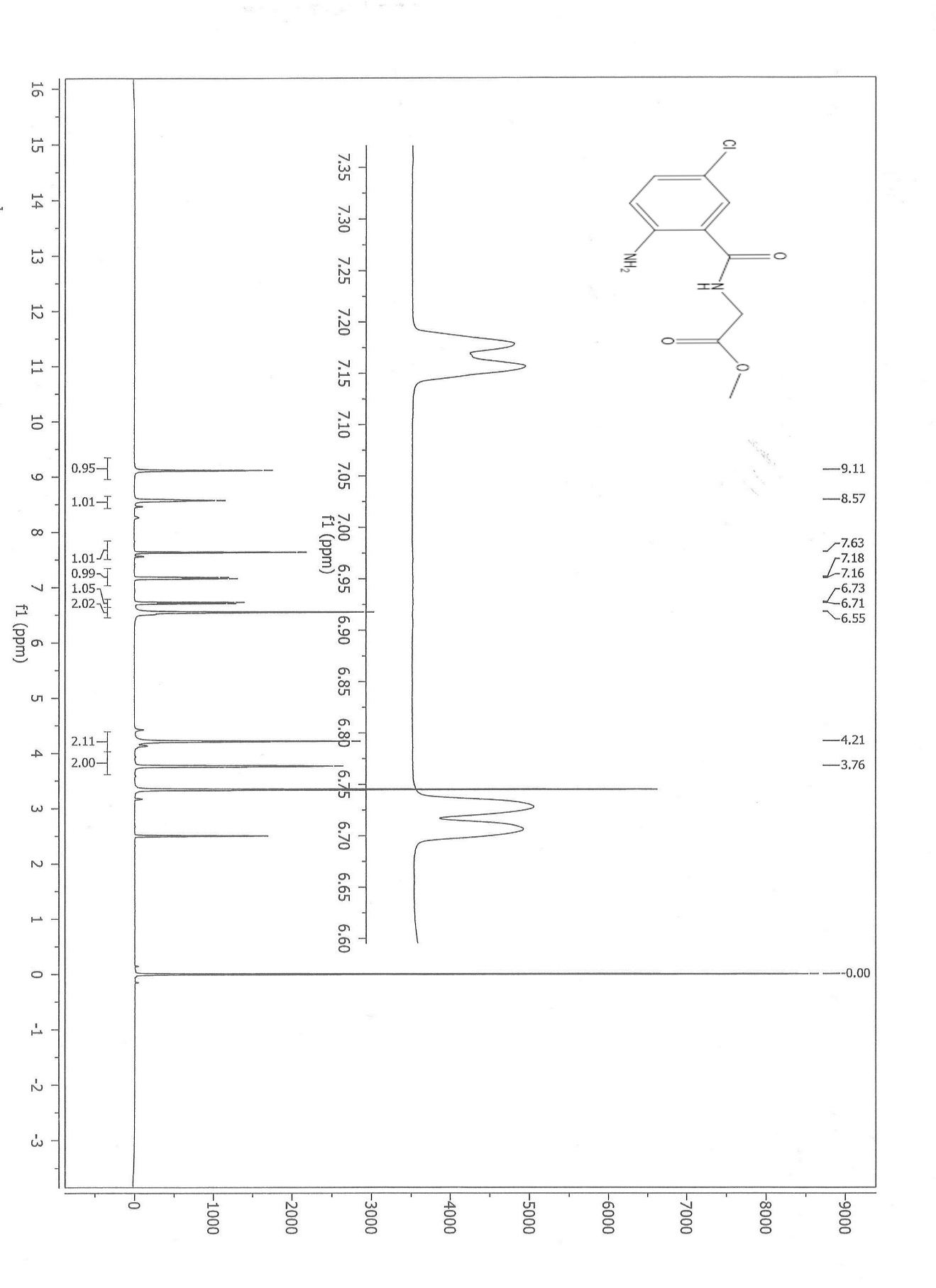


Figure S4. H^1^ spectra of compound (2b)


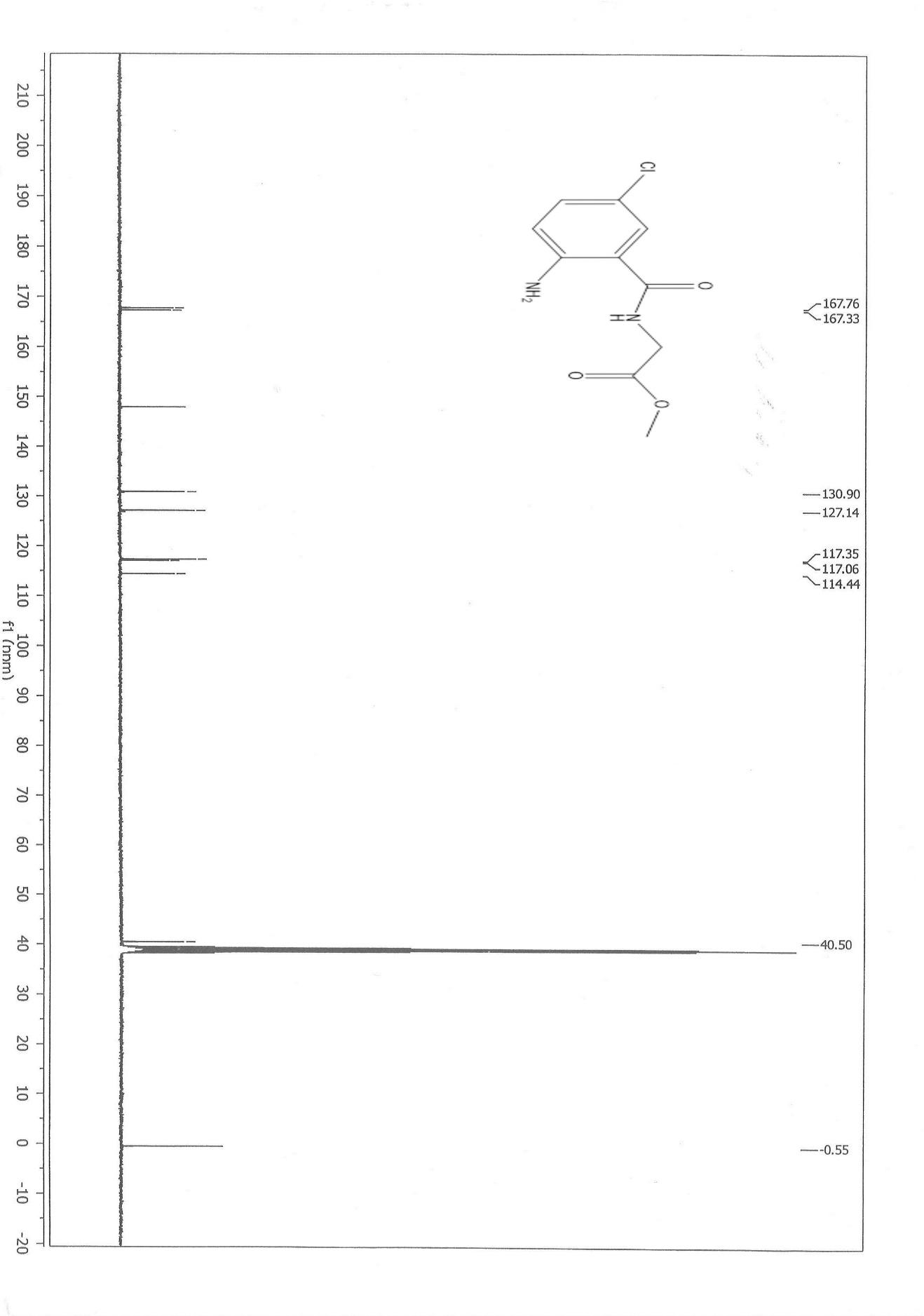


Figure S5. C^13^ spectra of compound (2b)


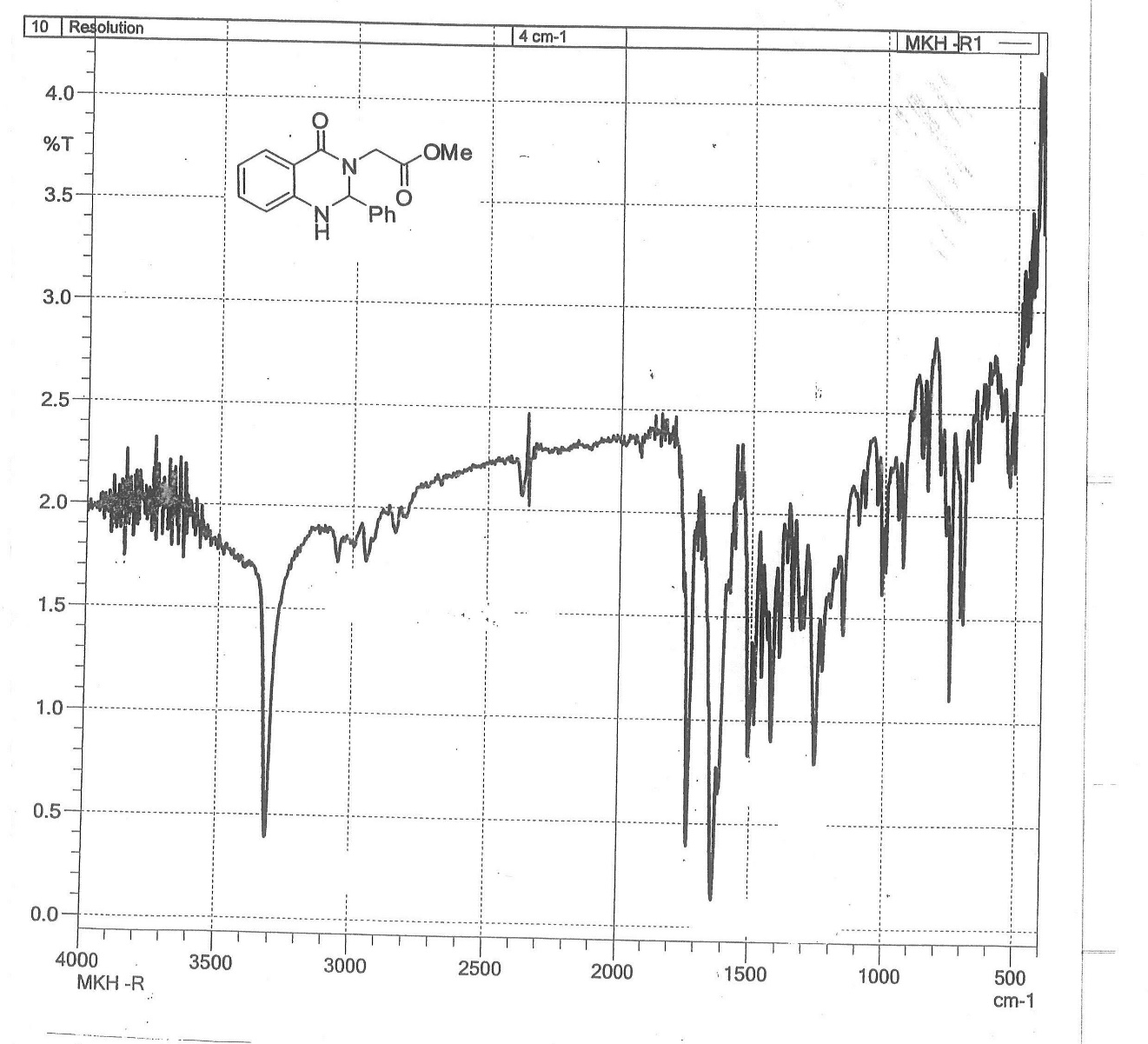


Figure S6. IR spectra of compound (3a)


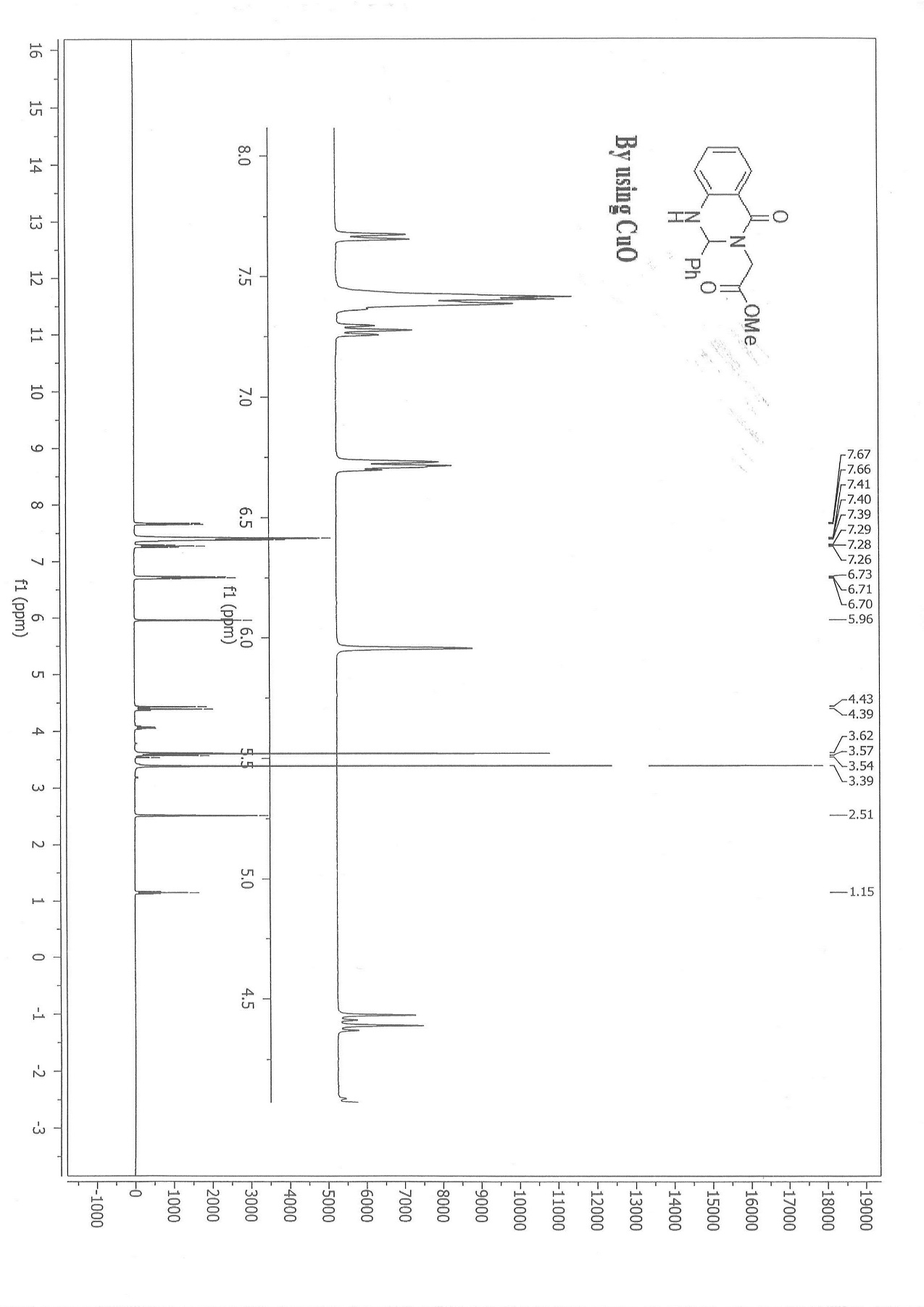


Figure S7. H^1^ spectra of compound (3a)


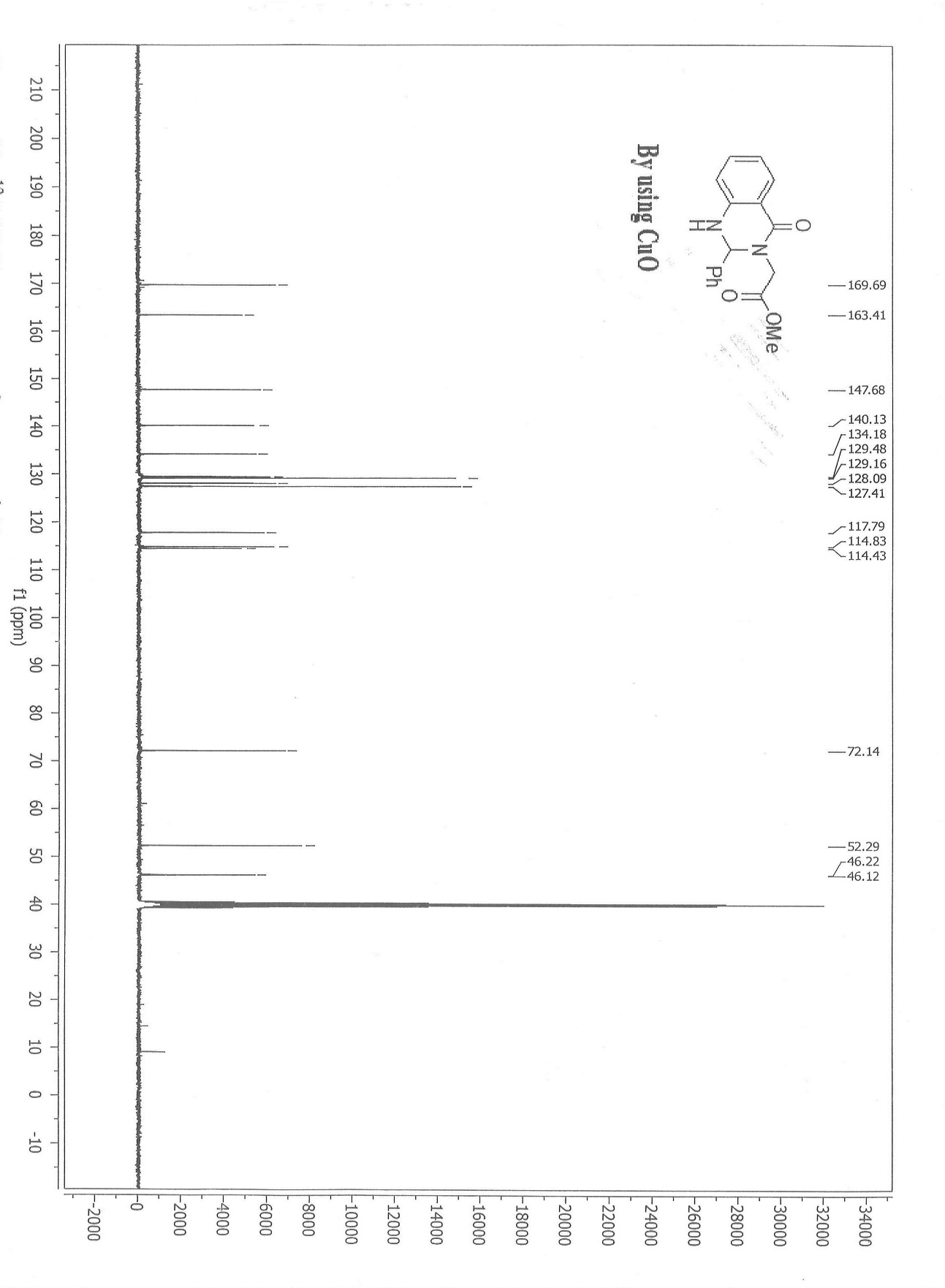


Figure S8. C^13^ spectra of compound (3a)


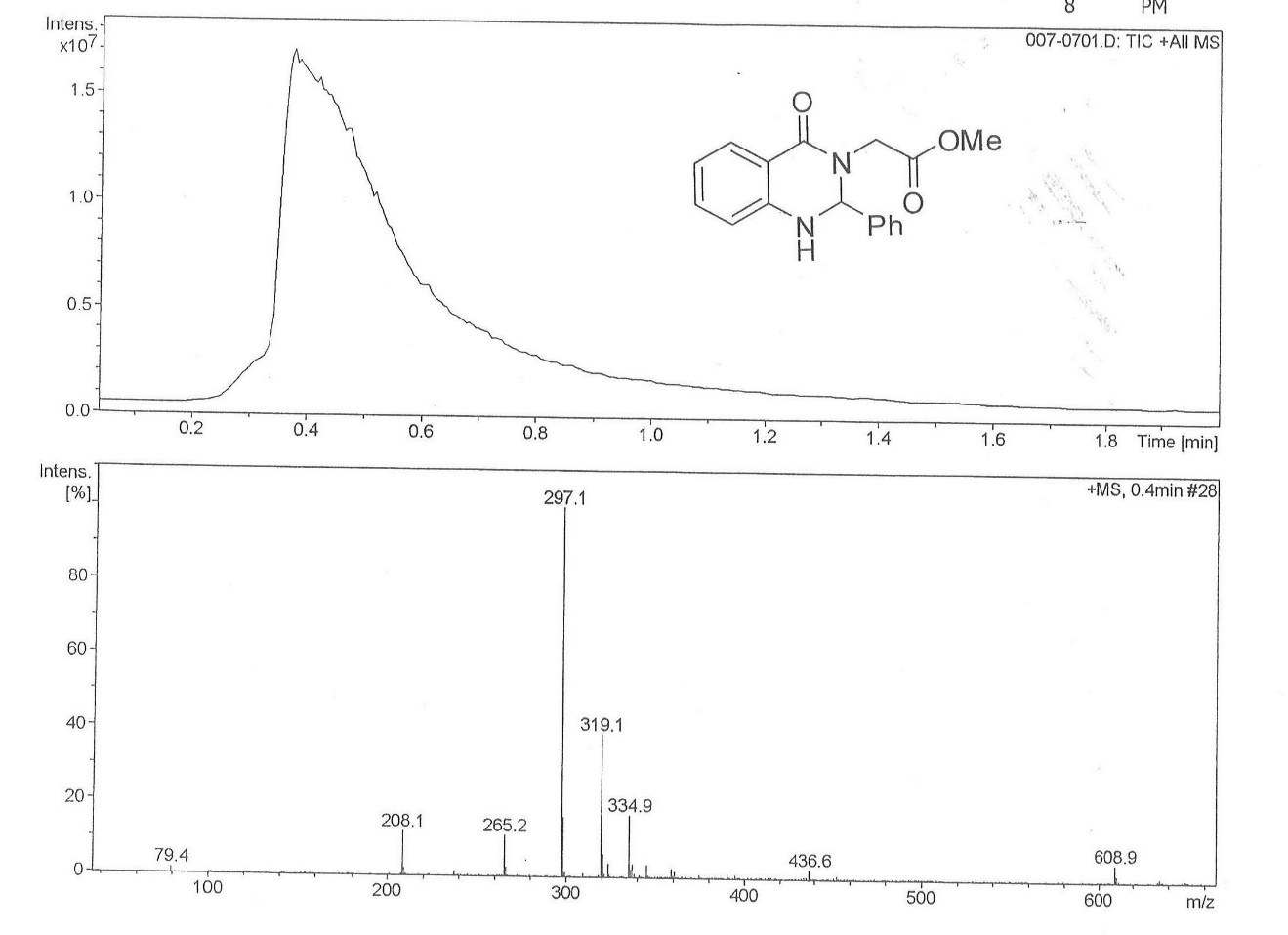


Figure S9. MS-ESI spectra of compound (3a)


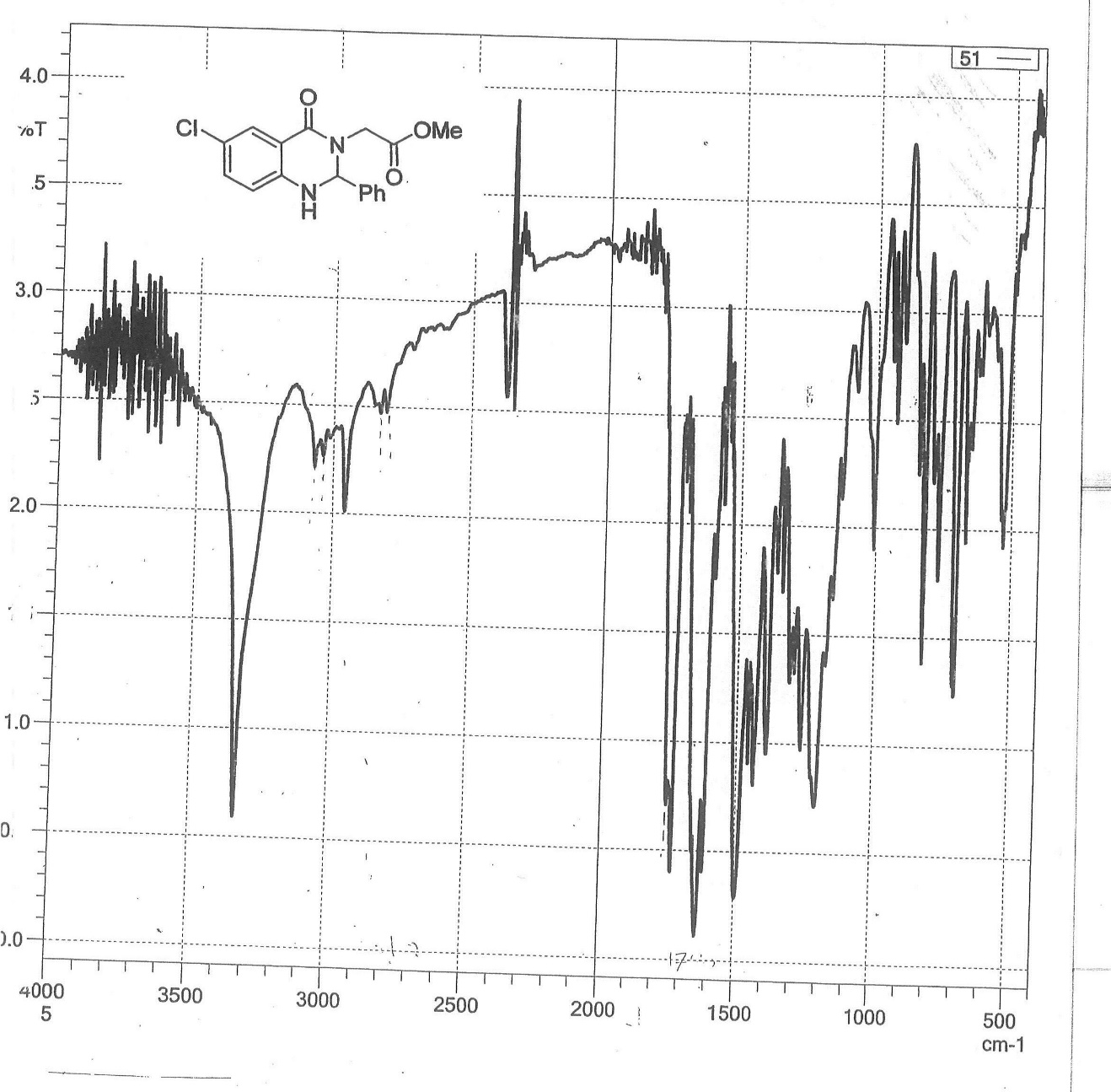


Figure S10. IR spectra of compound (3b)


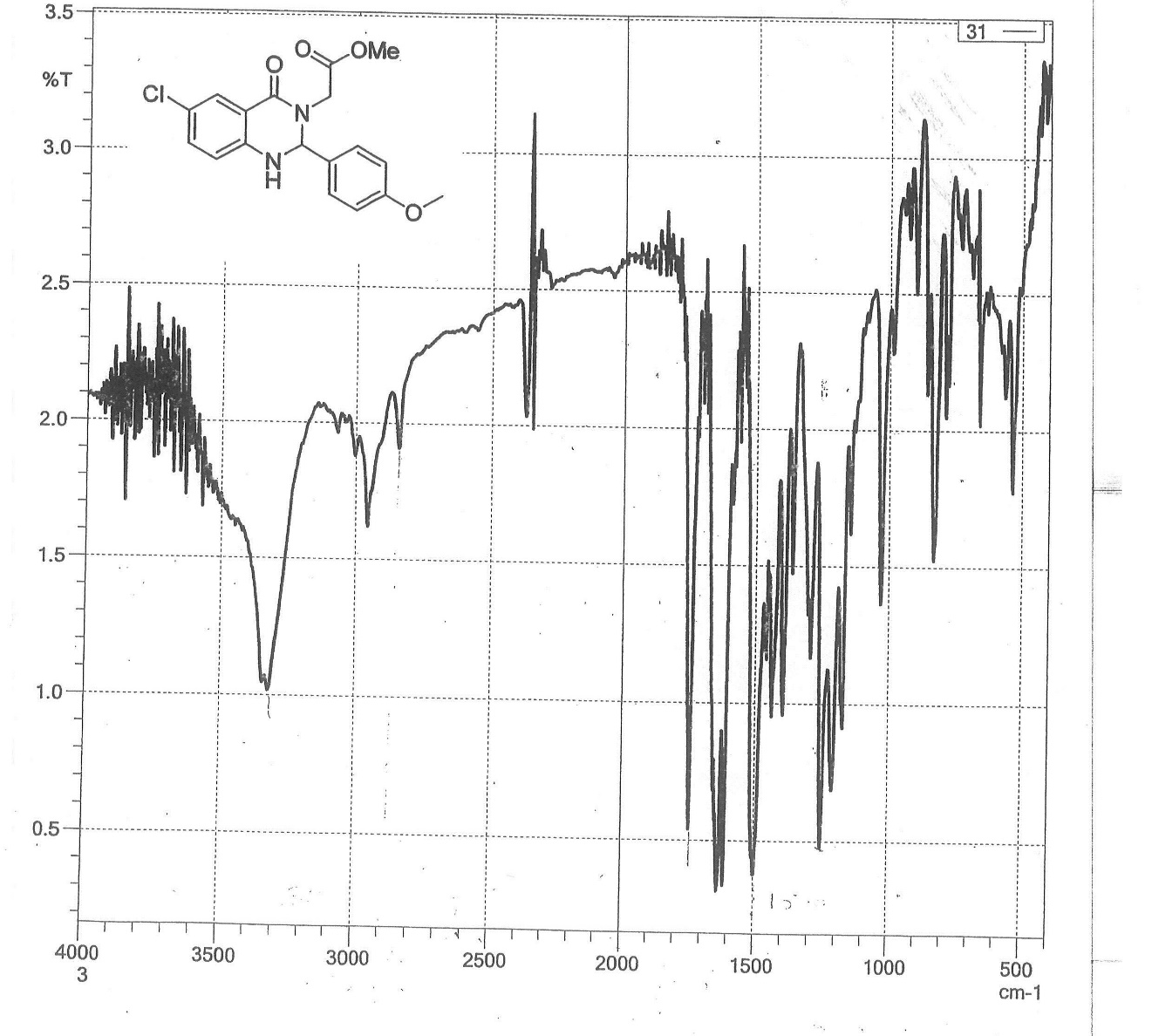


Figure S11. IR spectra of compound (3c)


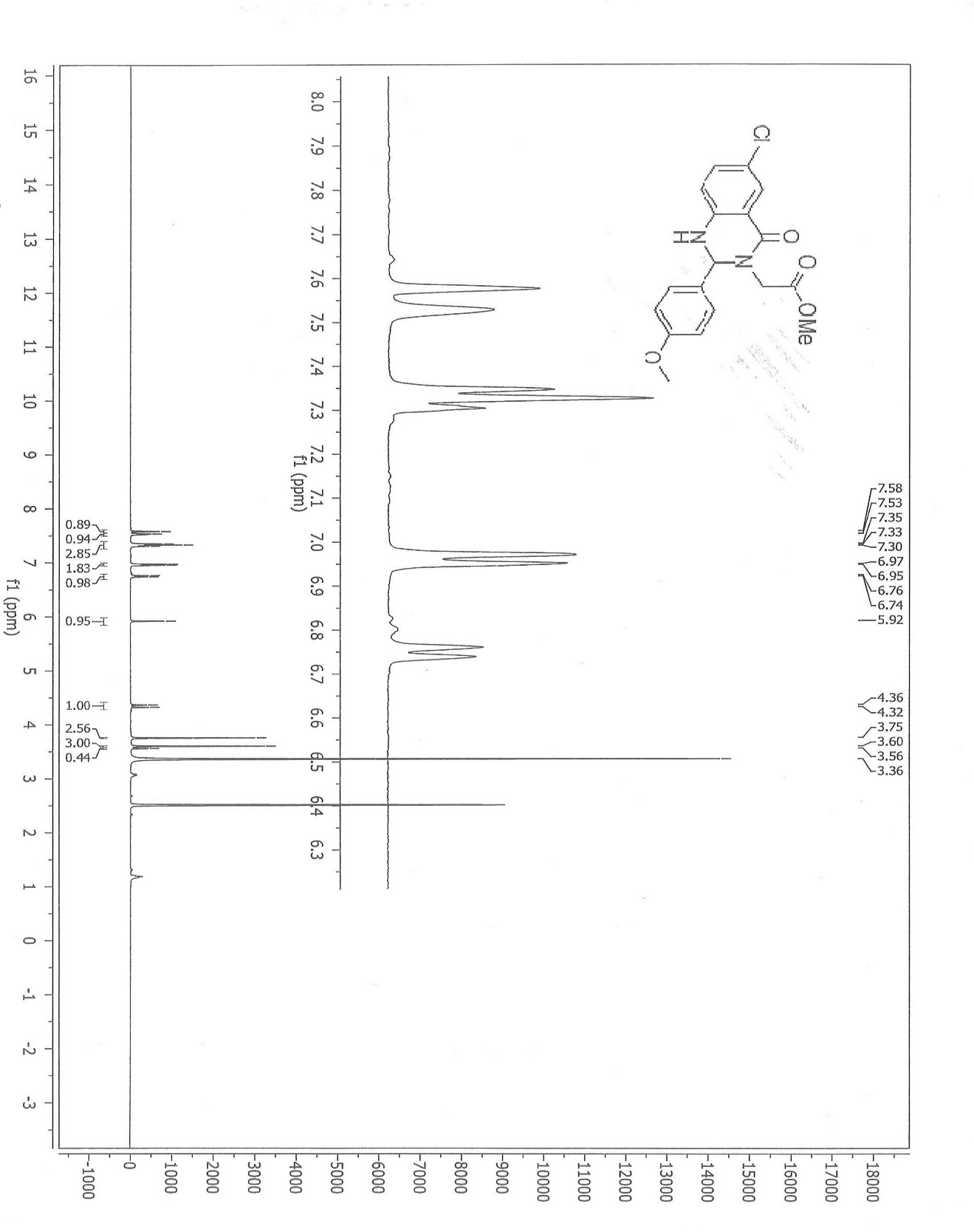


Figure S12. H^1^ spectra of compound (3c)


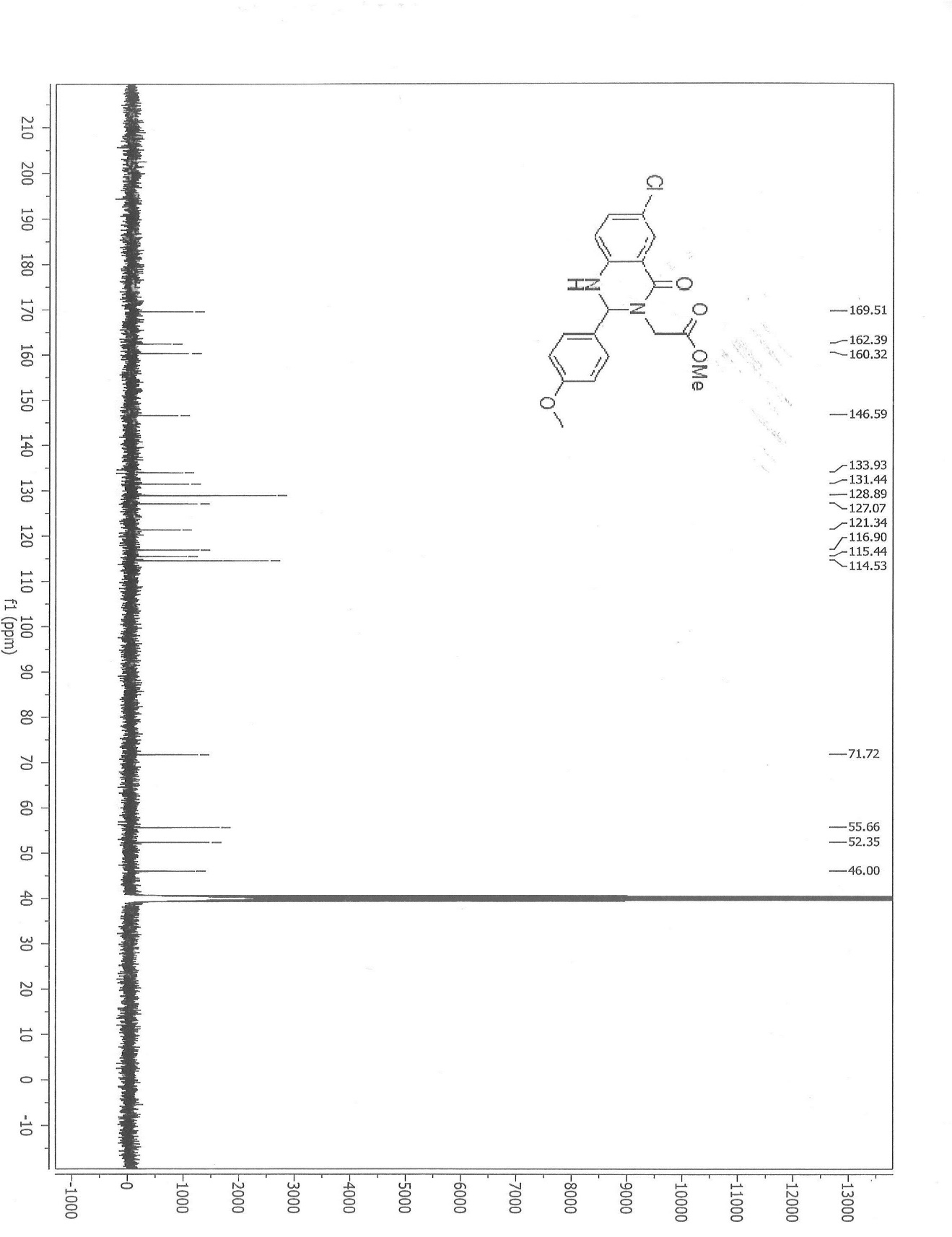


Figure S13. C^13^ spectra of compound (3c)


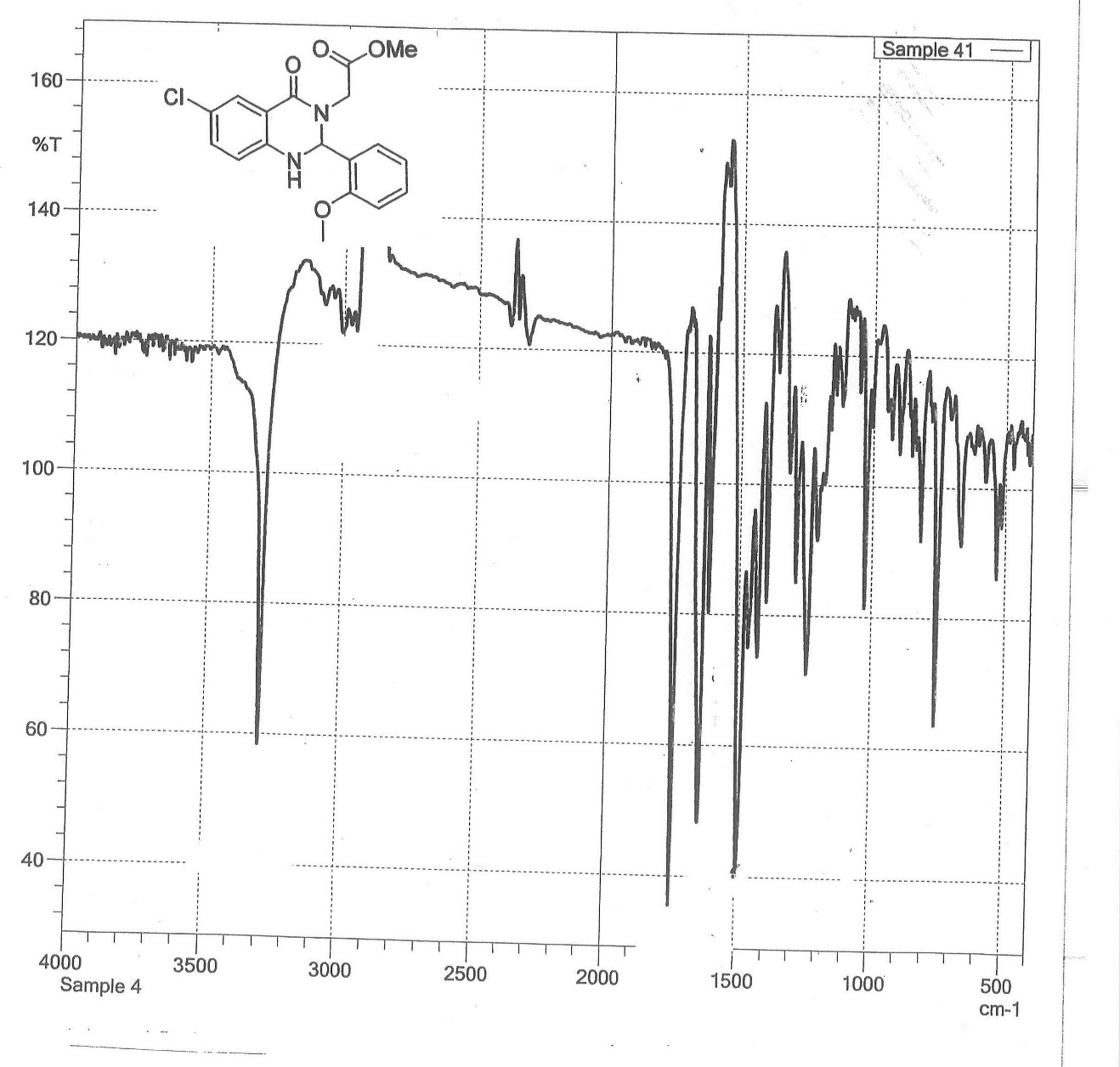


Figure S14. IR spectra of compound (3d)


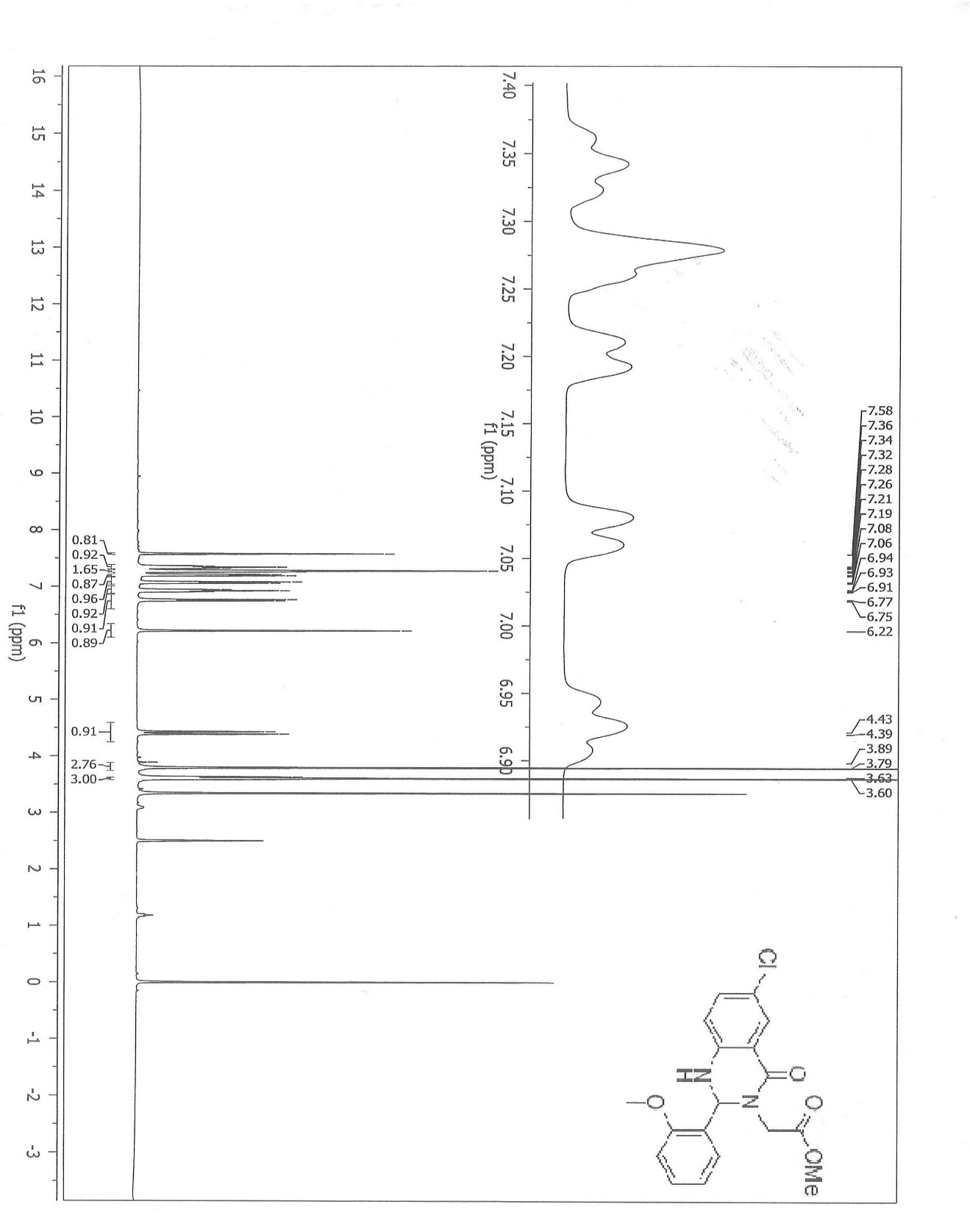


Figure S15. H^1^ spectra of compound (3d)


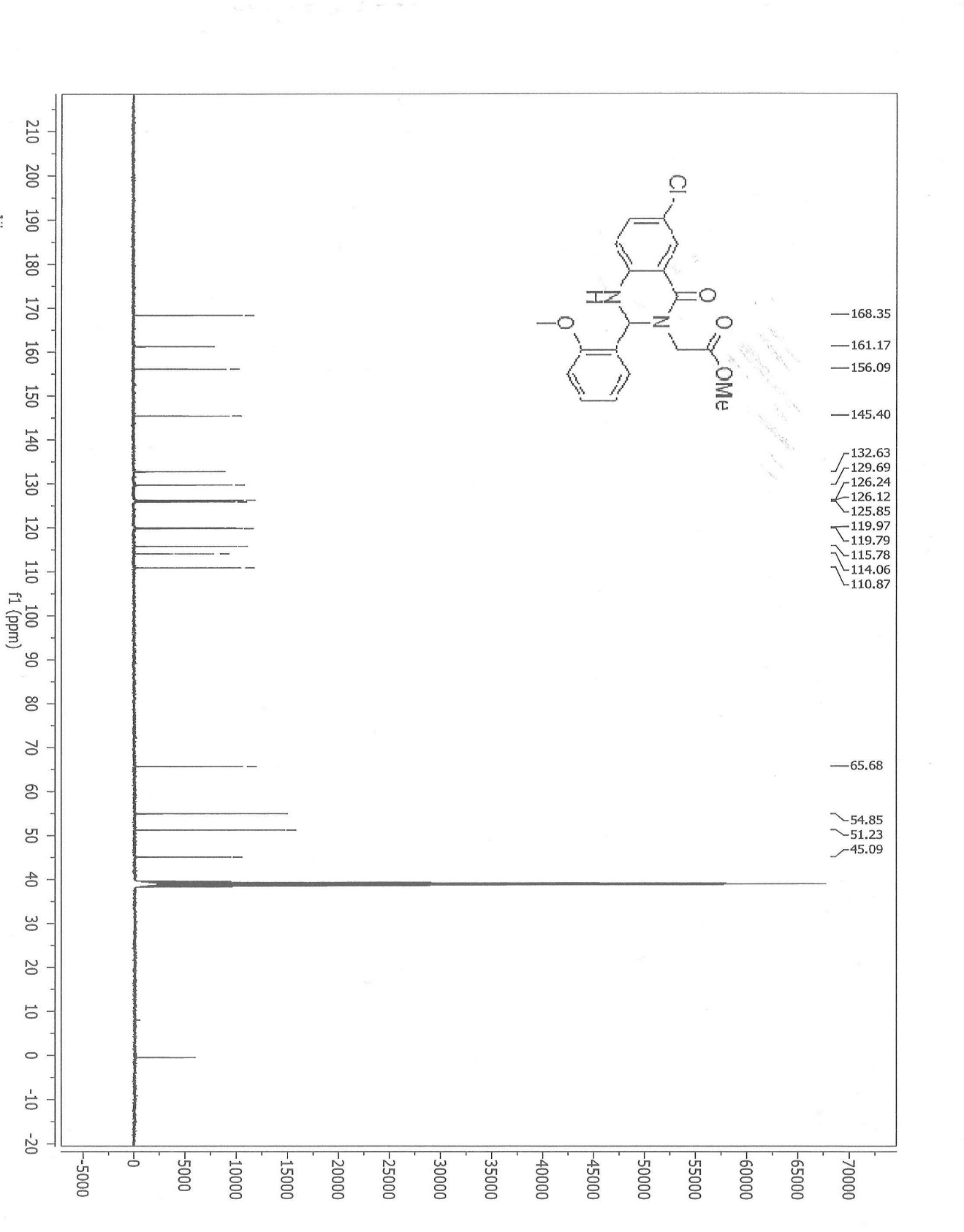


Figure S16. C^13^ spectra of compound (3d)


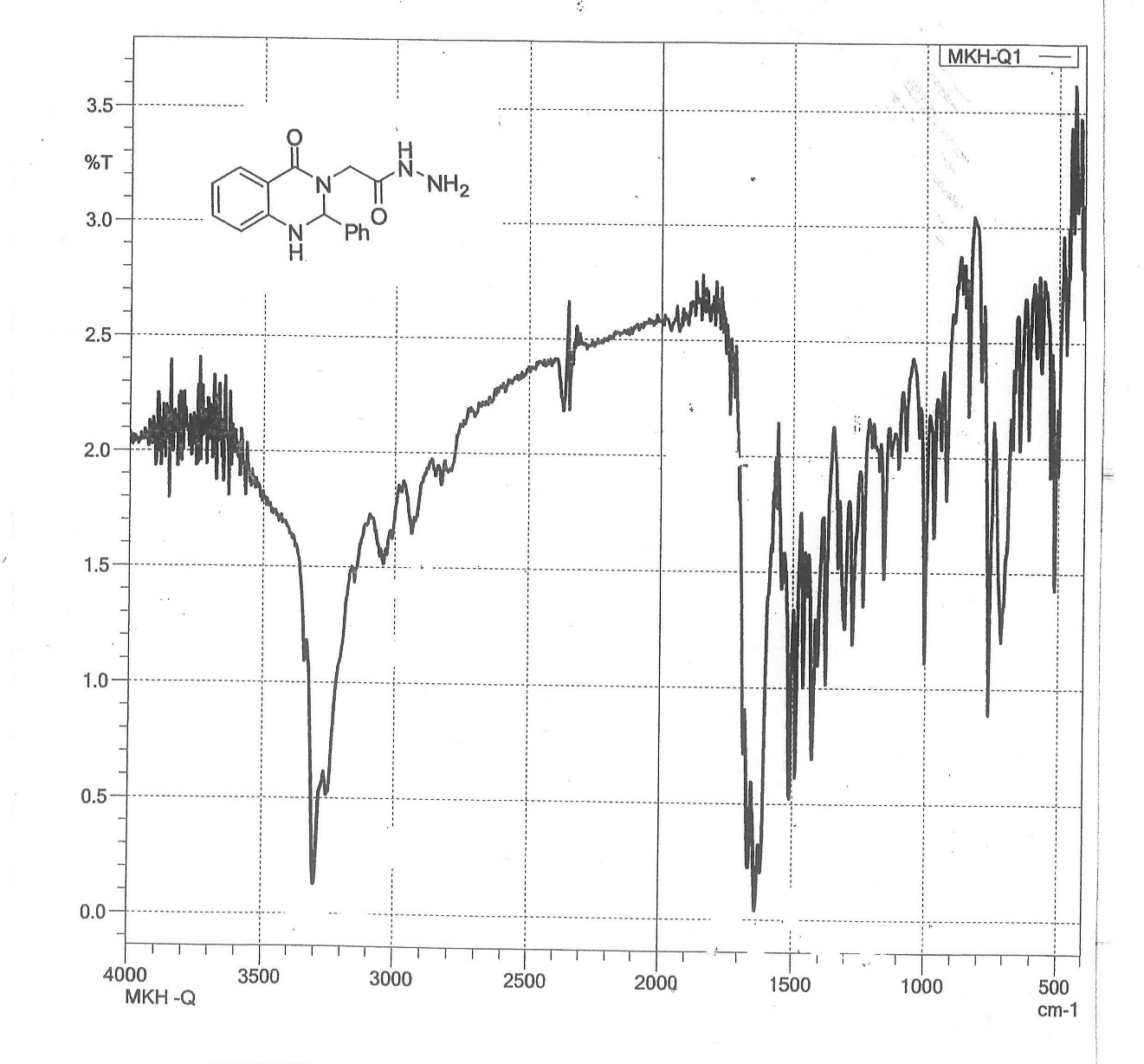


Figure S17. IR spectra of compound (4a)


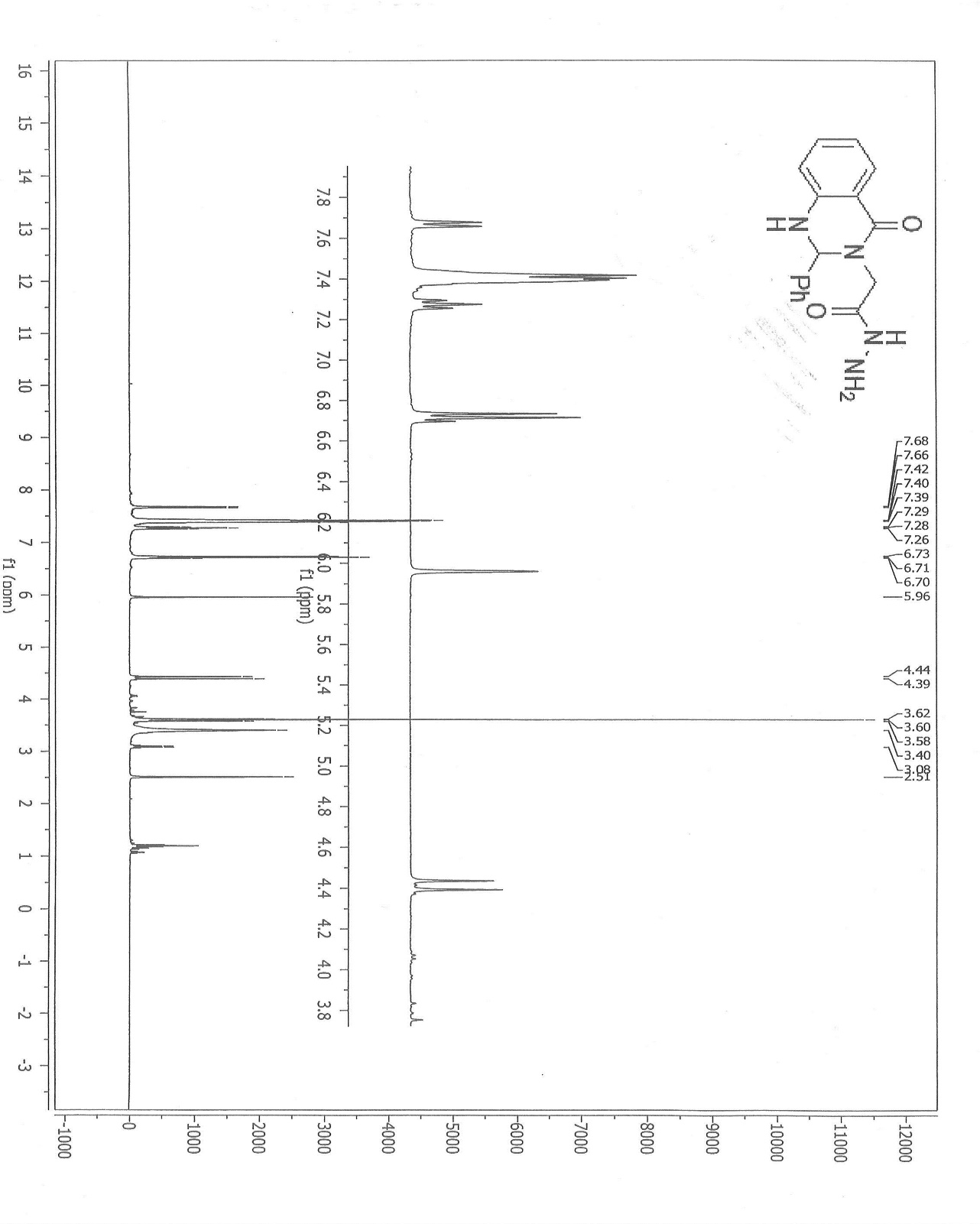


Figure S18. H^1^ spectra of compound (4a)


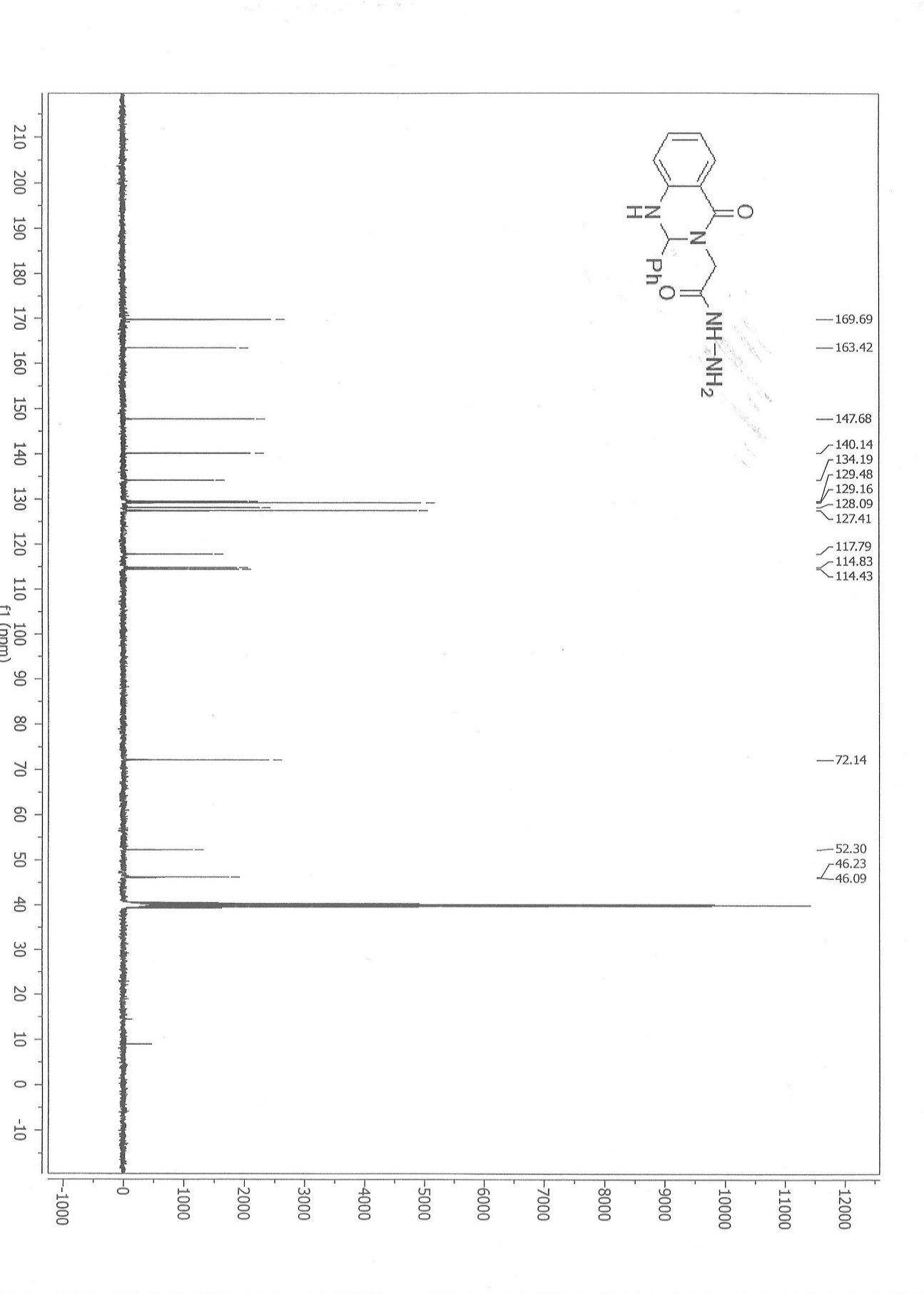


Figure S19. C^13^ spectra of compound (4a)


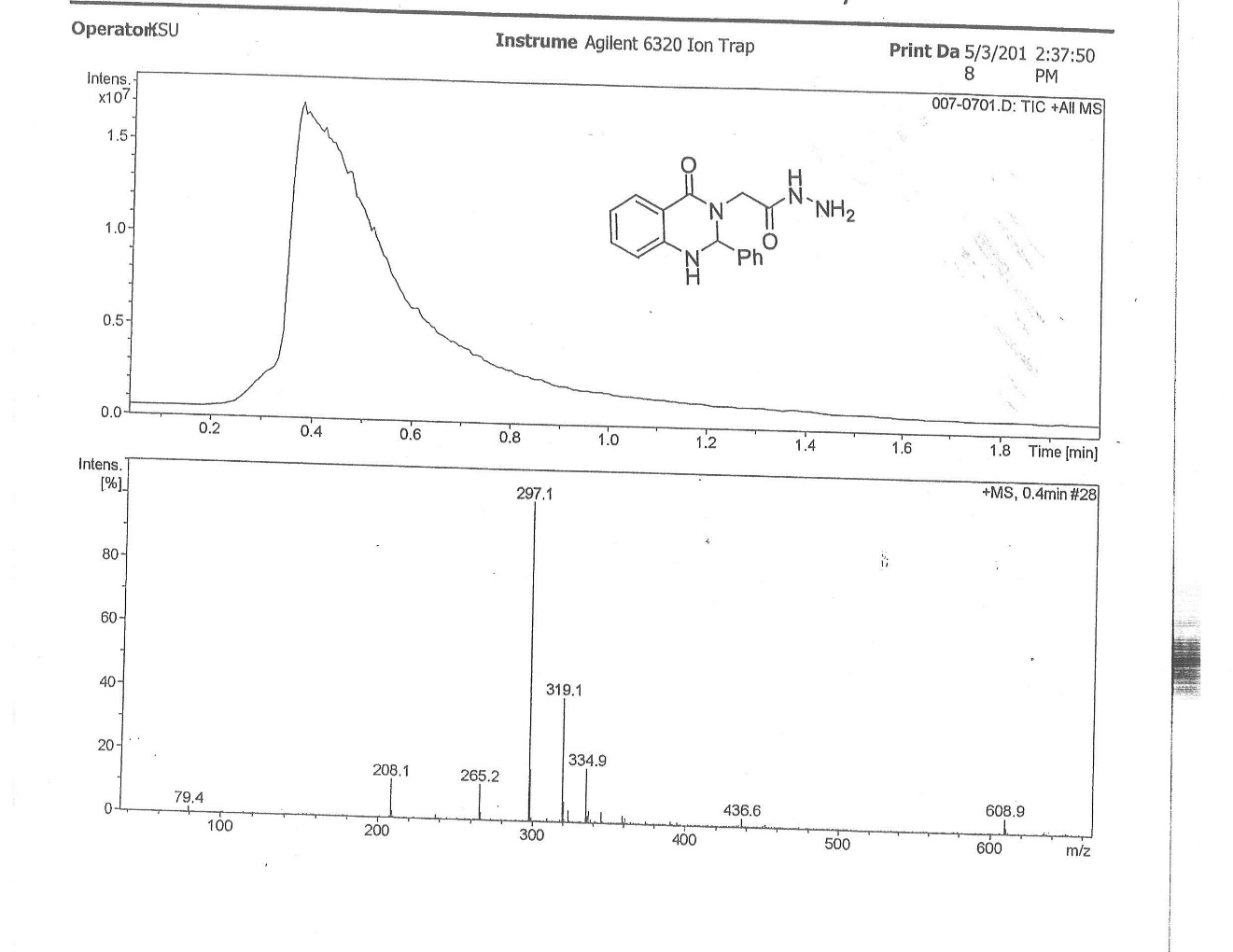


Figure S20. MS-ESI spectra of compound (4a)


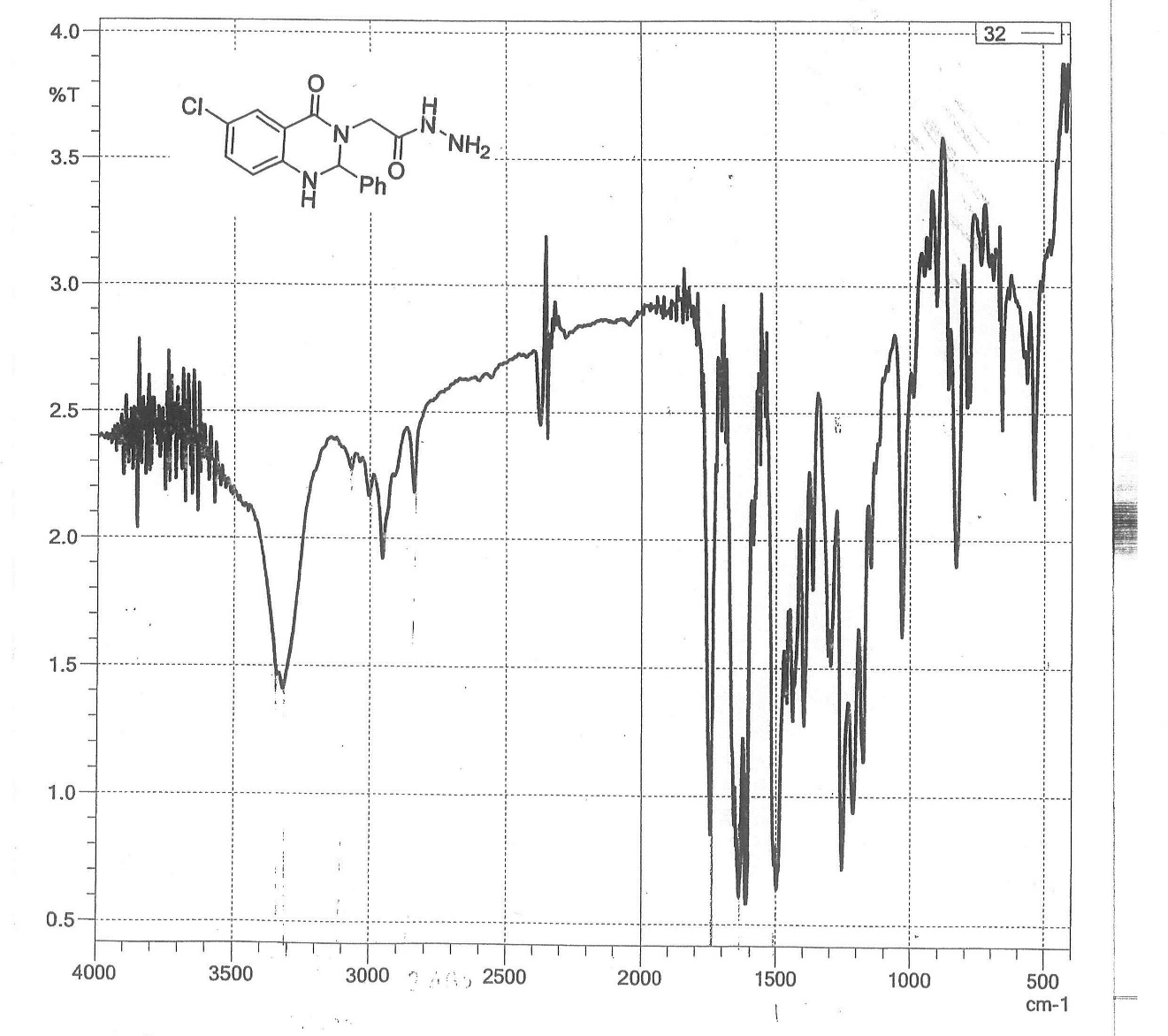


Figure S21. IR spectra of compound (4b)


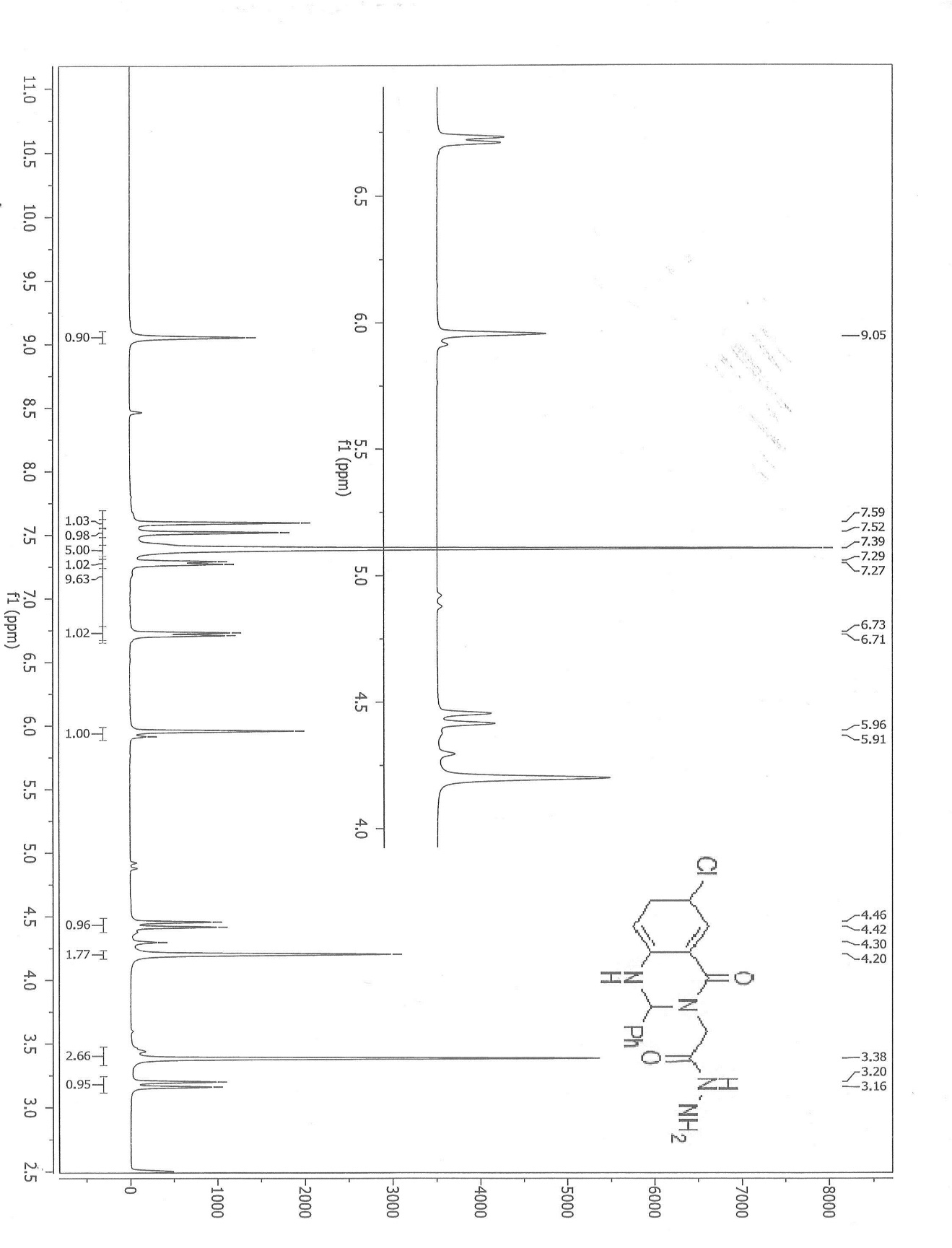


Figure S22. H^1^ spectra of compound (4b)


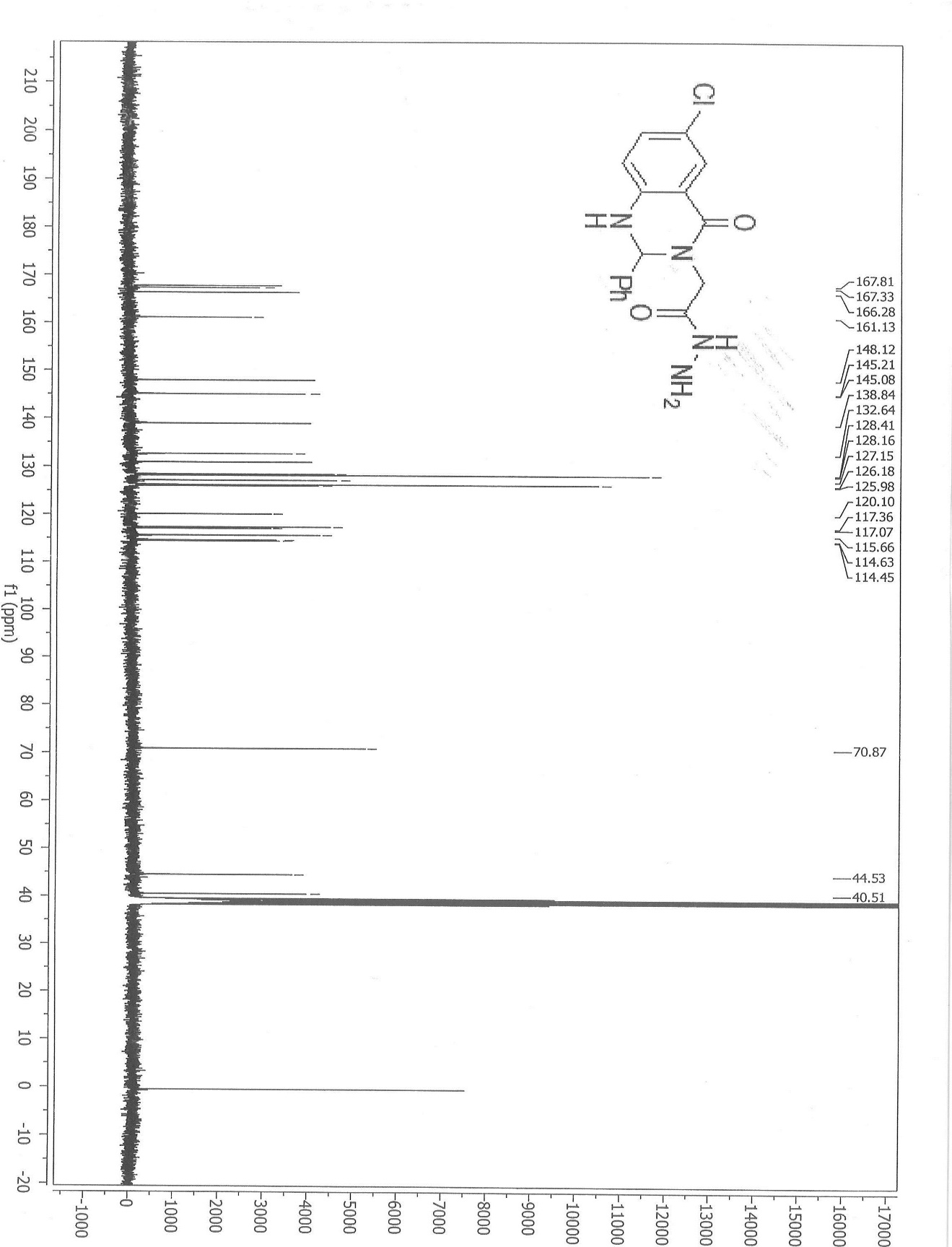


Figure S23. C^13^ spectra of compound (4b)


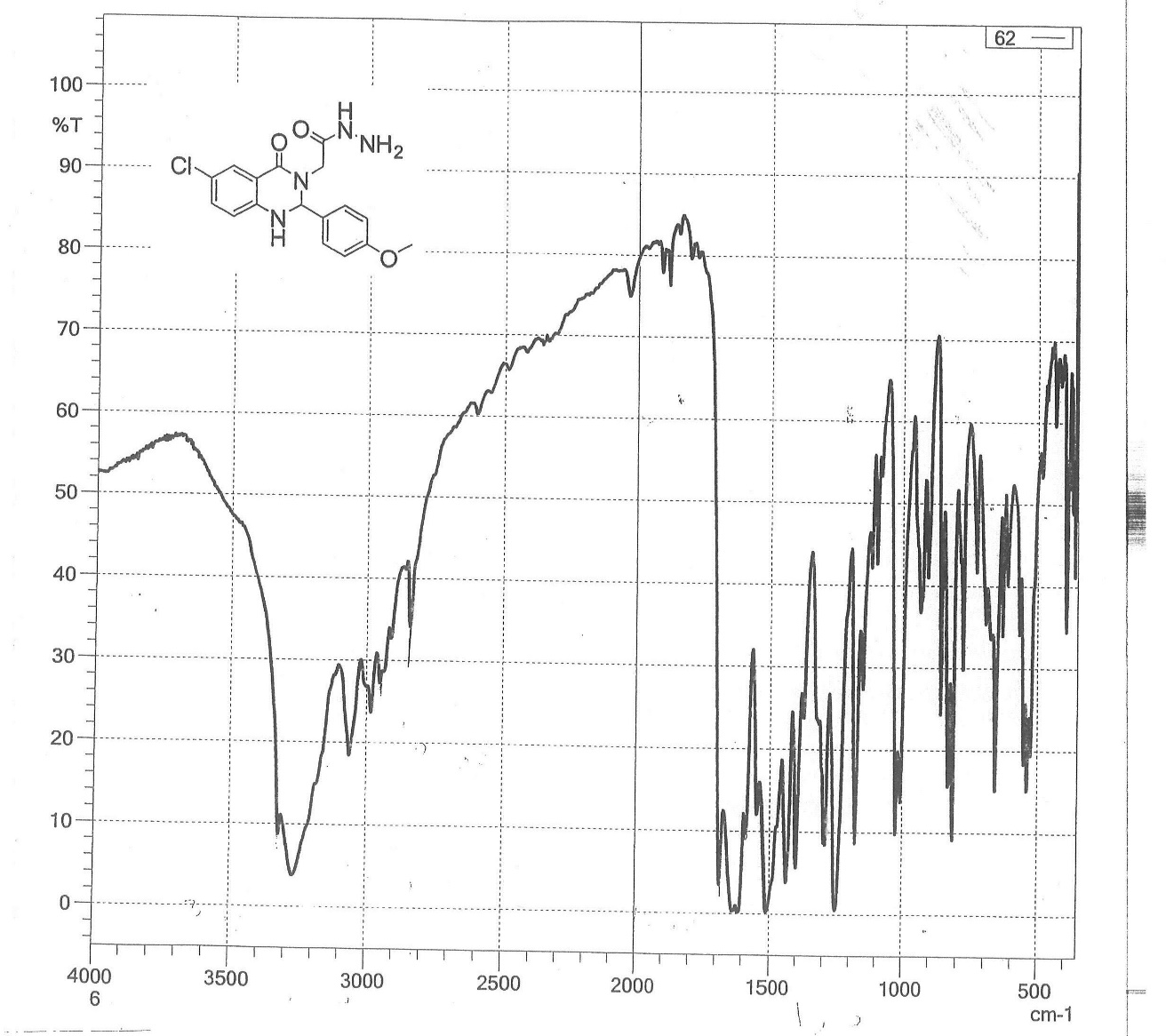


Figure S24. IR spectra of compound (4c)


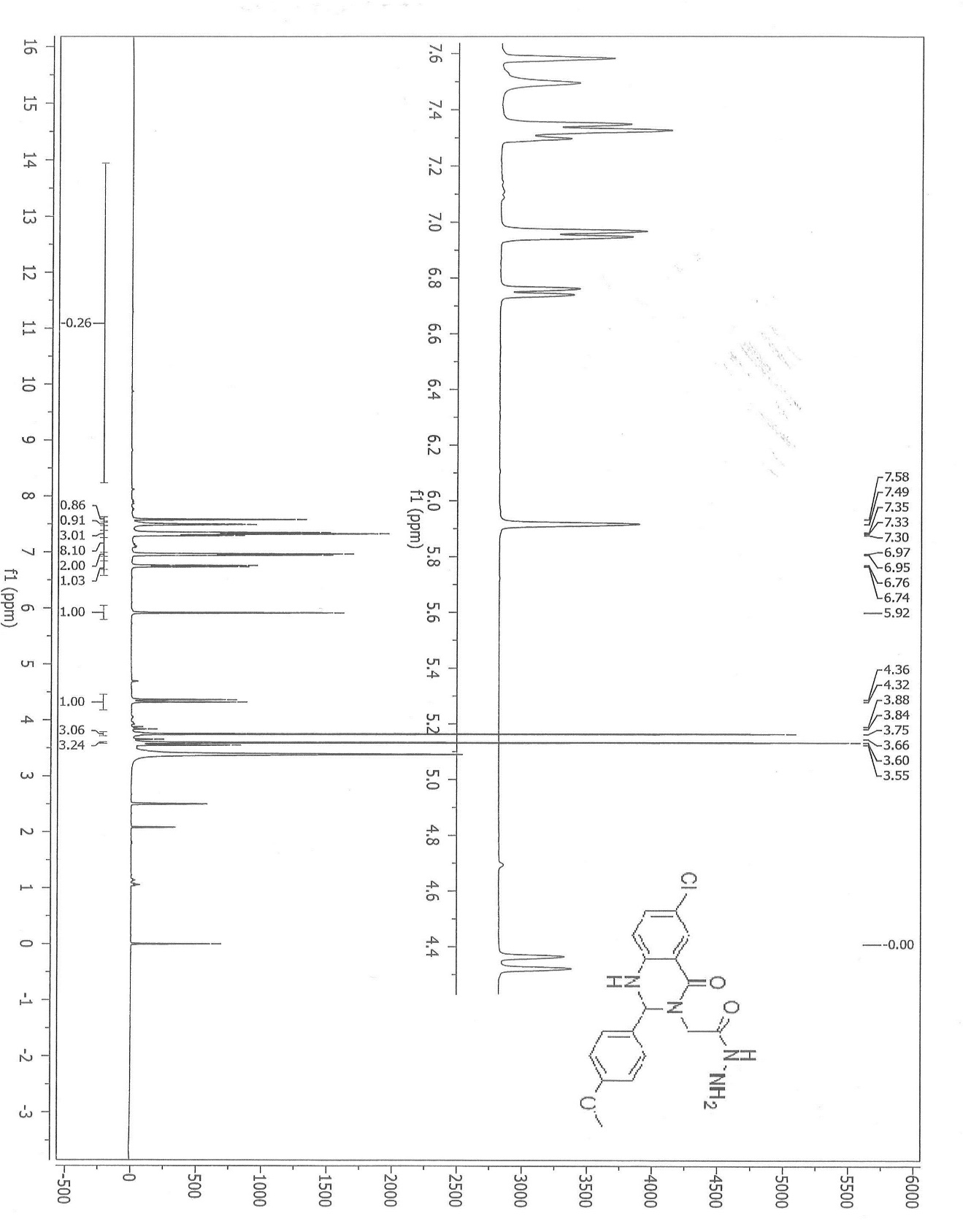


Figure S25. H^1^ spectra of compound (4c)


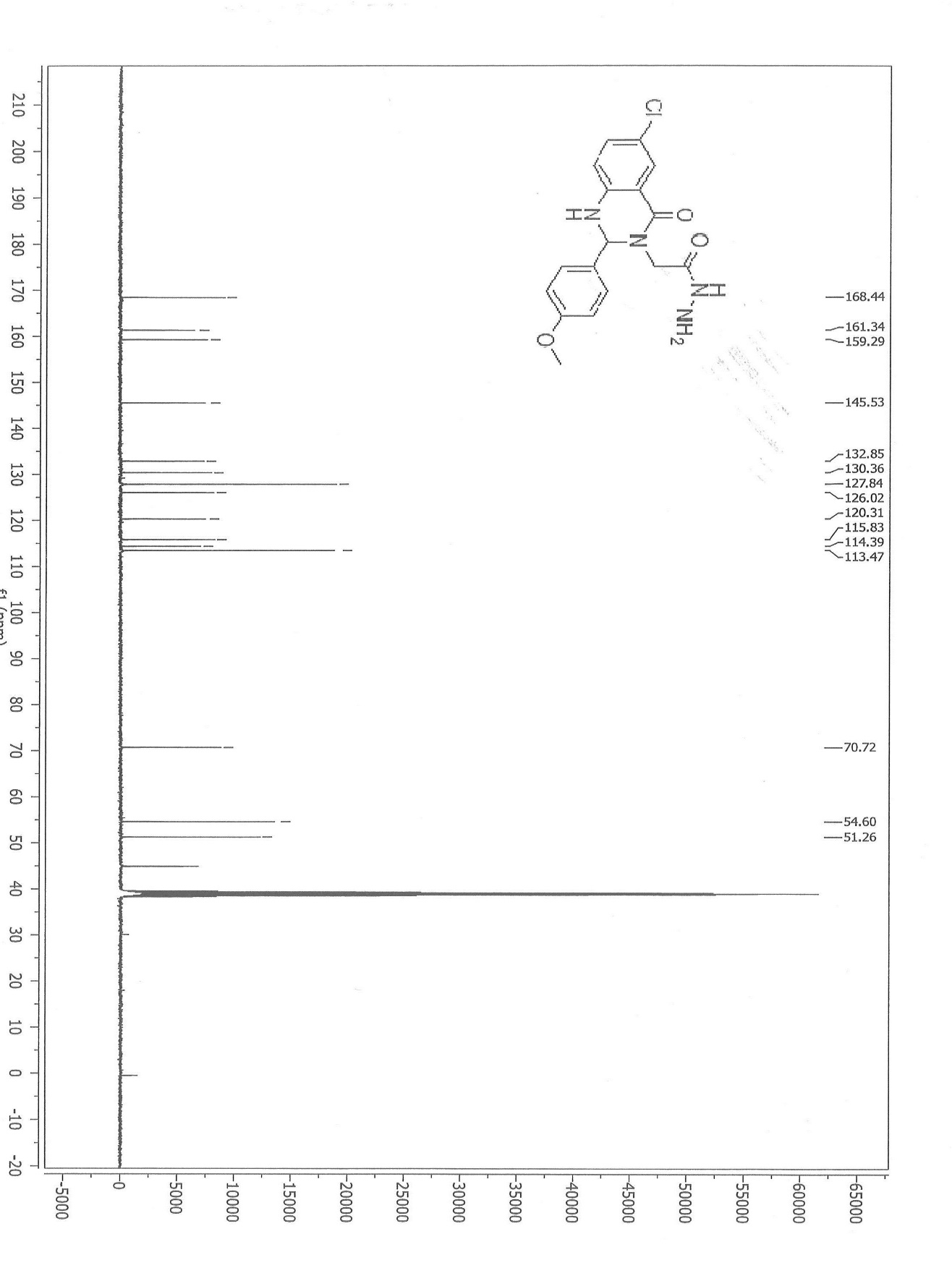


Figure S26. C^13^ spectra of compound (4c)


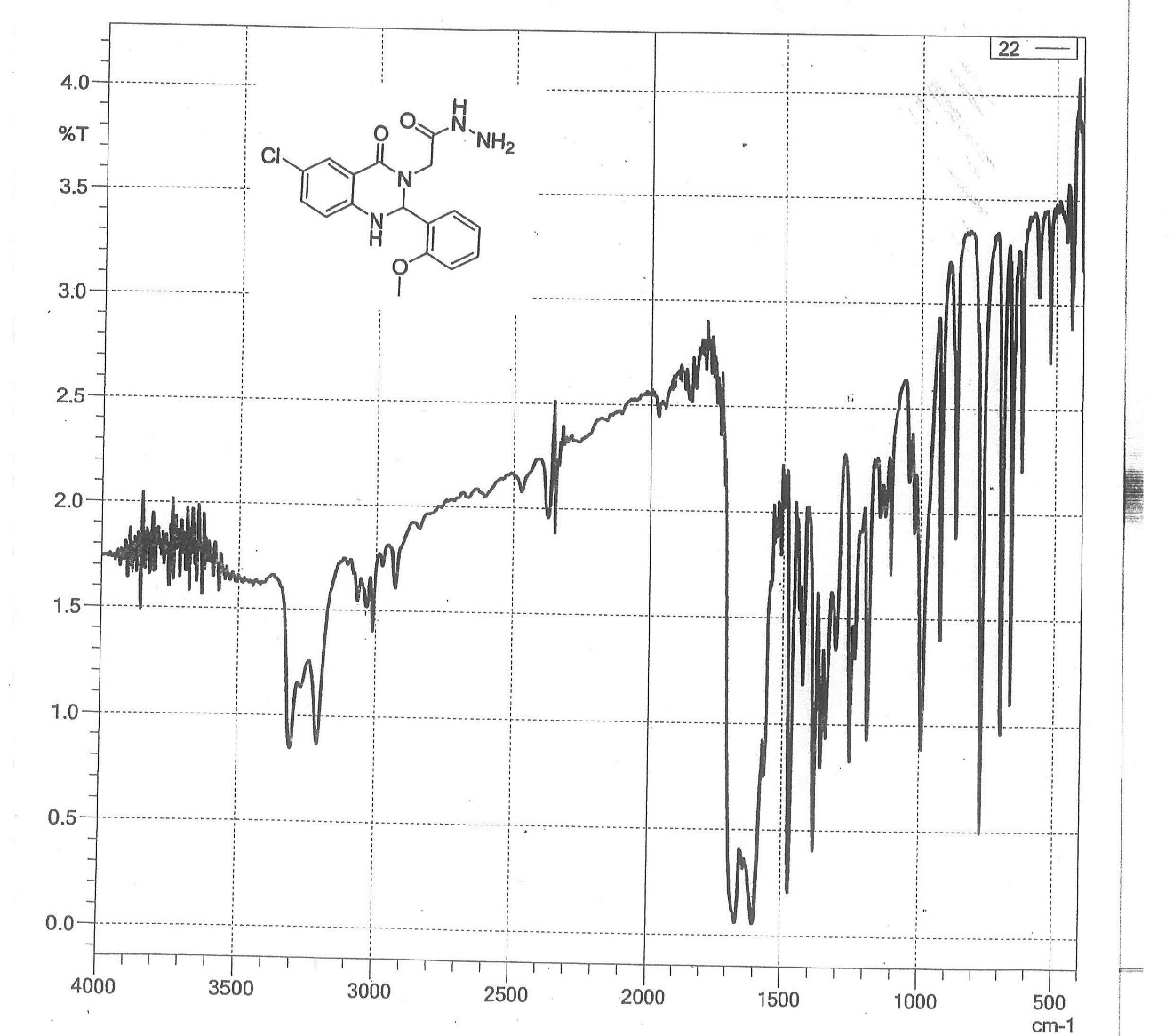


Figure S27. IR spectra of compound (4d)


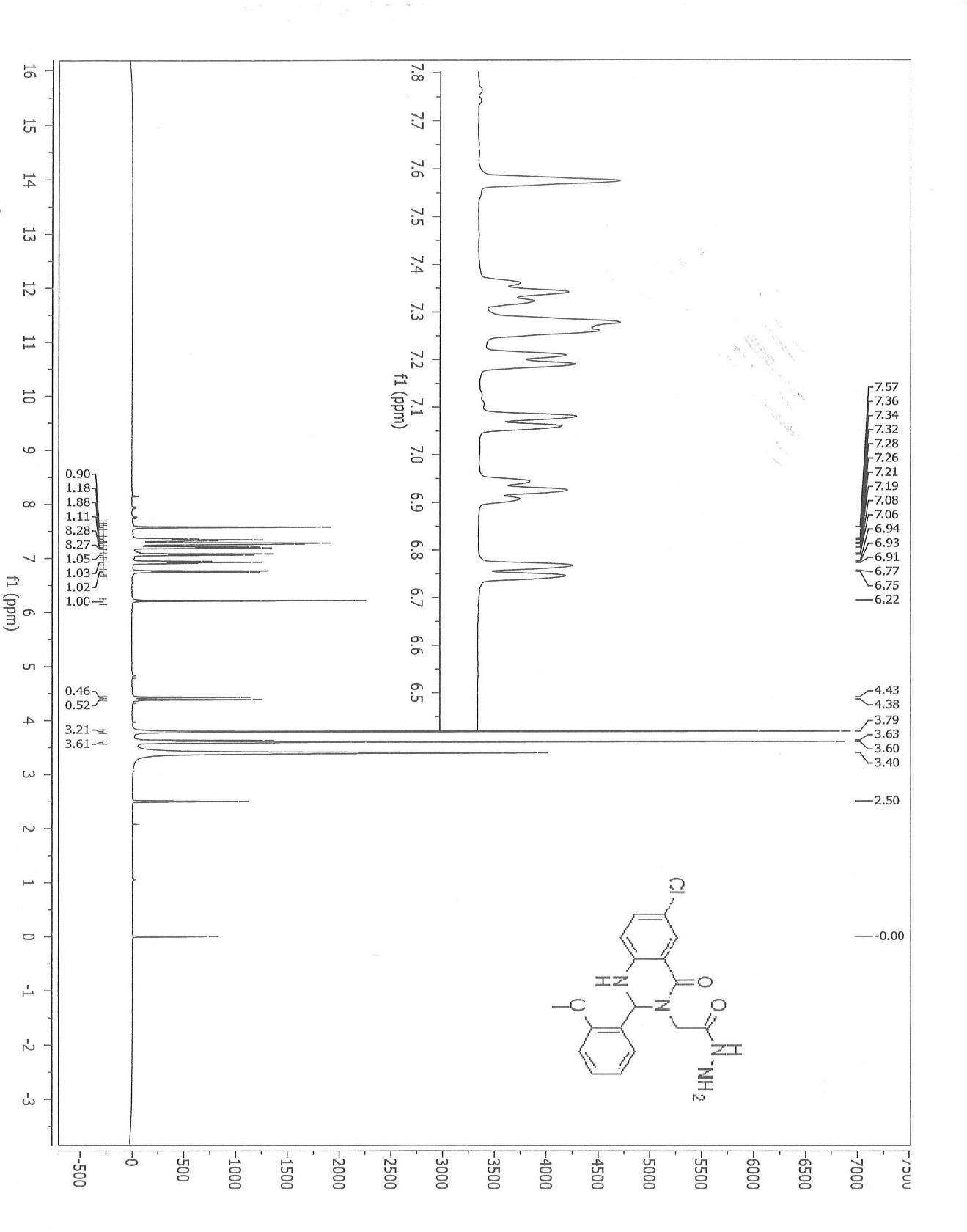


Figure S28. H^1^ spectra of compound (4d)


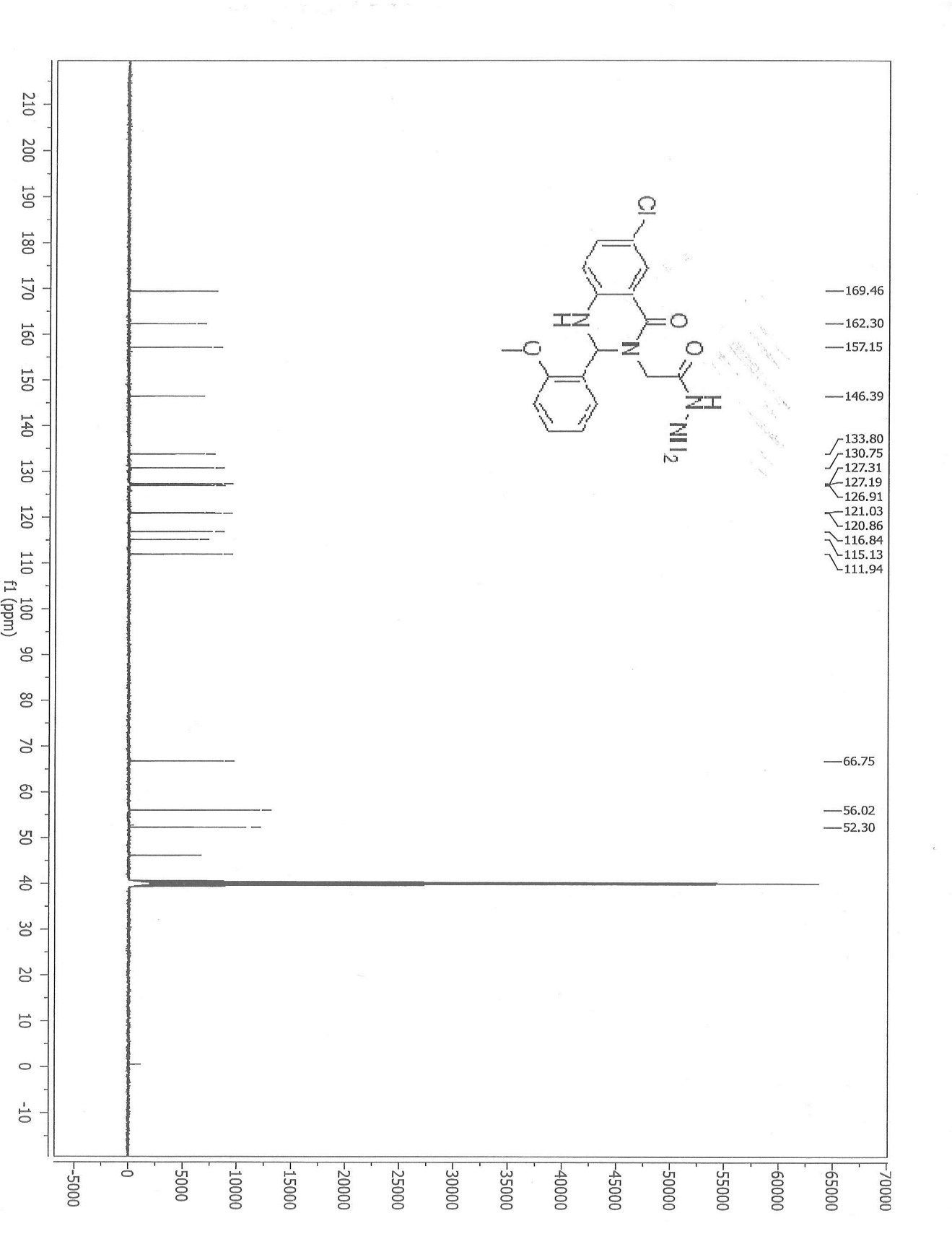


Figure S29. C^13^ spectra of compound (4d)


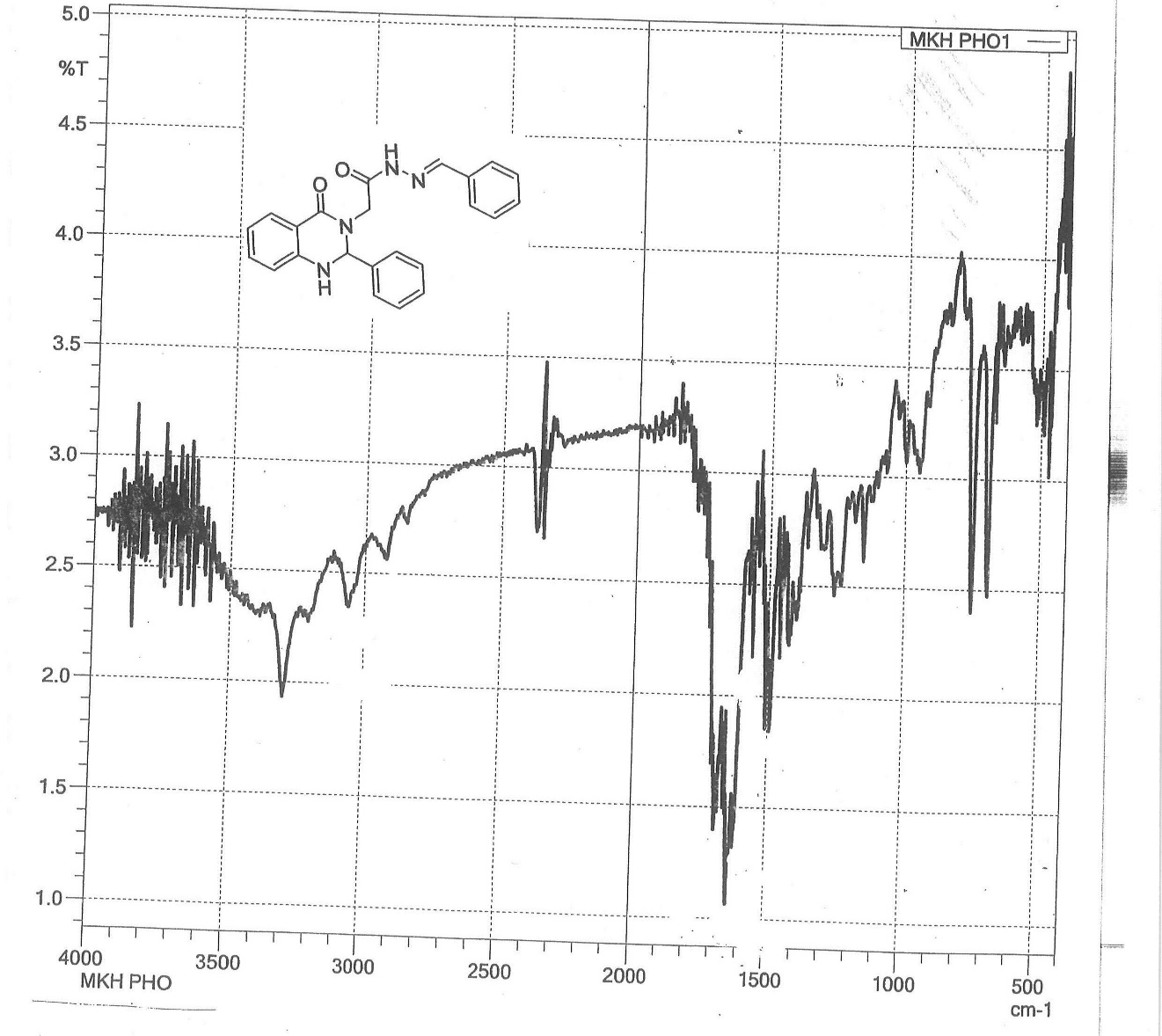


Figure S30. IR spectra of compound (5a)


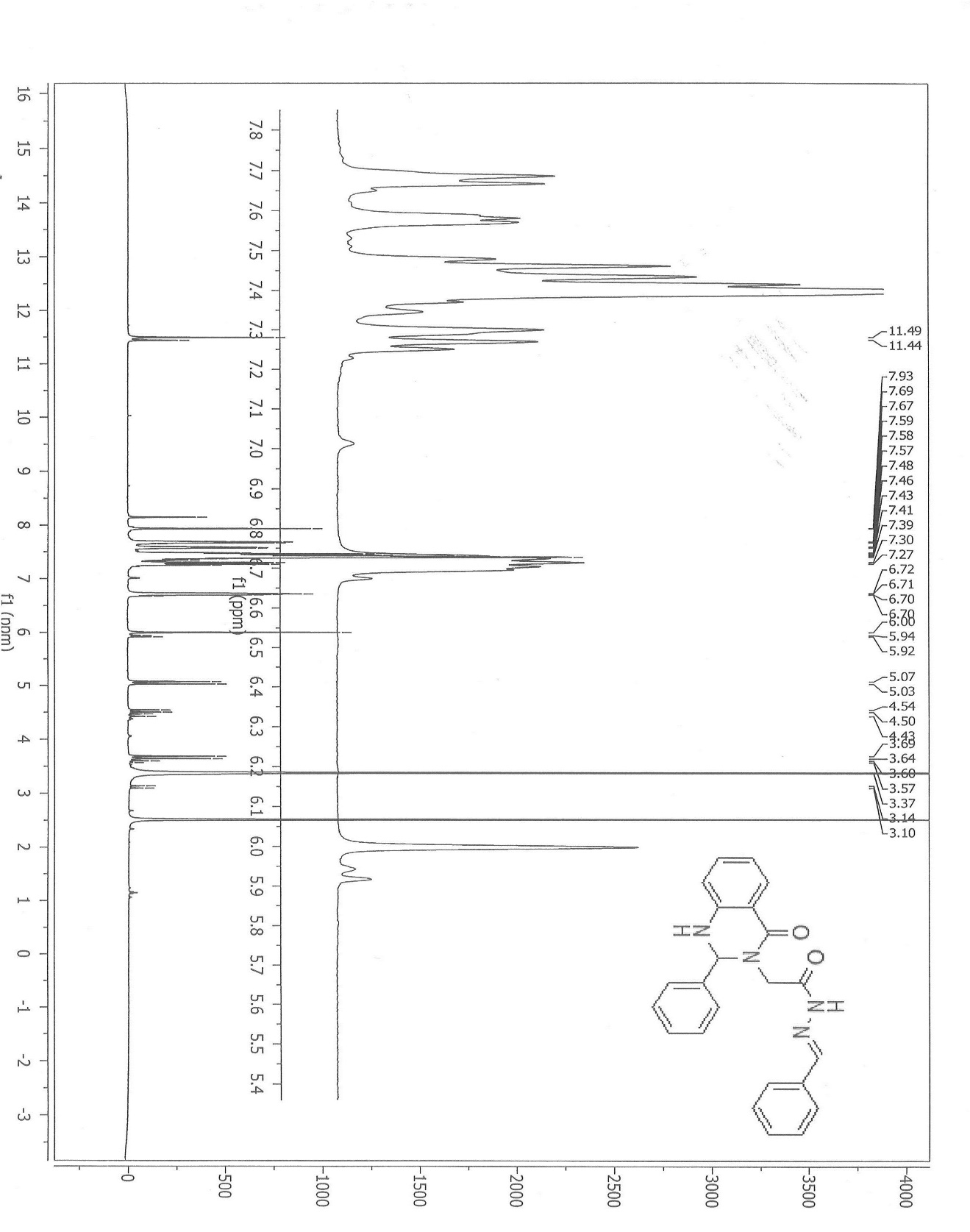


Figure S31. H^1^ spectra of compound (5a)


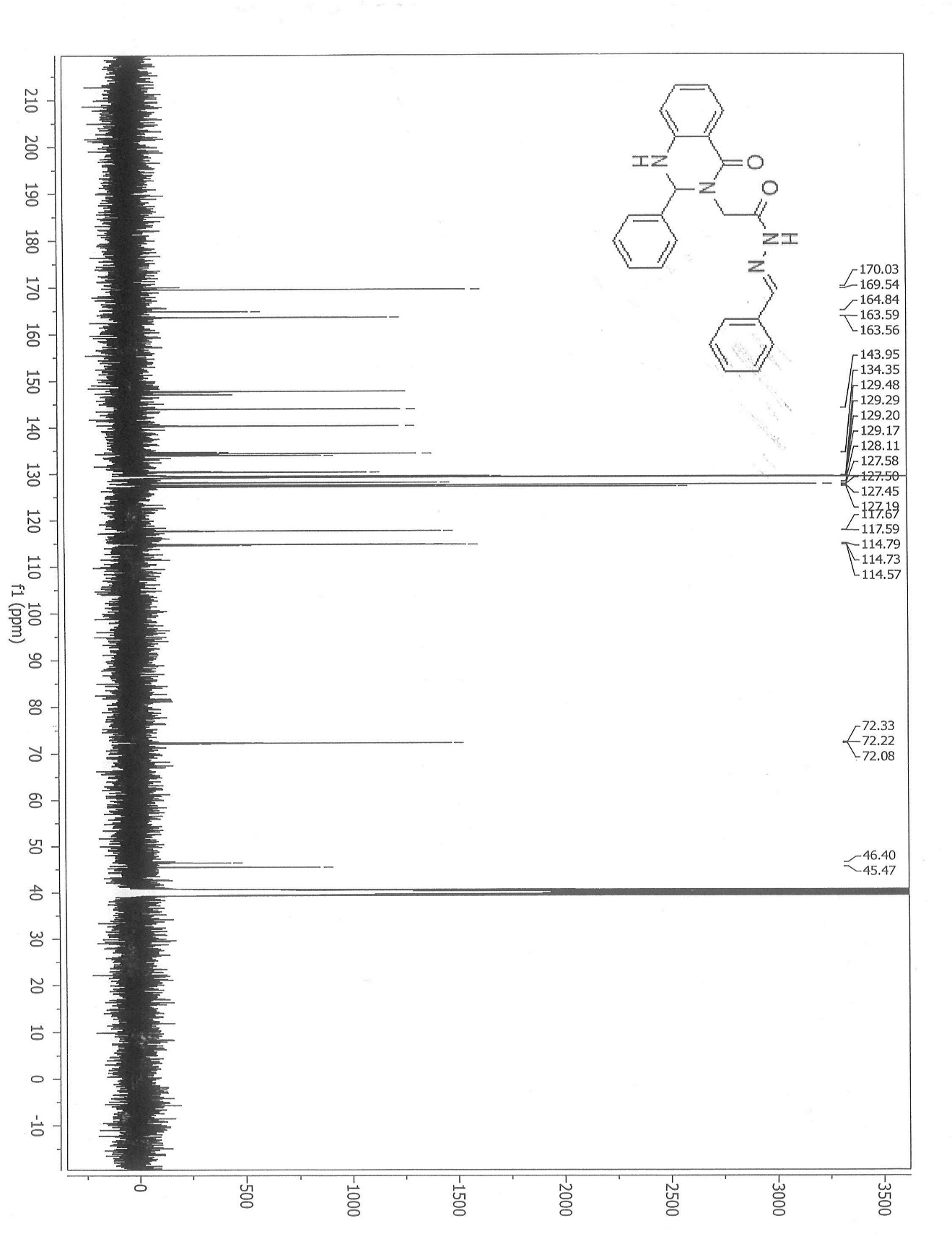


Figure S32. C^13^ spectra of compound (5a)


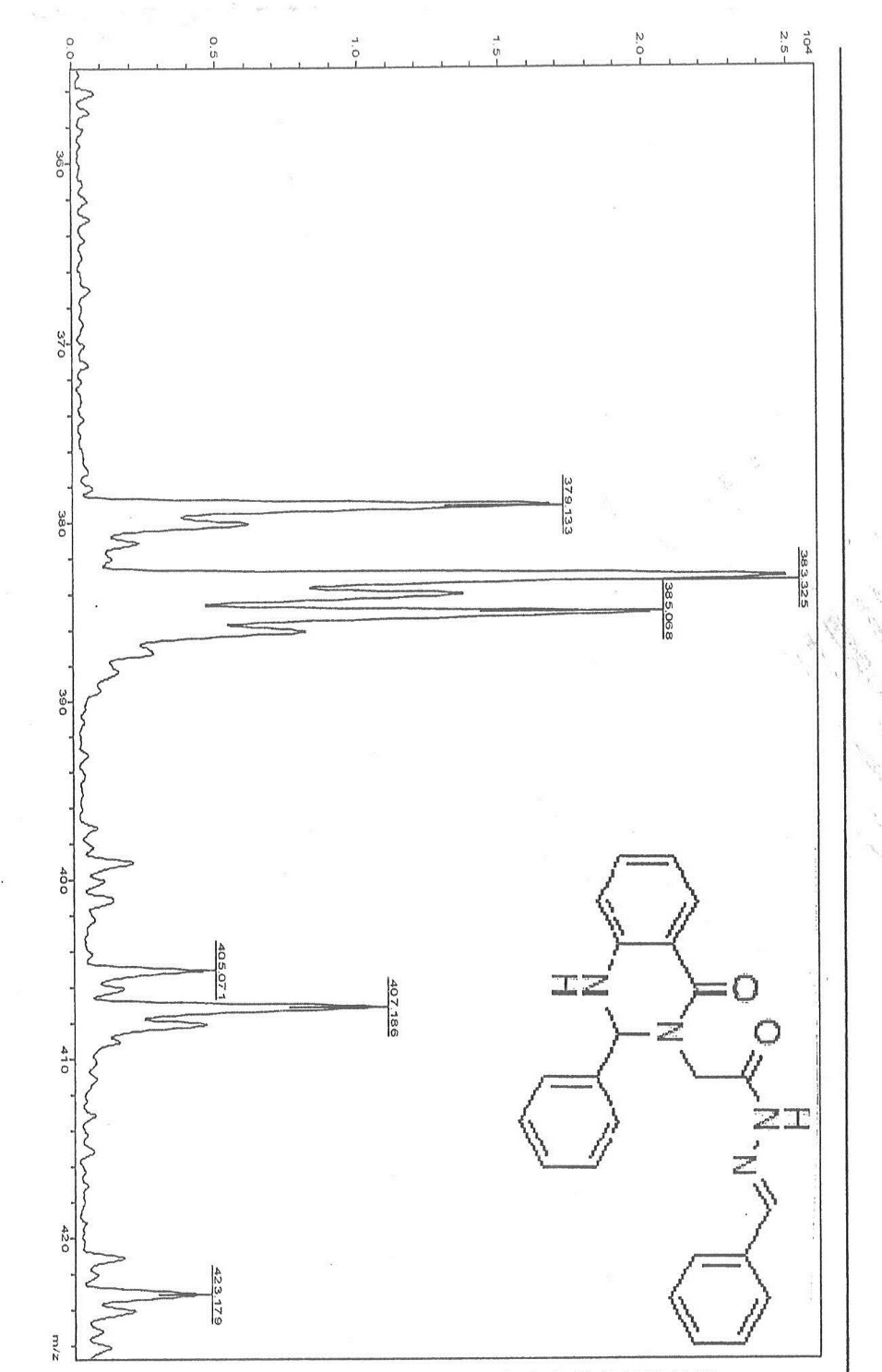


Figure S33. M.S spectra of compound (5a)


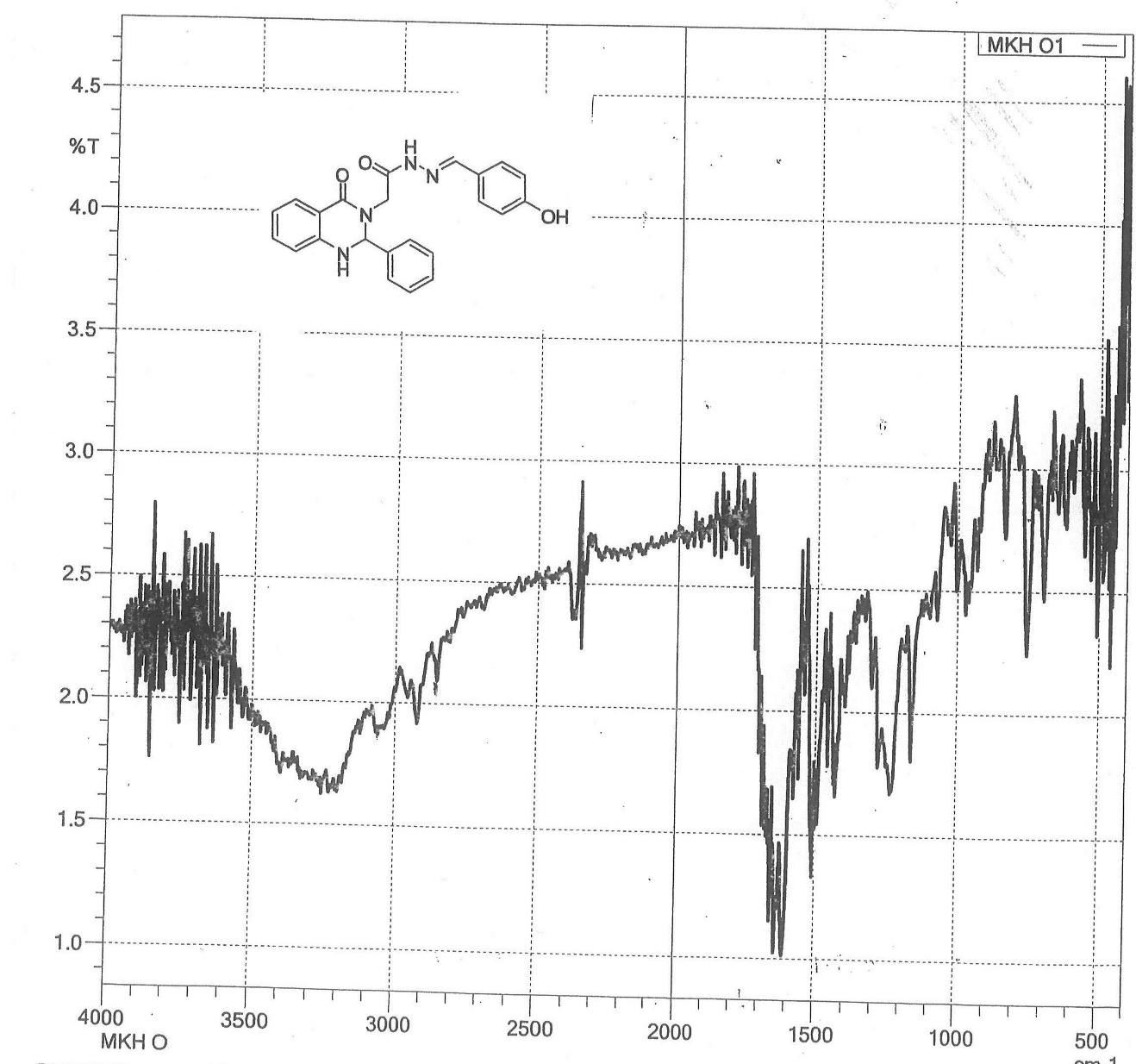


Figure S34. IR spectra of compound (5b)


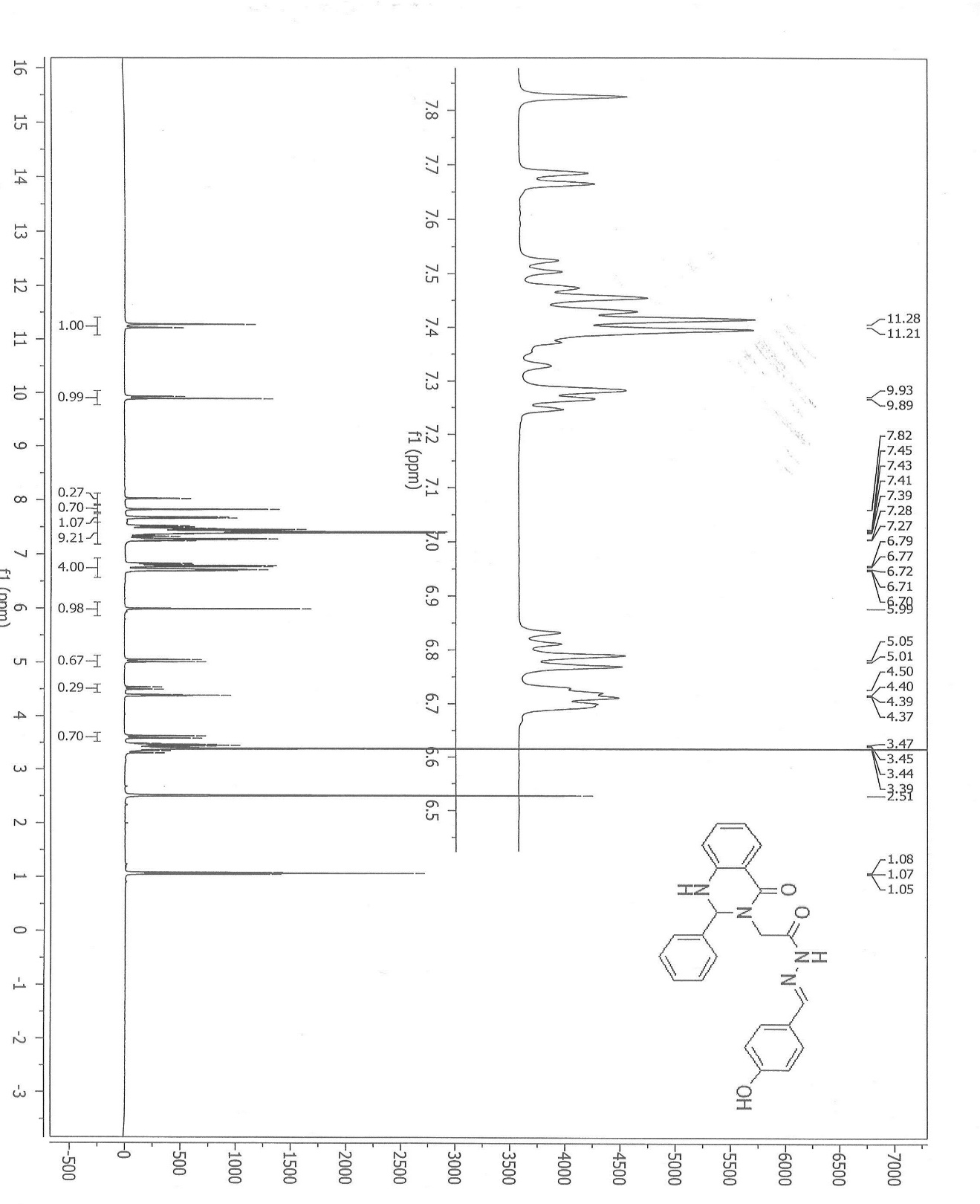


Figure S35. H^1^ spectra of compound (5b)


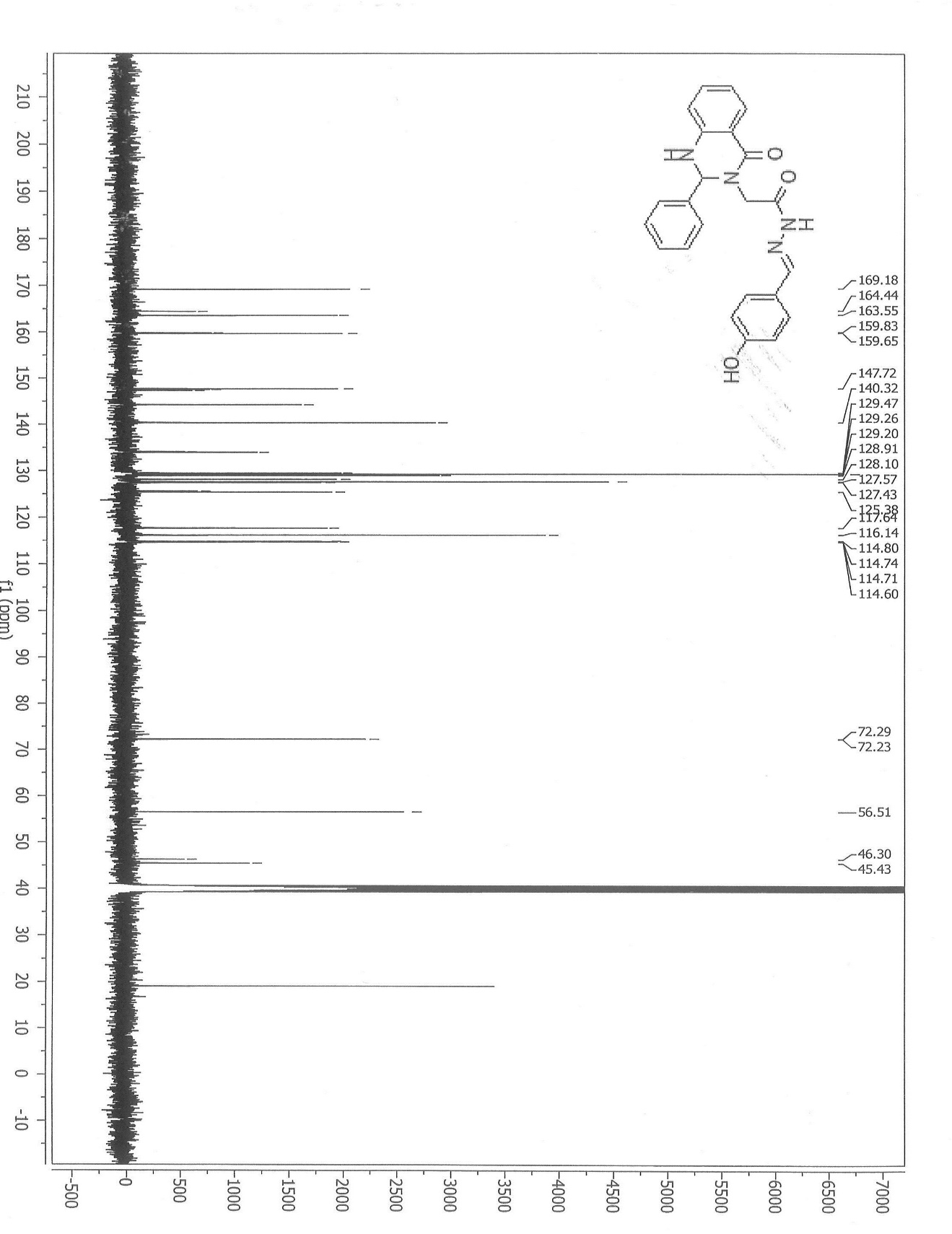


Figure S36. C^13^ spectra of compound (5b)


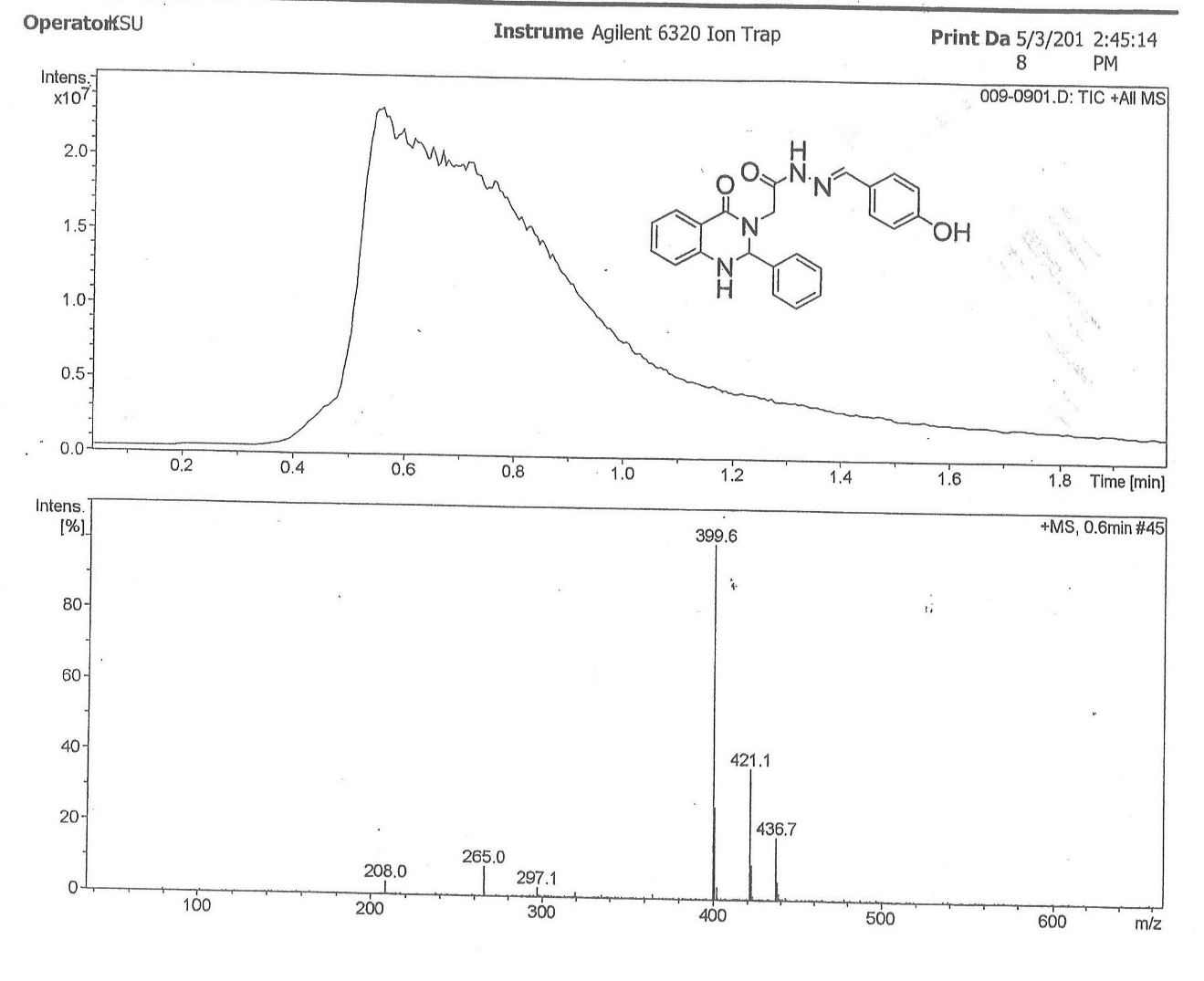


Figure S37. MS-ESI spectra of compound (5b)


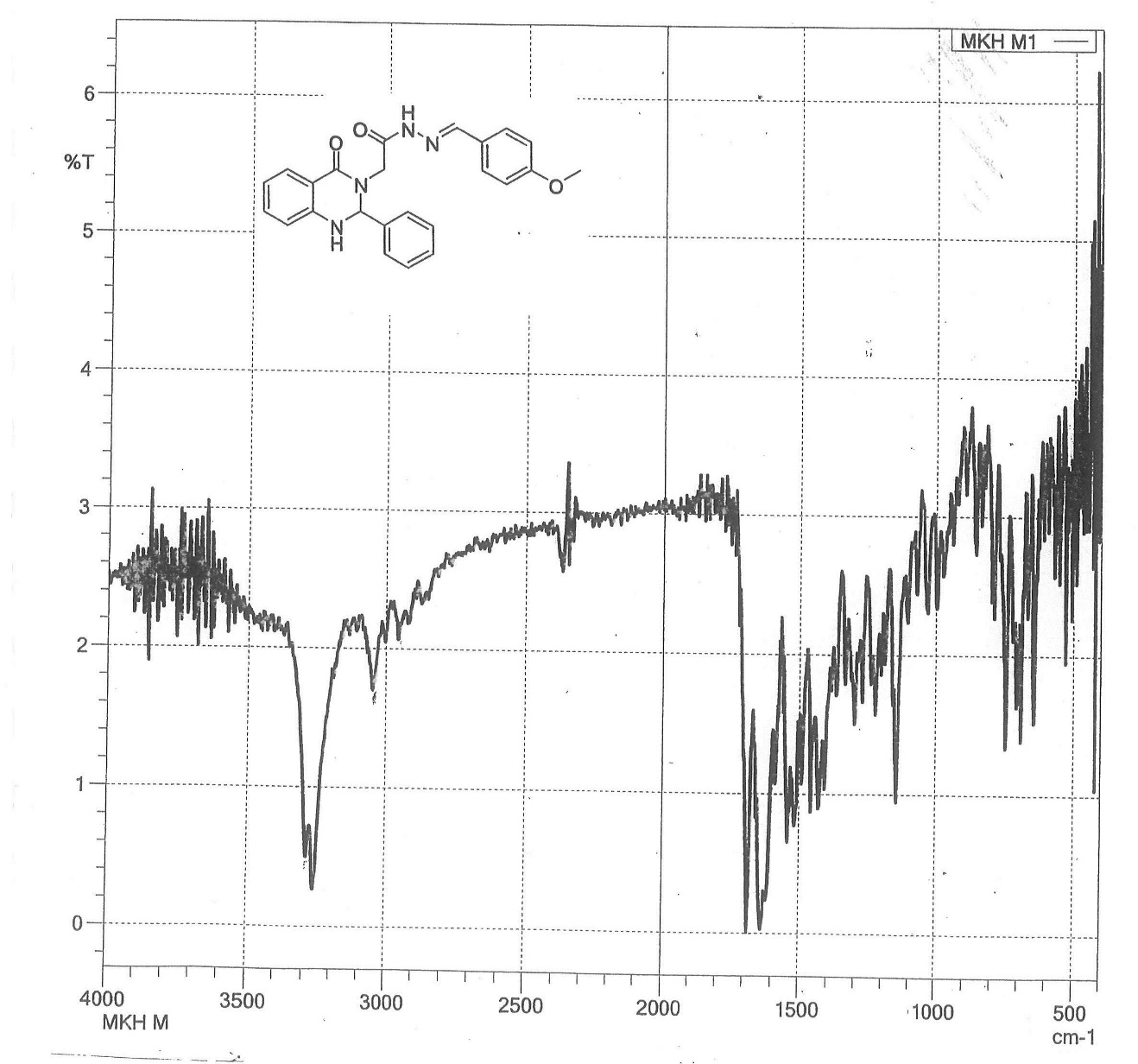


Figure S38. IR spectra of compound (5c)


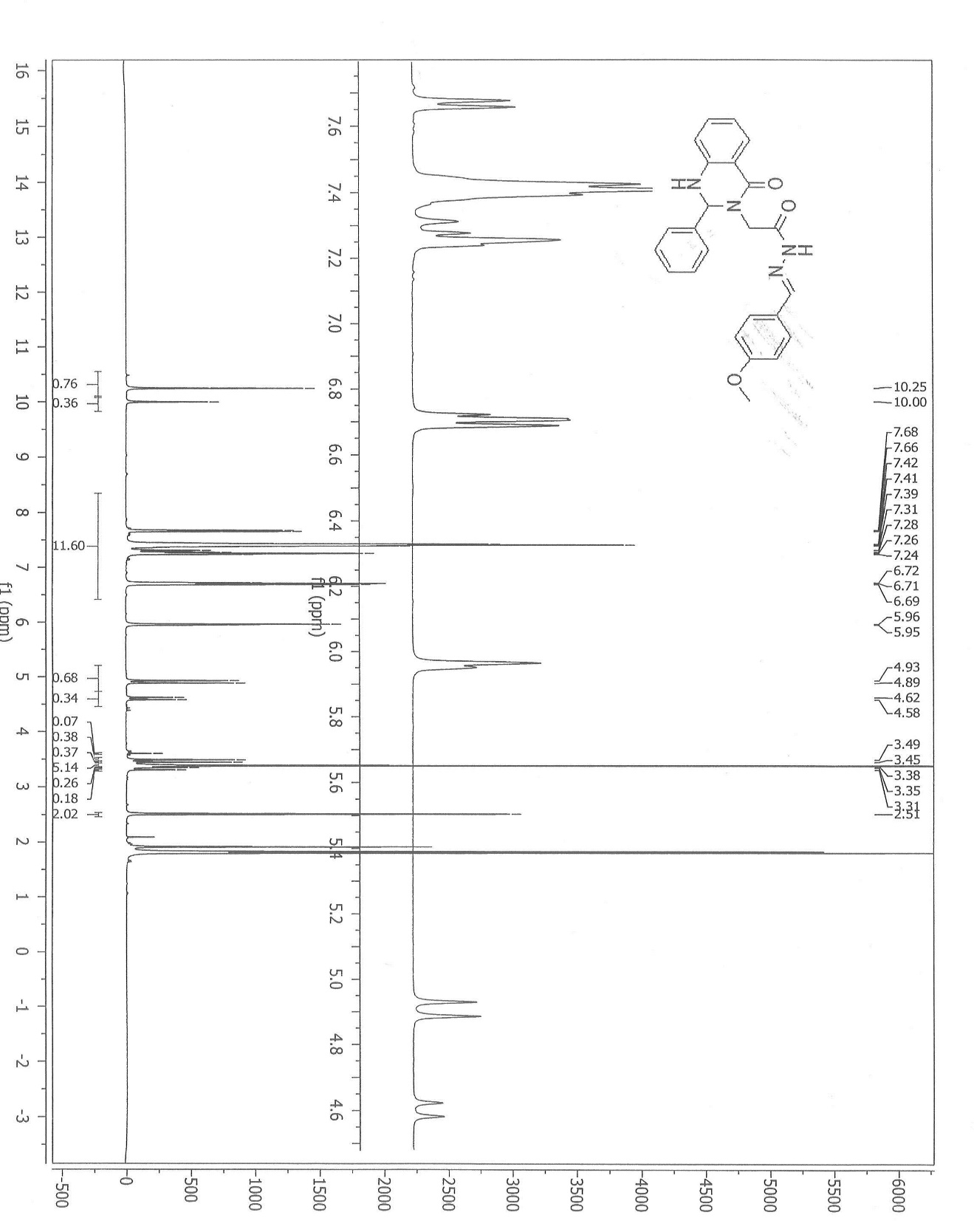


Figure S39. H^1^ spectra of compound (5c)


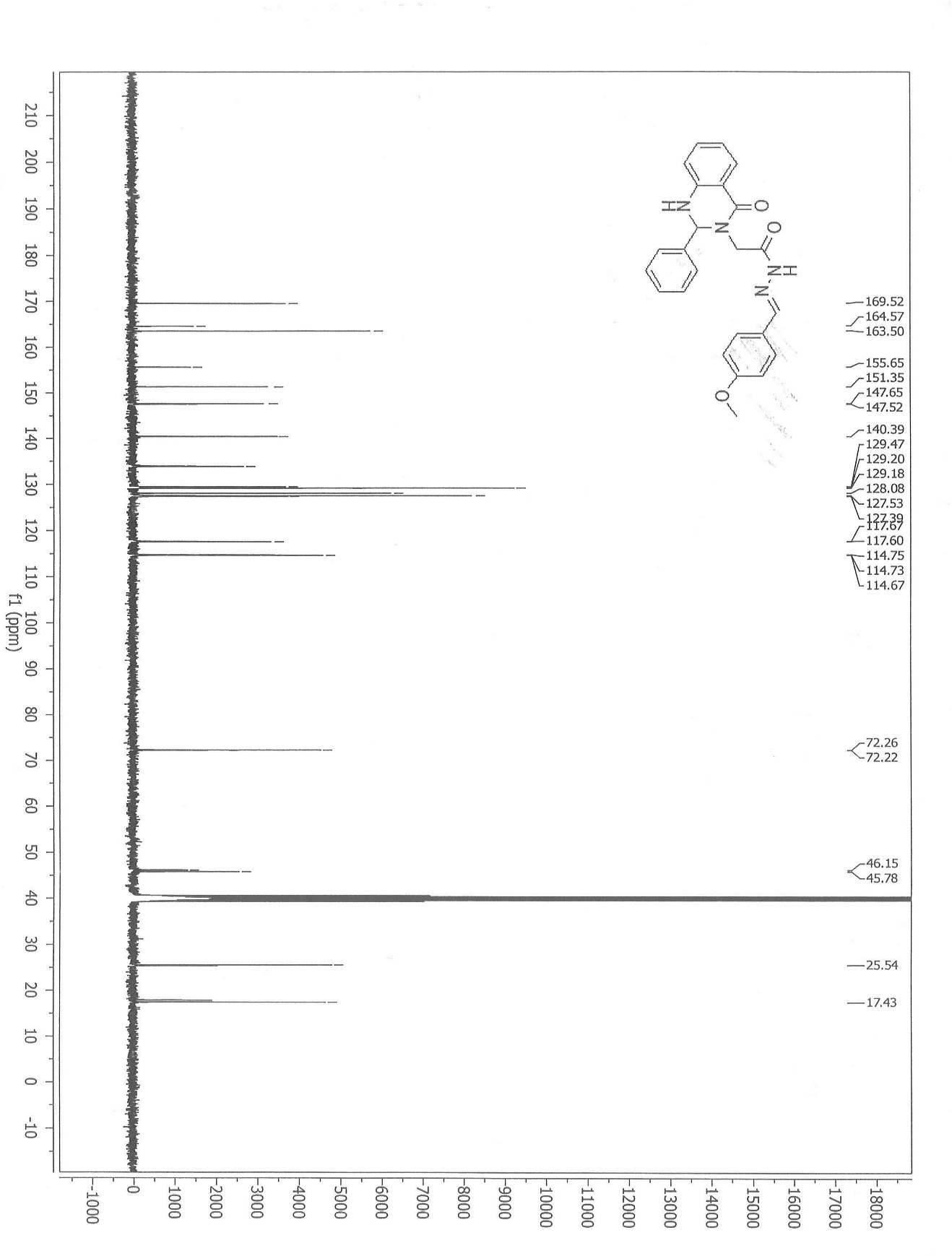


Figure S40. C^13^ spectra of compound (5c)


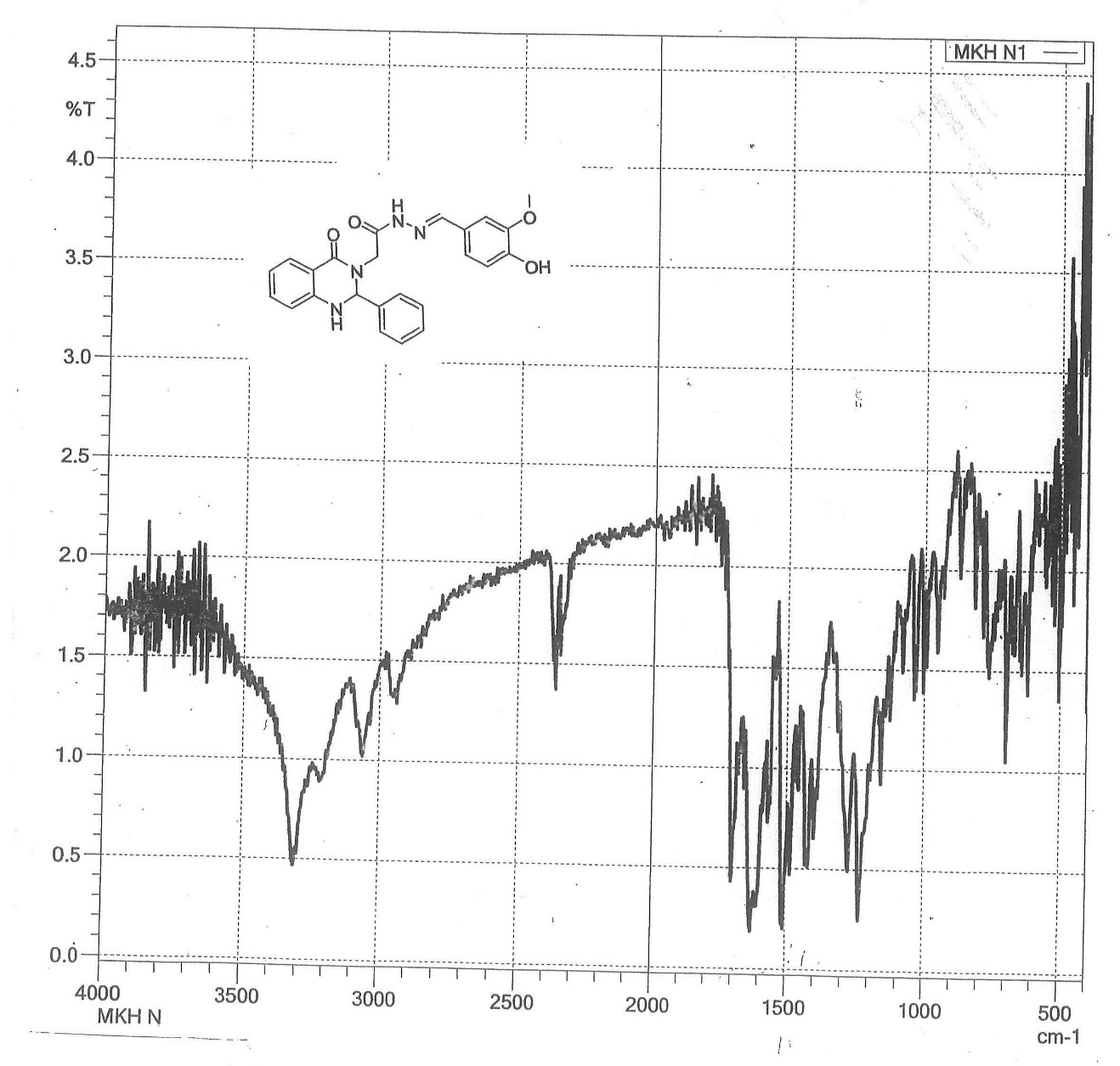


Figure S41. IR spectra of compound (5d)


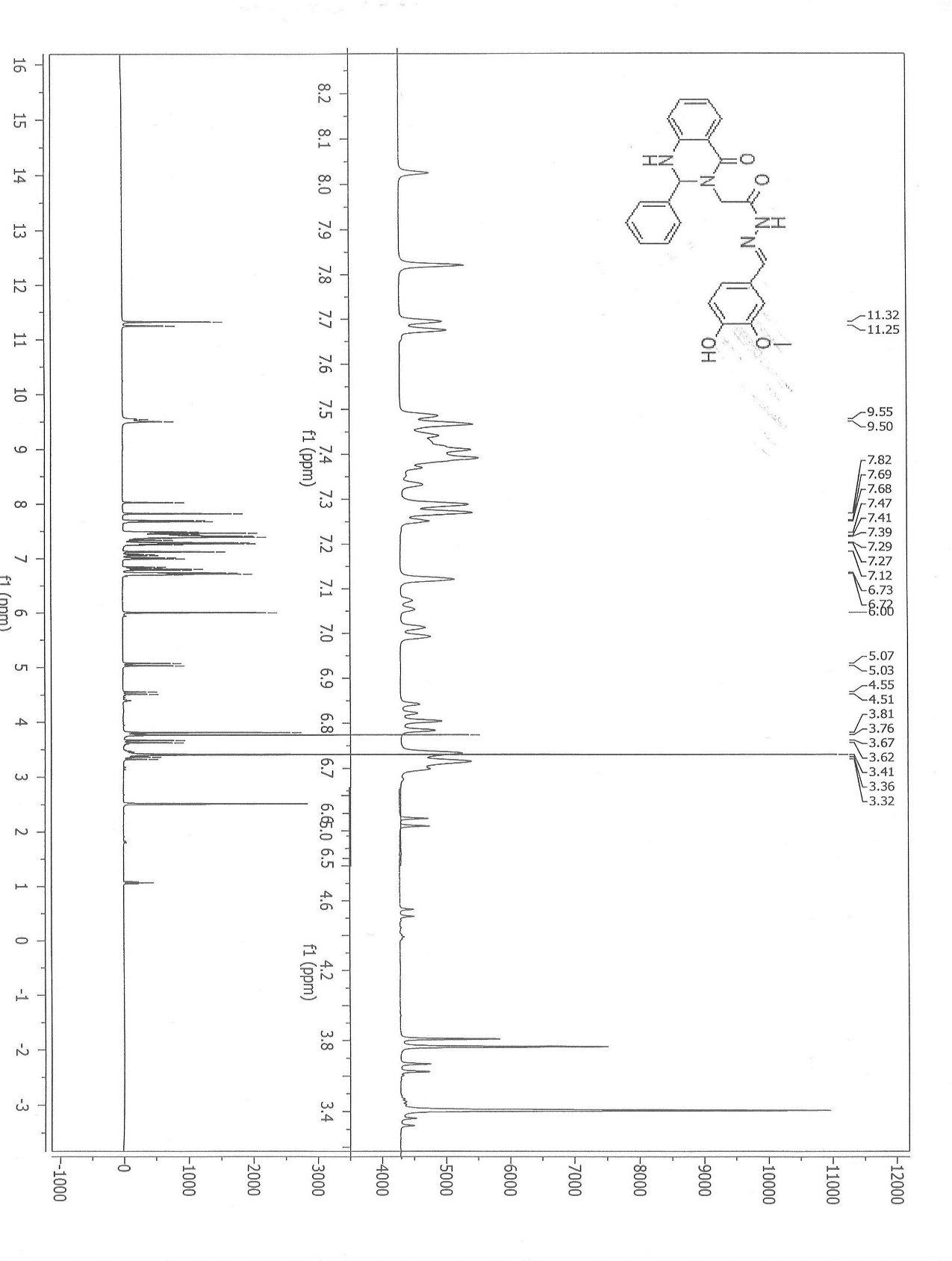


Figure S42. H^1^ spectra of compound (5d)


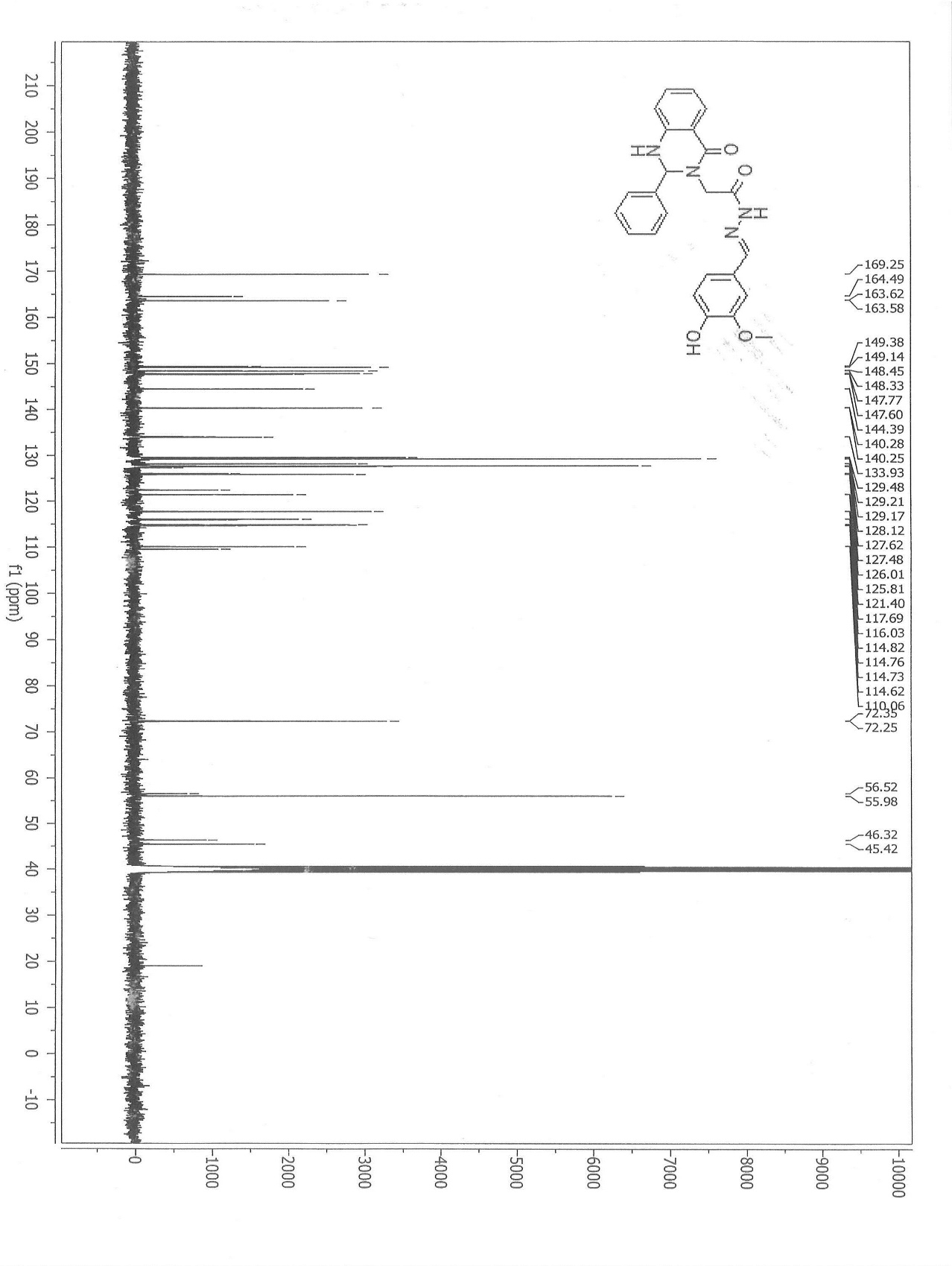


Figure S43. C^13^ spectra of compound (5d)


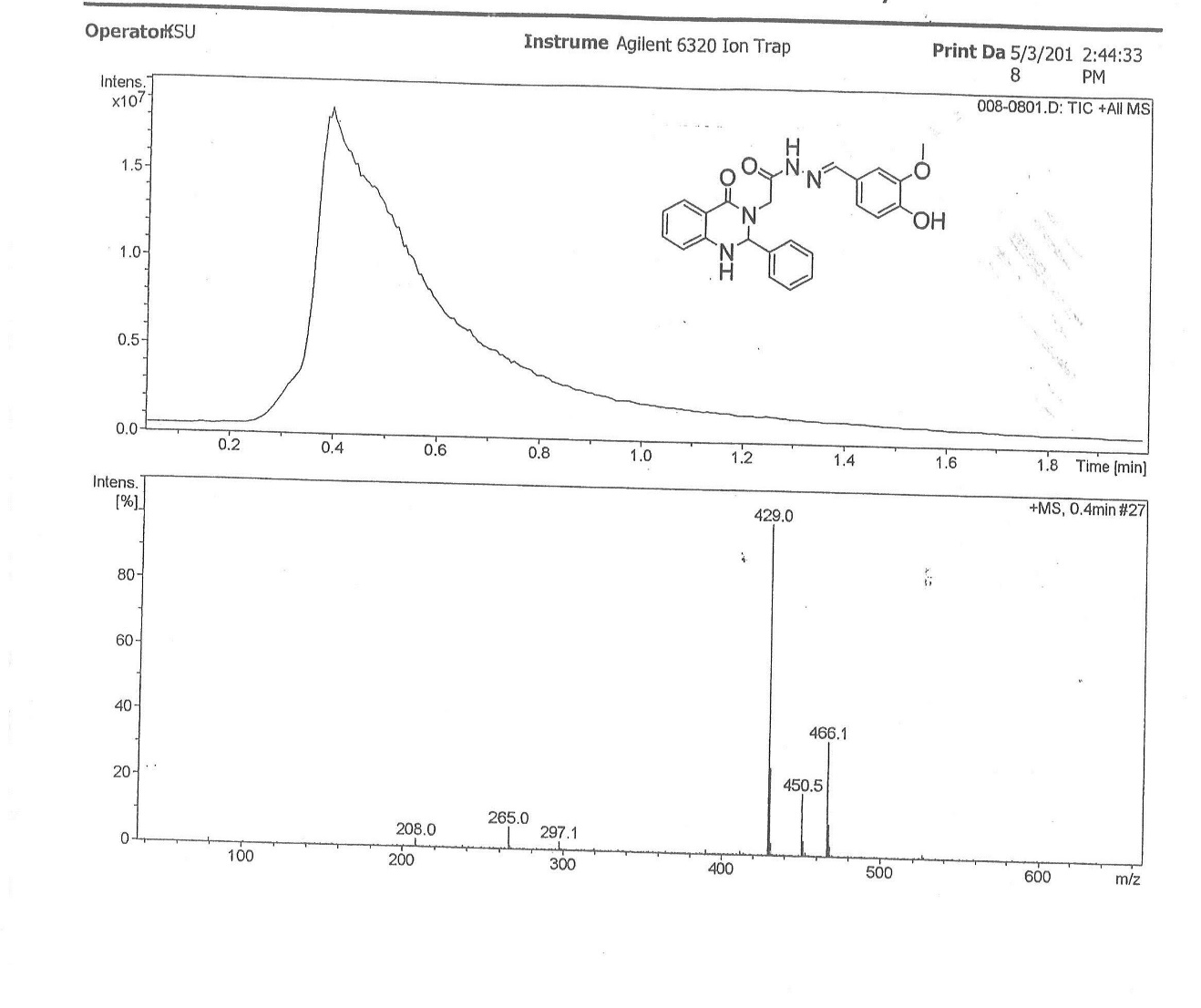


Figure S44. MS-ESI spectra of compound (5d)


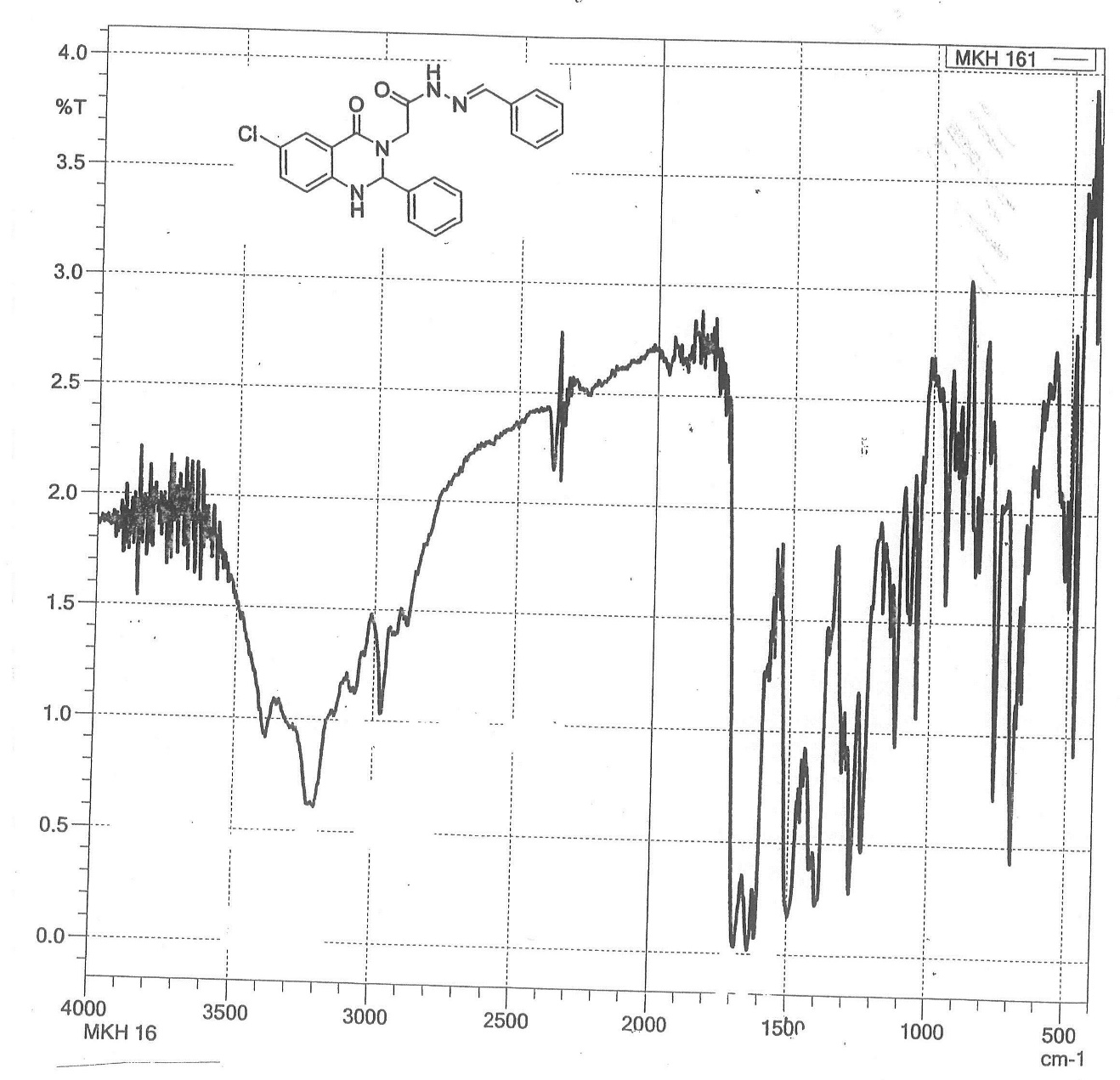


Figure S45. IR spectra of compound (5e)


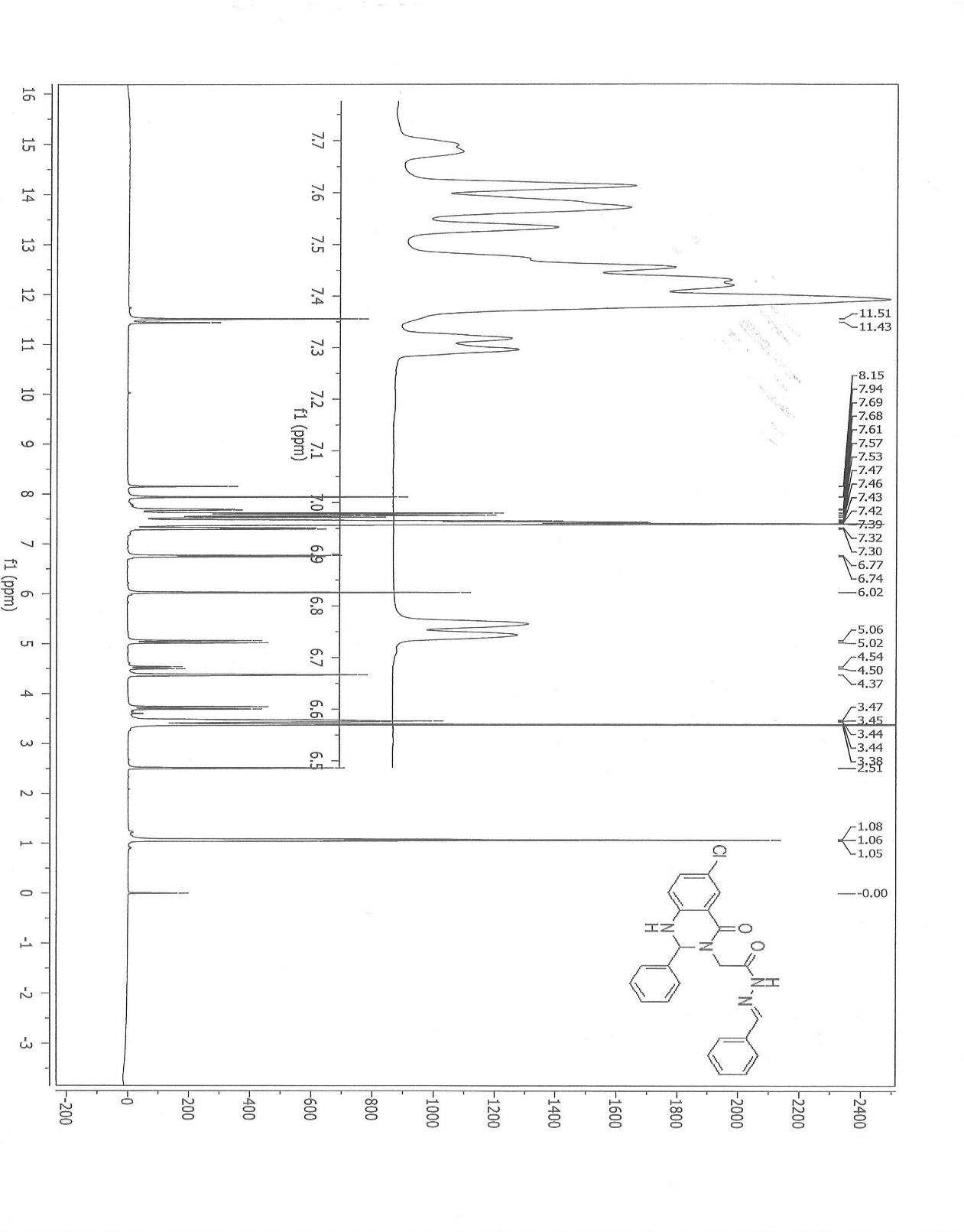


Figure S46. H^1^ spectra of compound (5e)


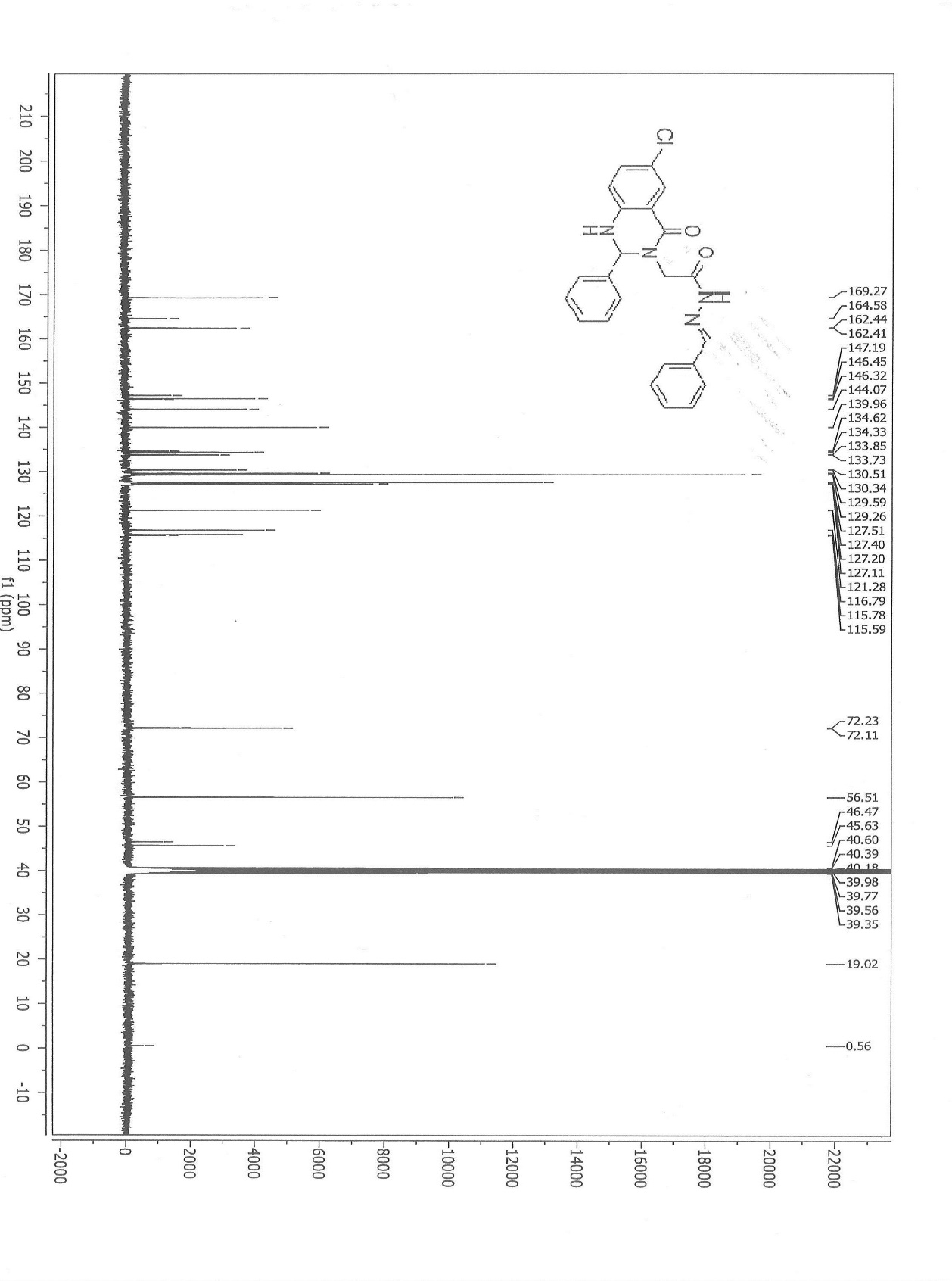


Figure S47. C^13^ spectra of compound (5e)


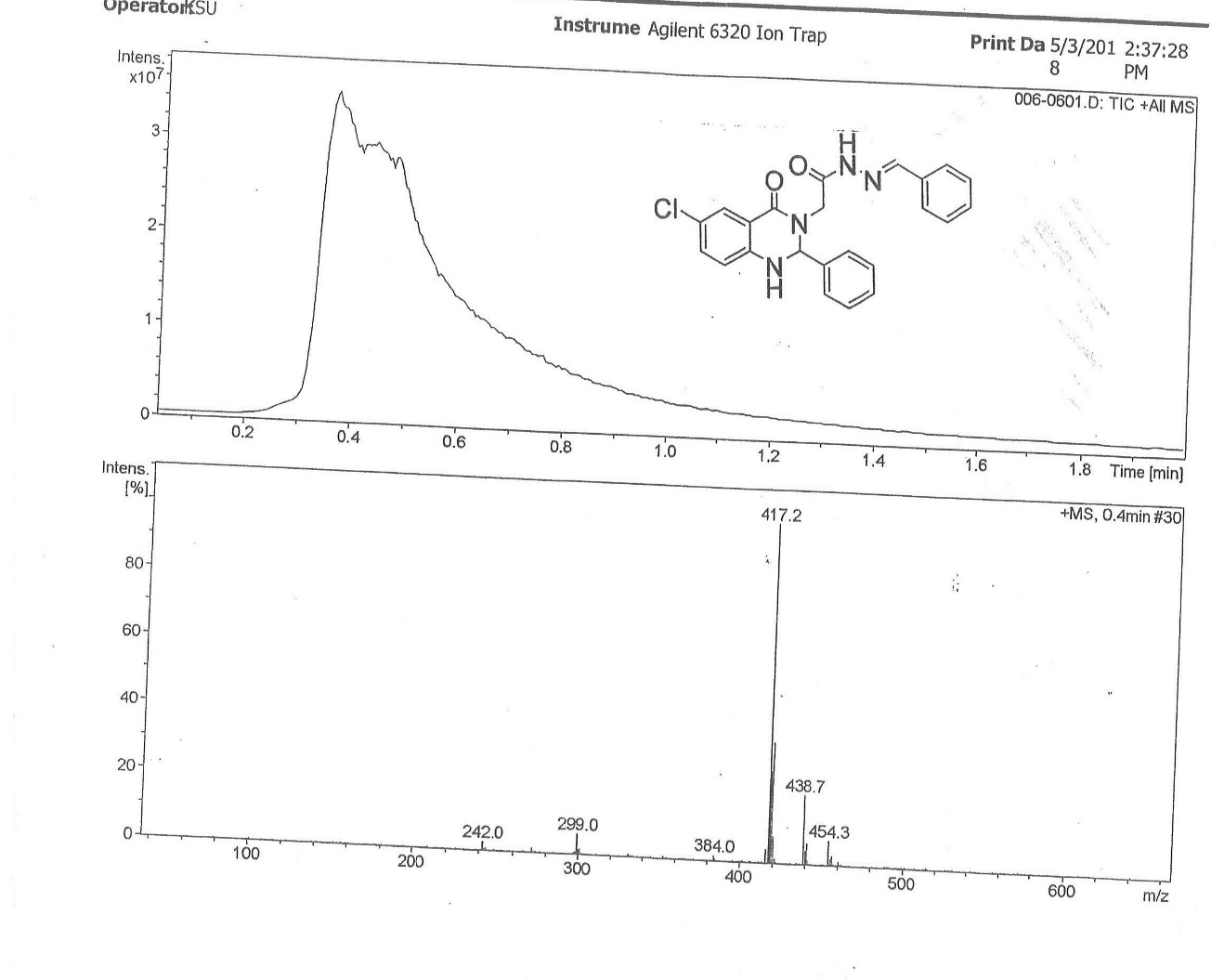


Figure S48. MS-ESI spectra of compound (5e)


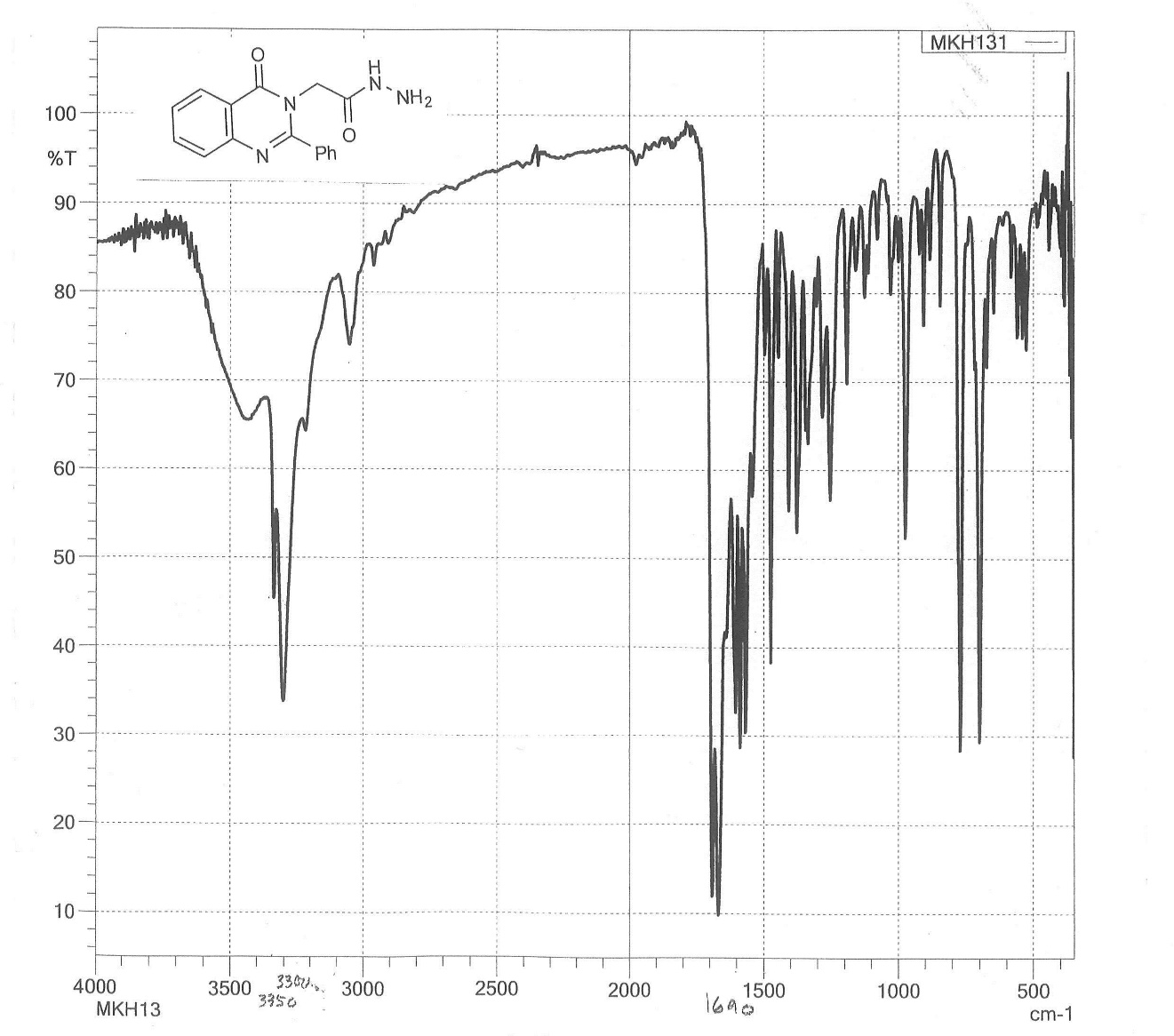


Figure S49. IR spectra of compound (7)


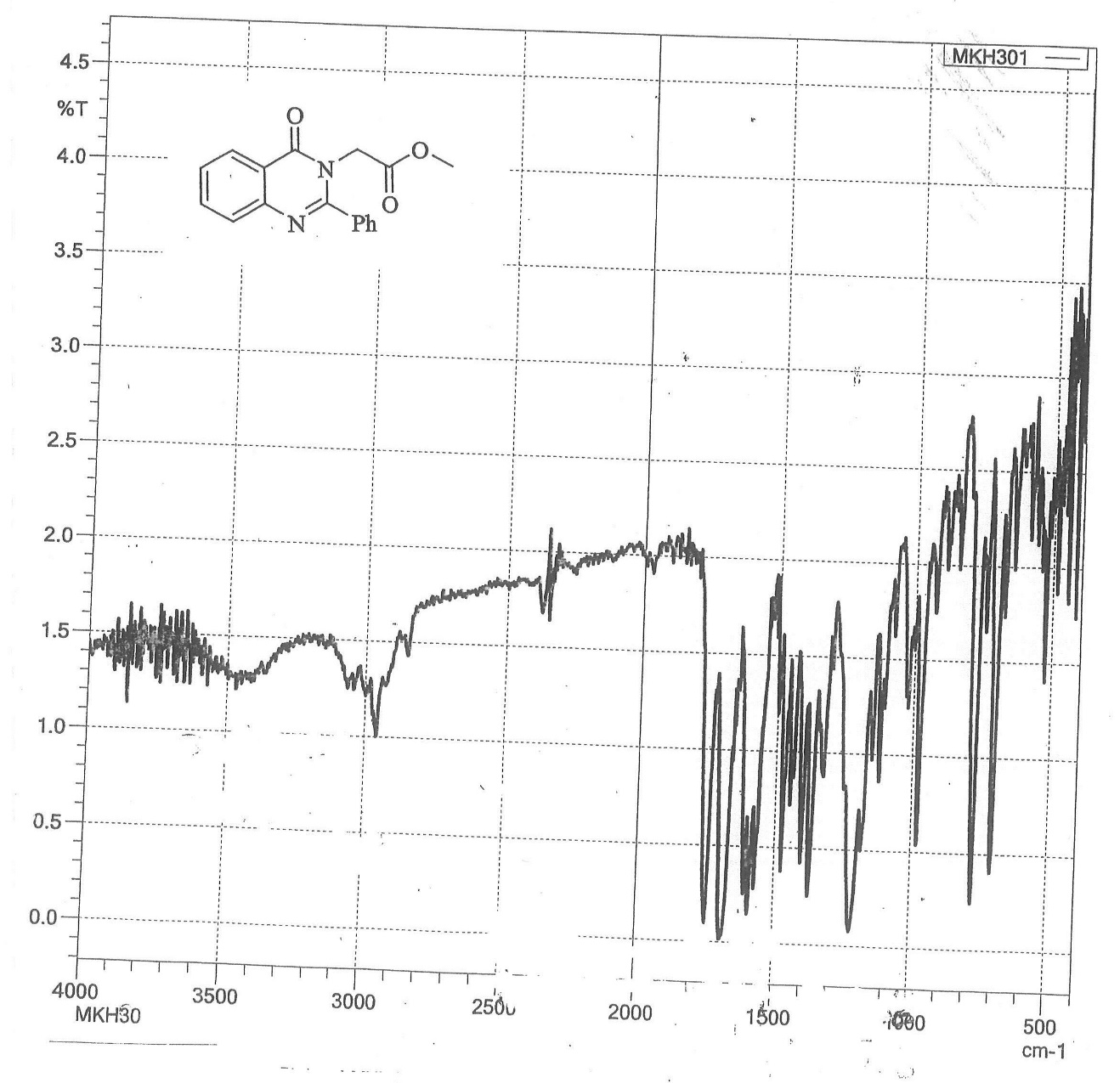


Figure S50. IR spectra of compound (8)


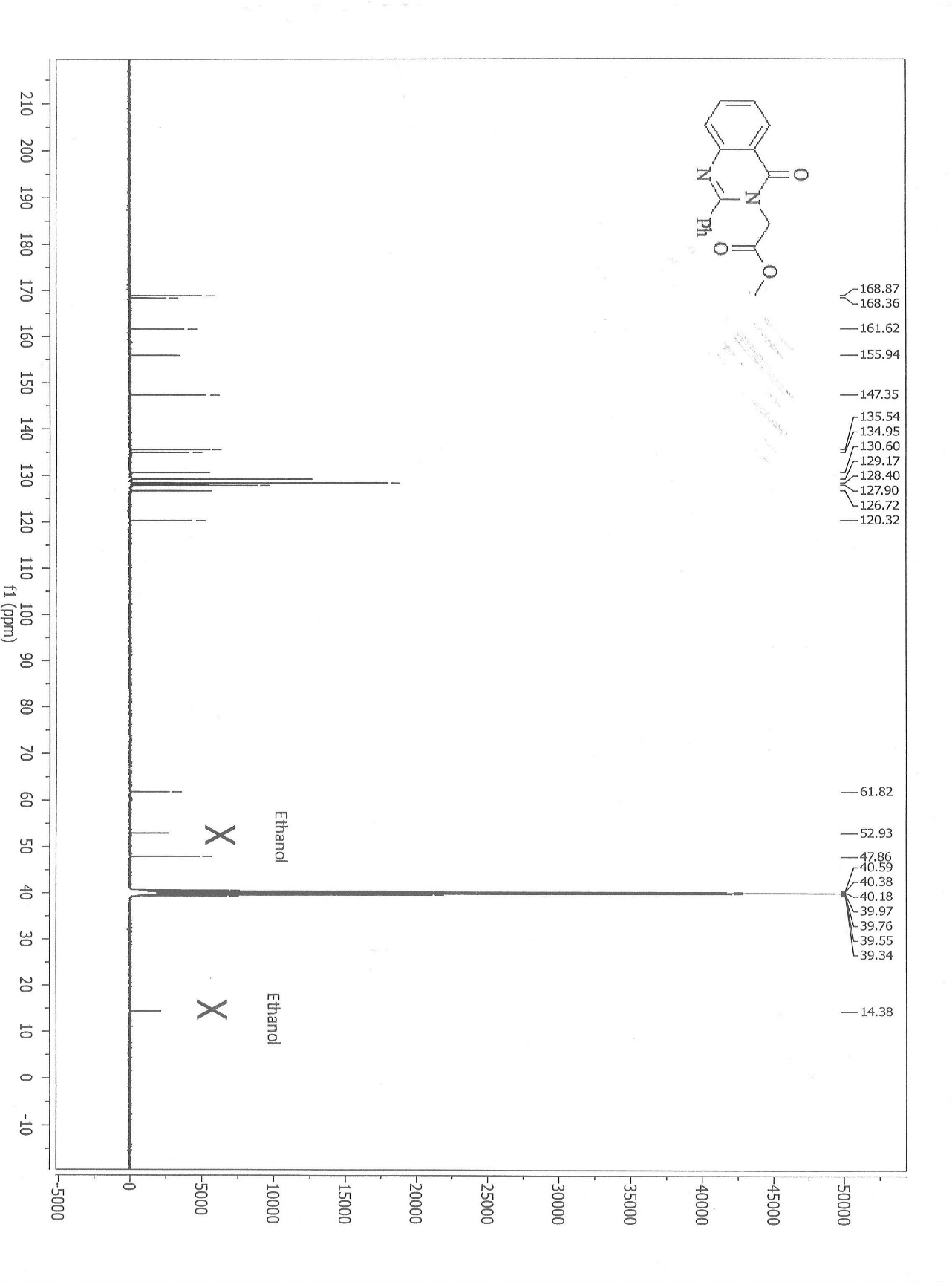


Figure S51. C^13^ spectra of compound (8)


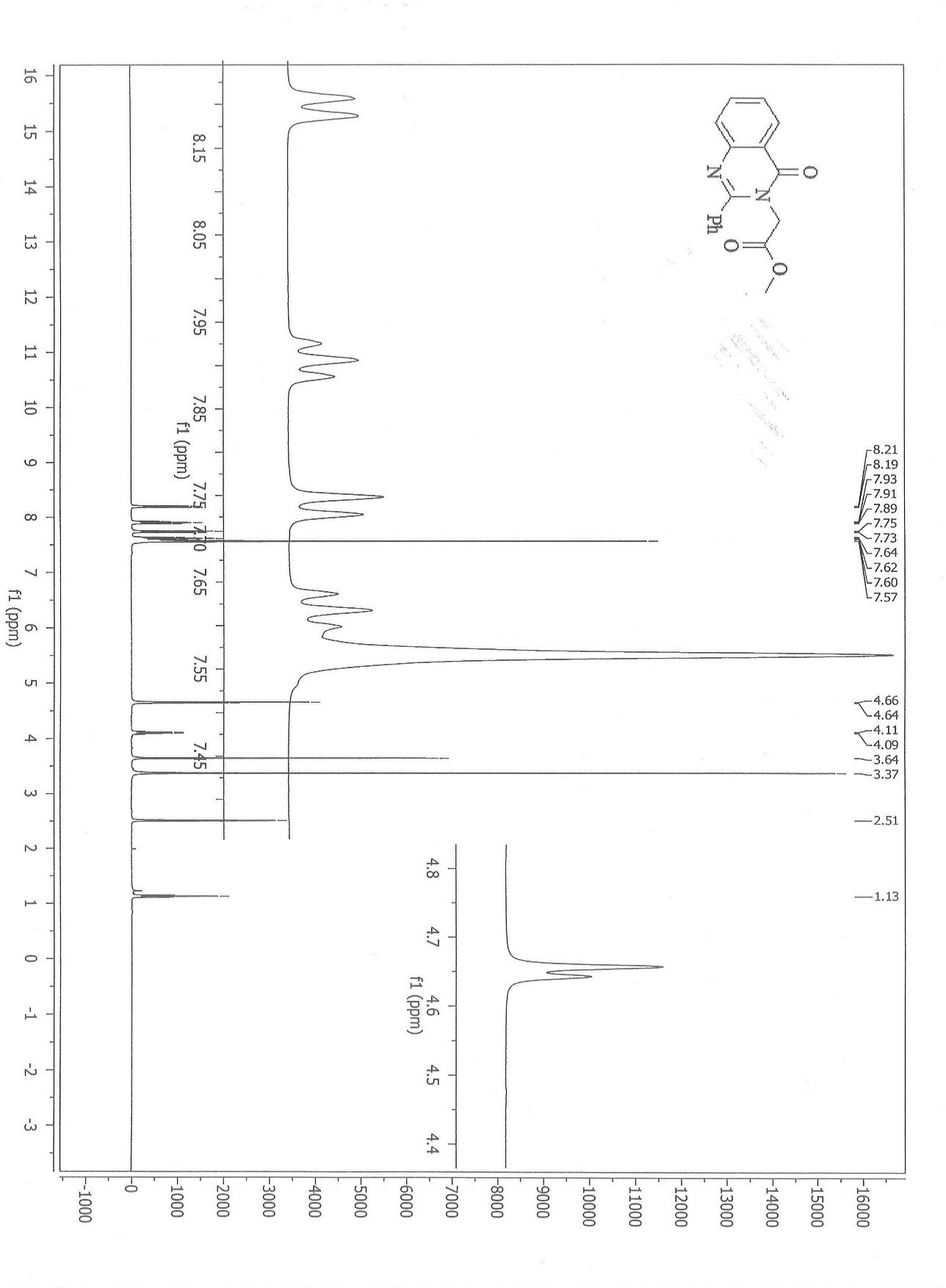


Figure S52. H^1^ spectra of compound (8)


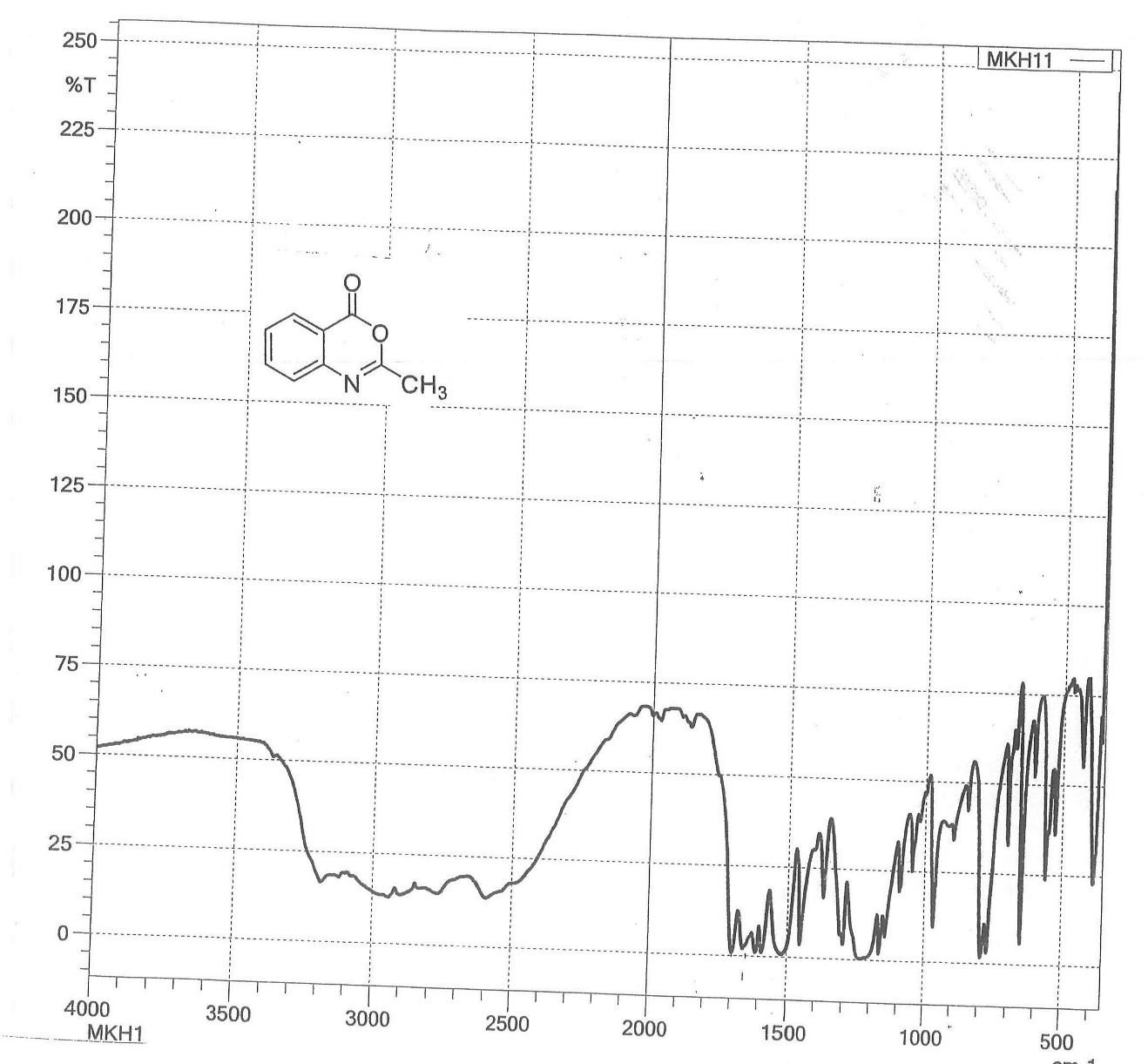


Figure S53. IR spectra of compound (9)


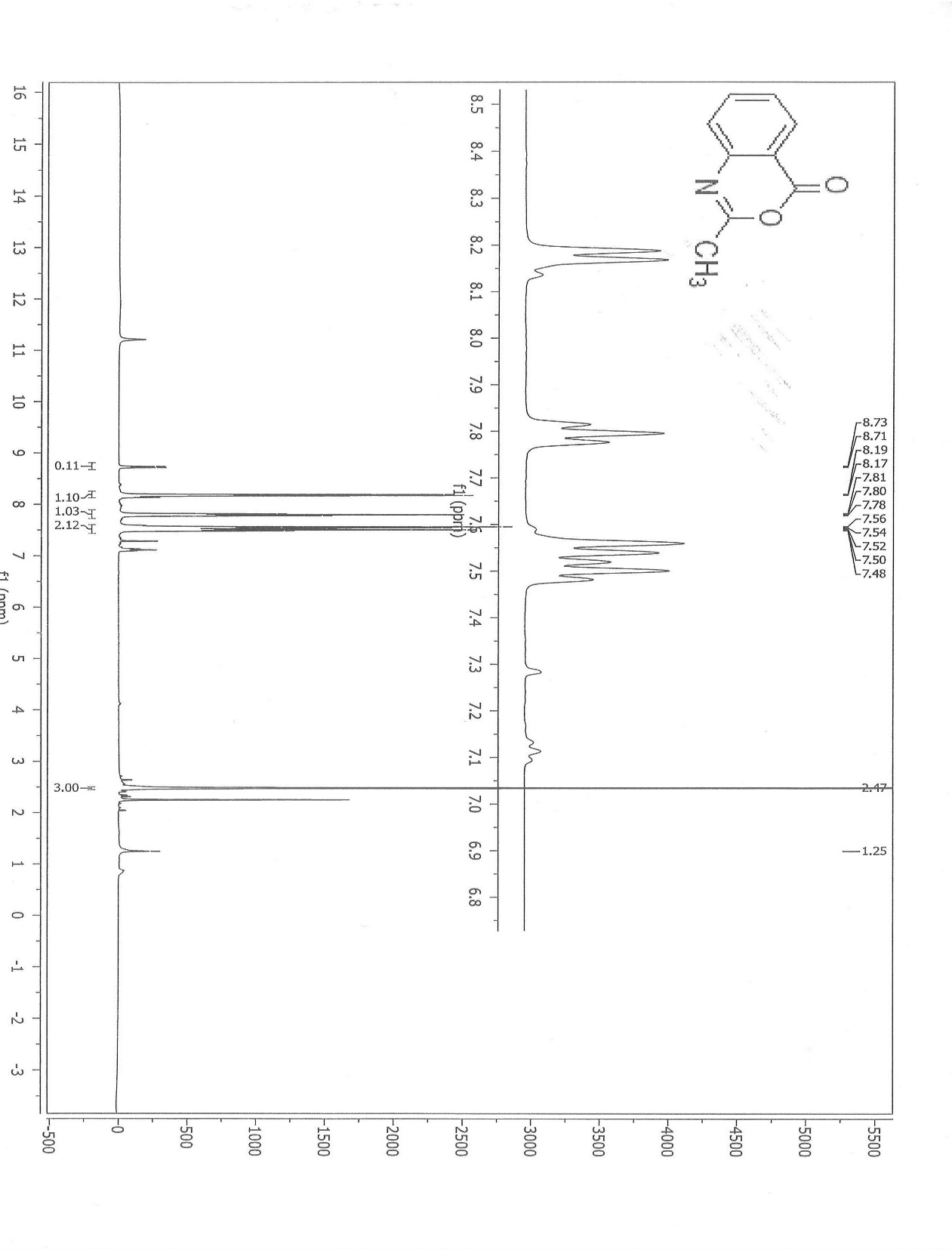


Figure S54. H^1^ spectra of compound (9)


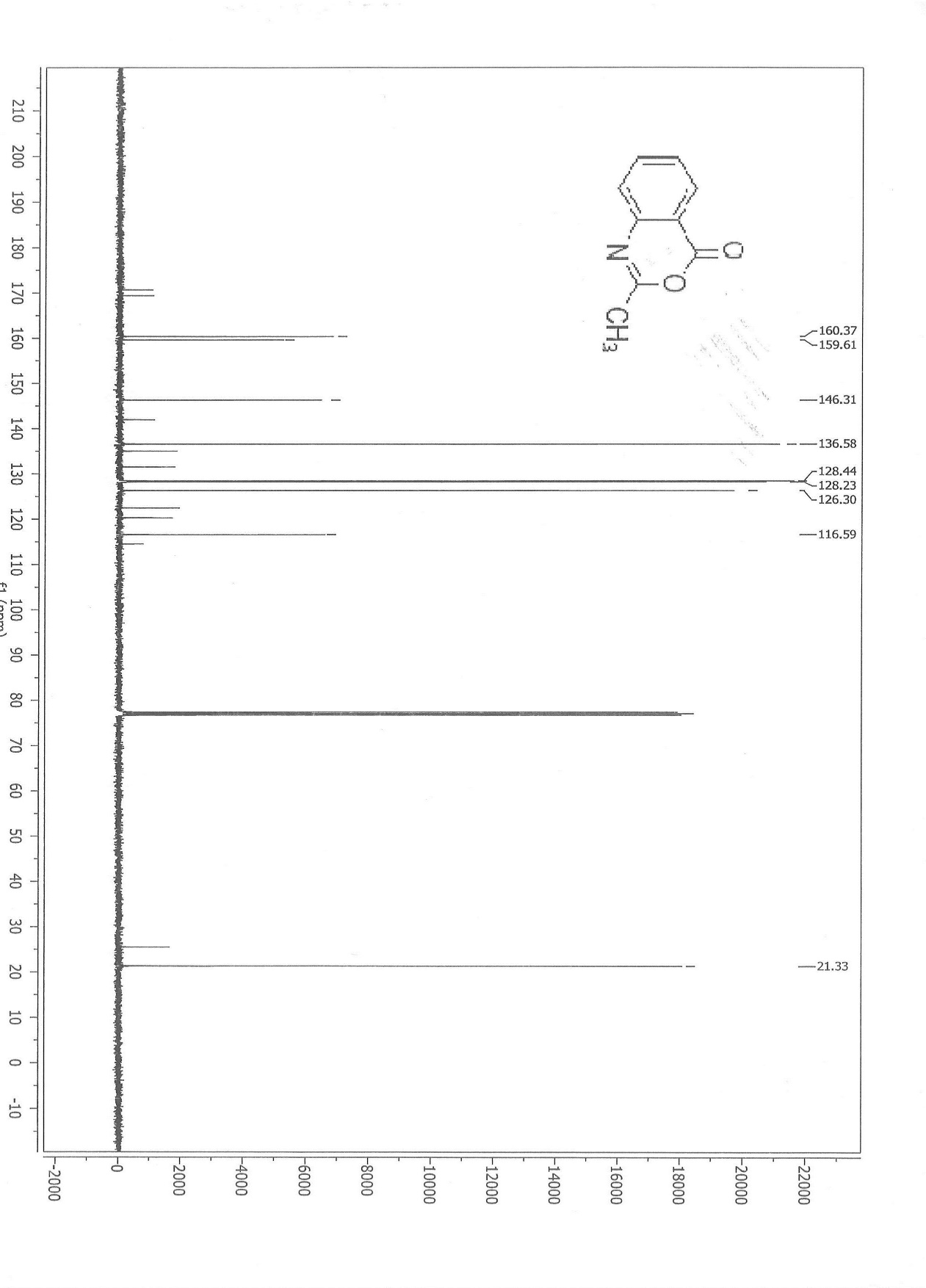


Figure S55. C^13^ spectra of compound (9)


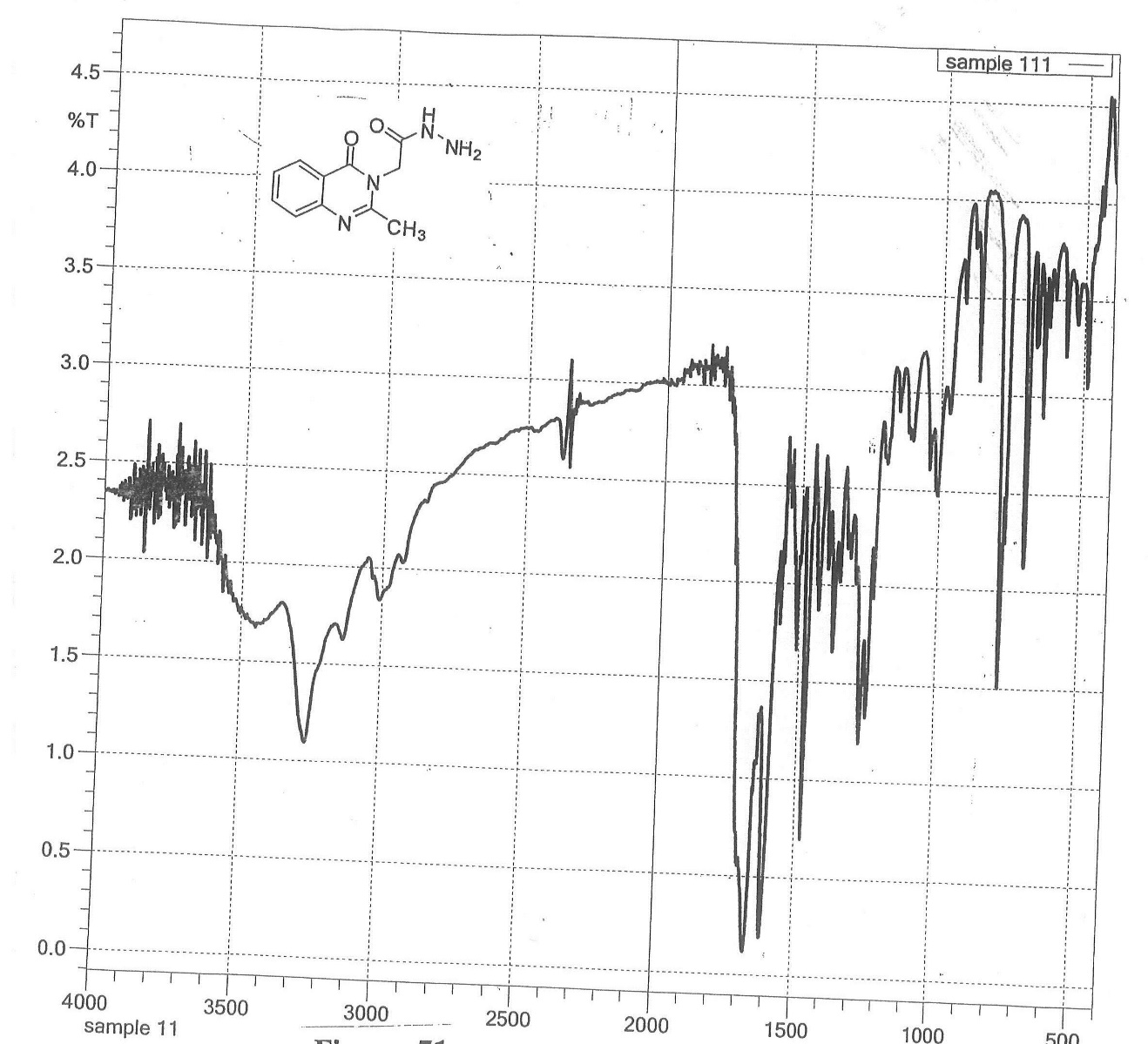


Figure S56. IR spectra of compound (11)


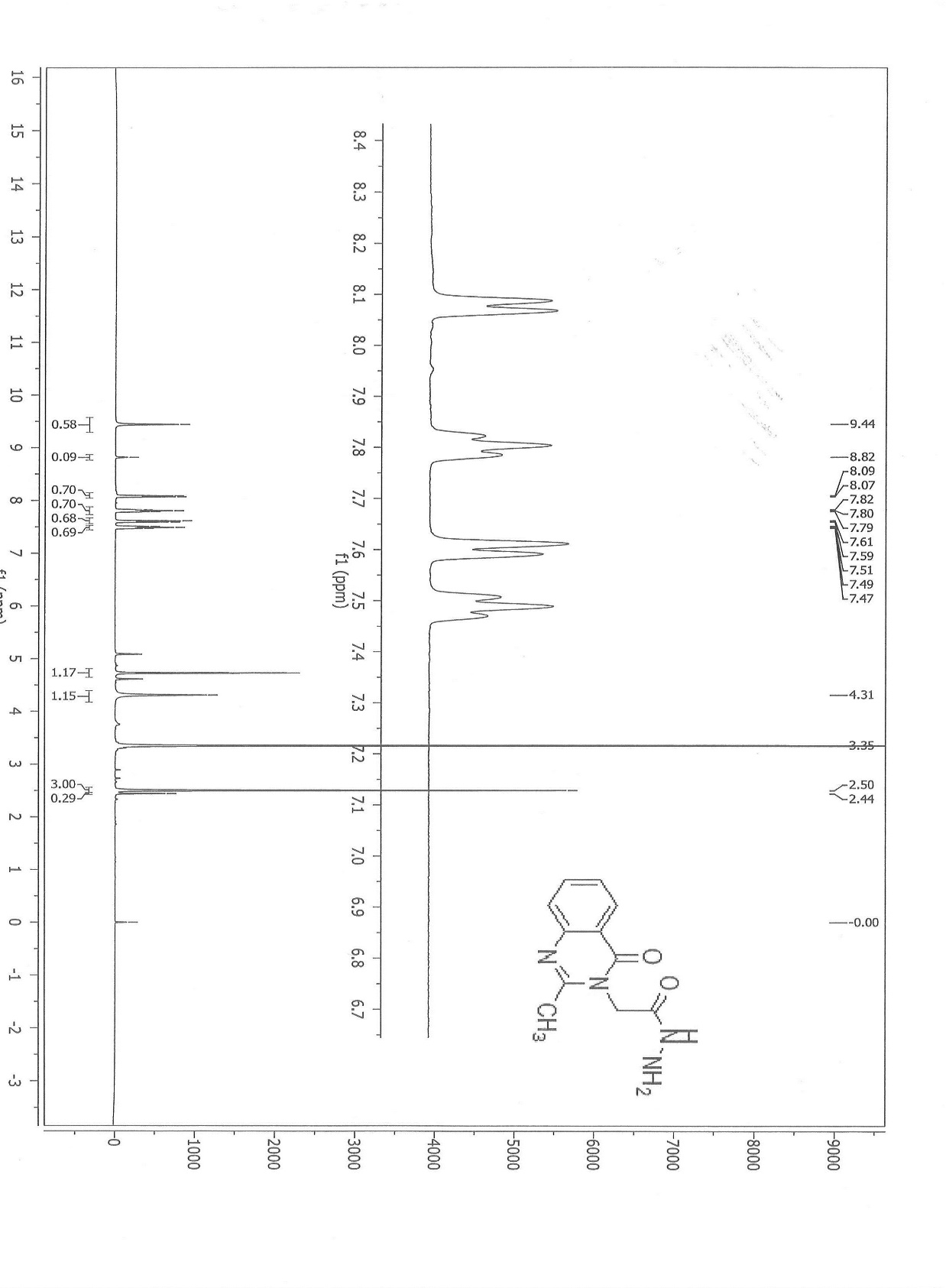


Figure S57. H^1^ spectra of compound (11)


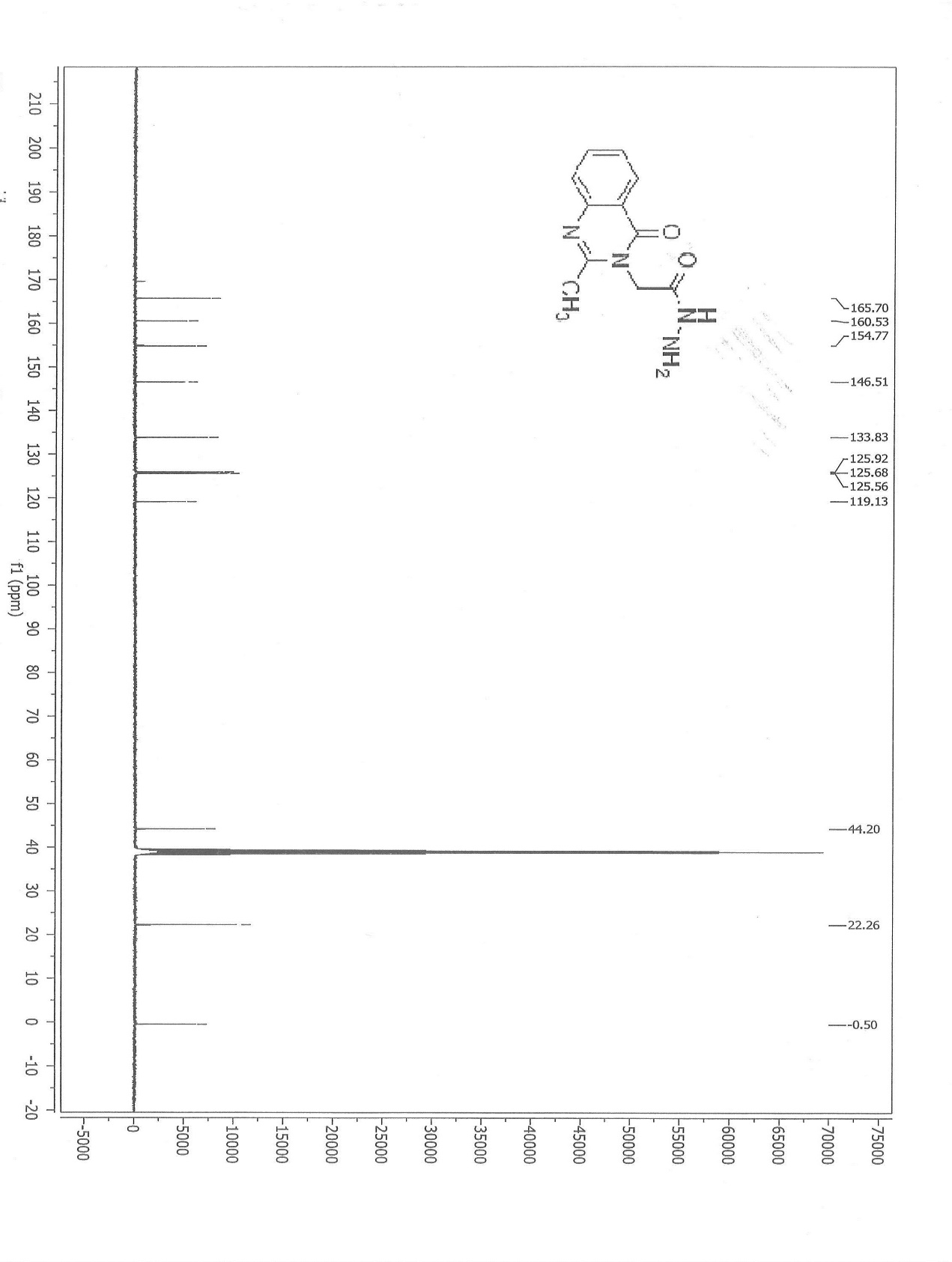


Figure S58. C^13^ spectra of compound (11)


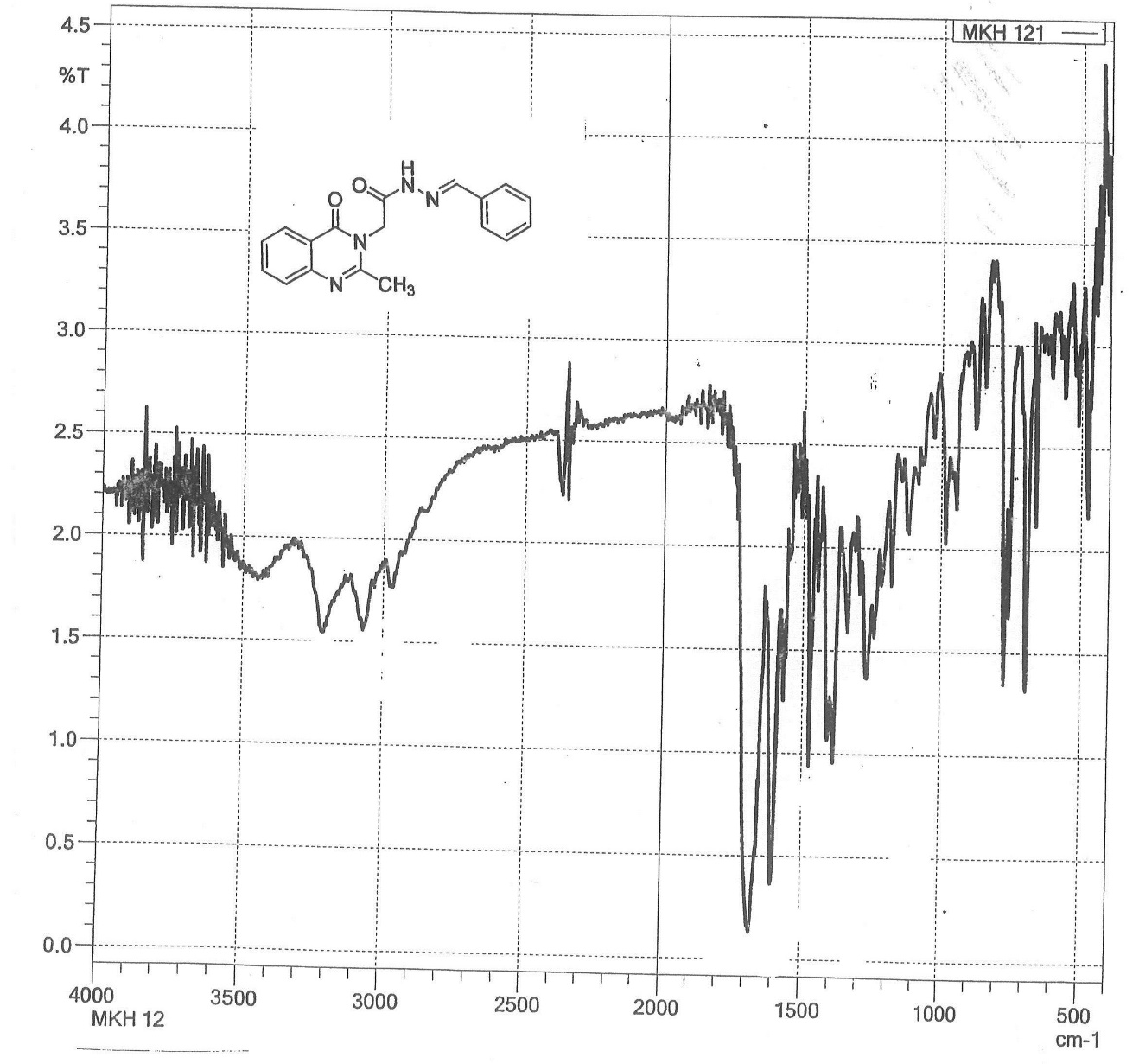


Figure S59. IR spectra of compound (12a)


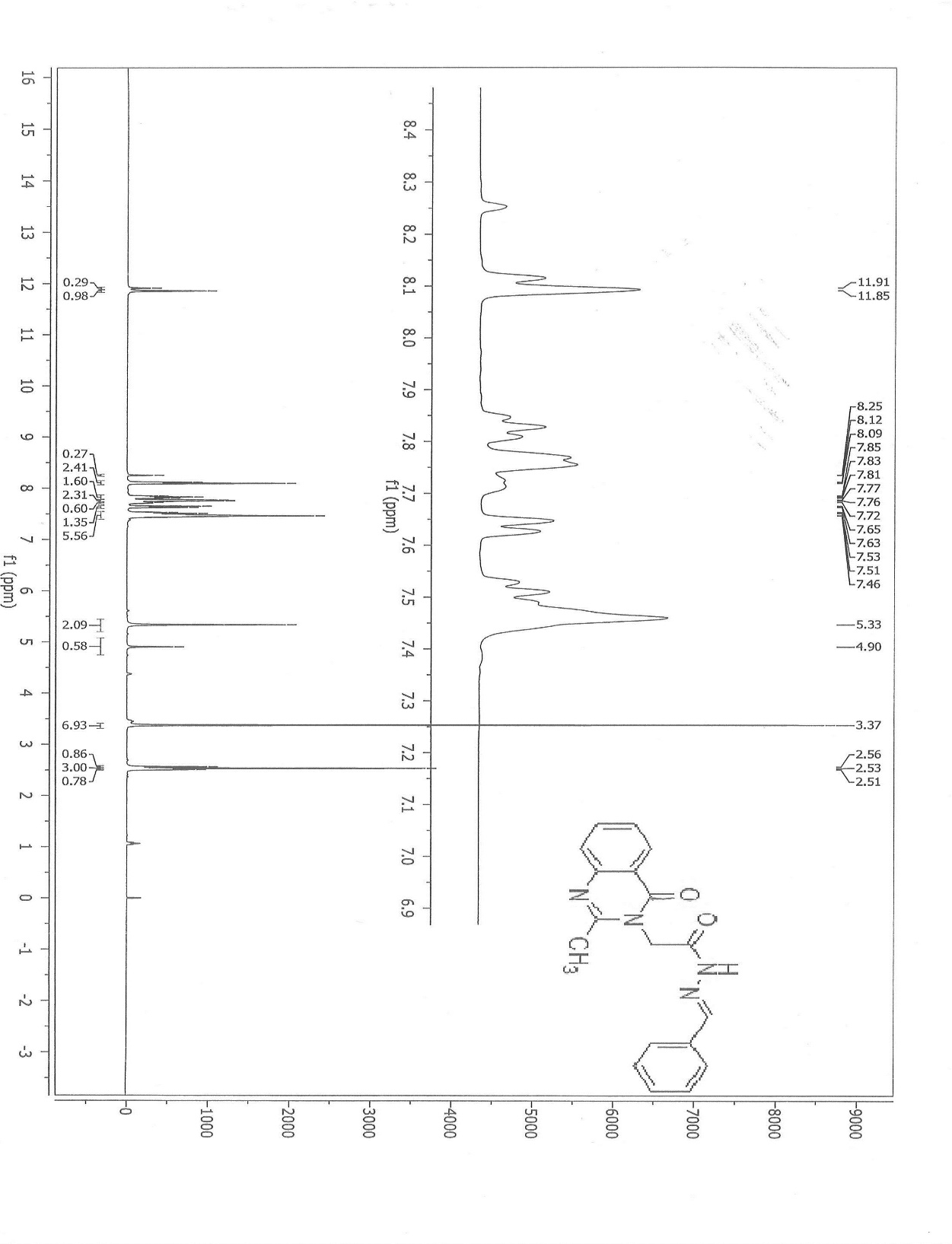


Figure S60. H^1^ spectra of compound (12a)


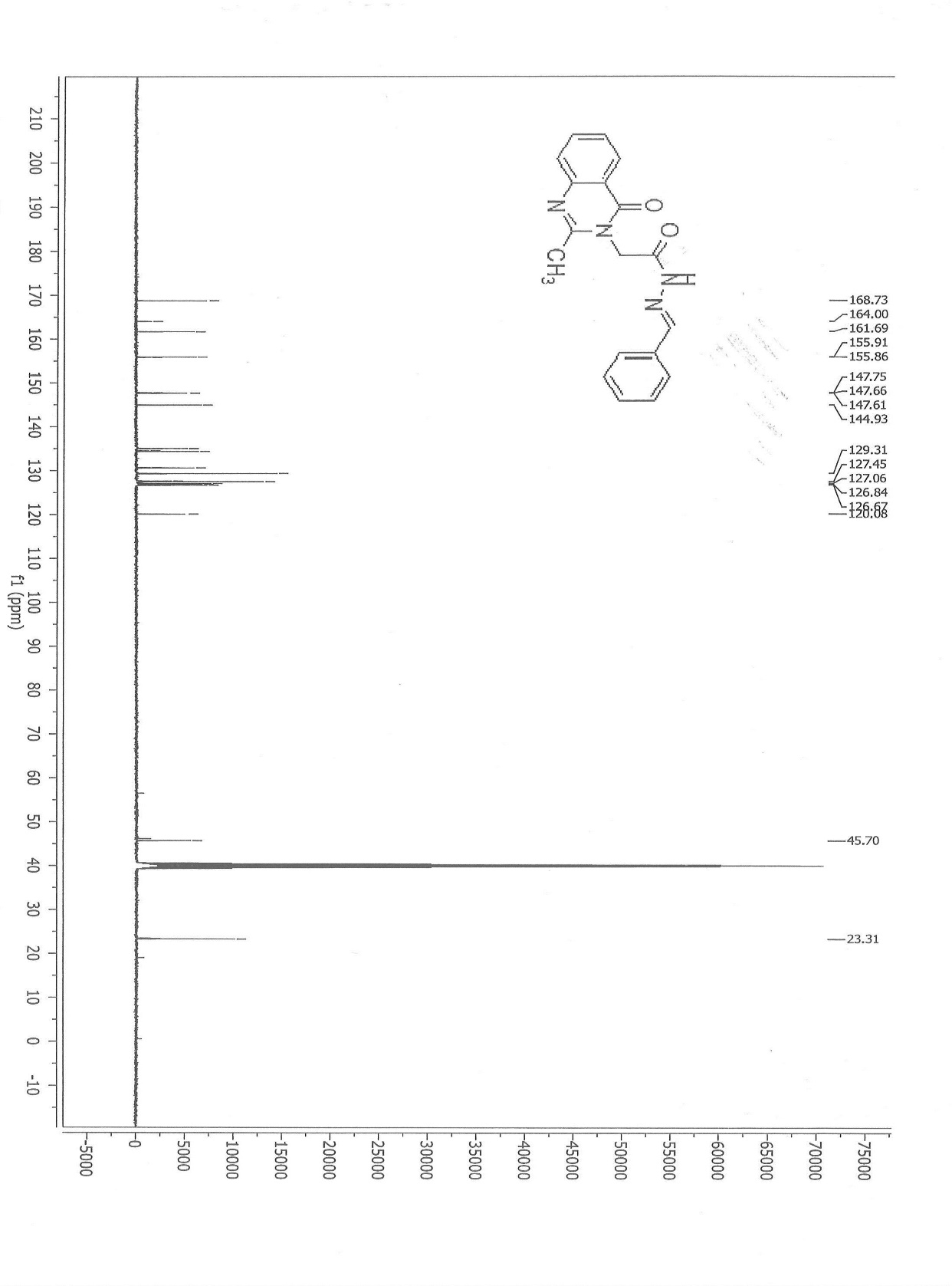


Figure S61. C^13^ spectra of compound (12a)


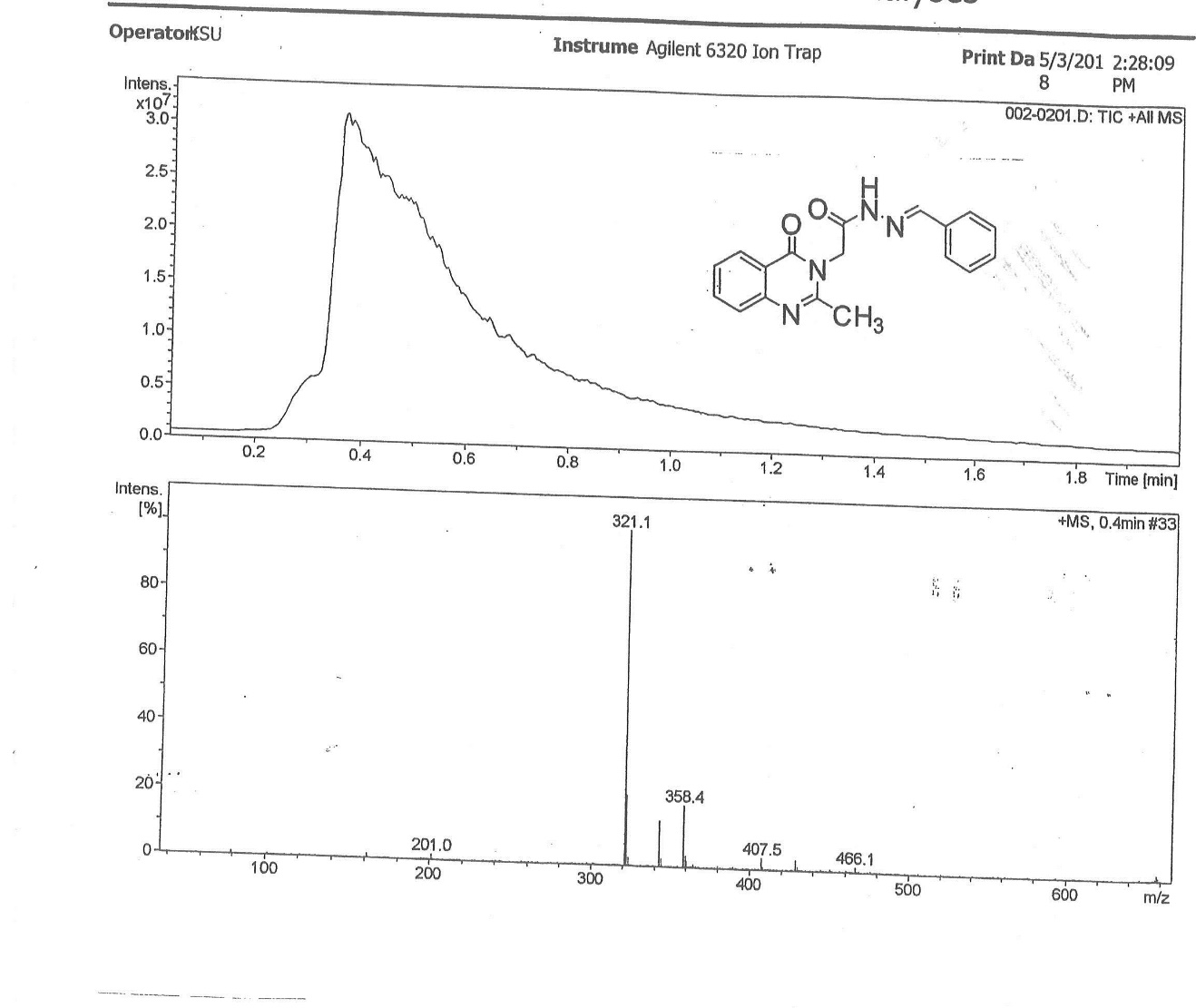


Figure S62. MS-ESI spectra of compound (12a)


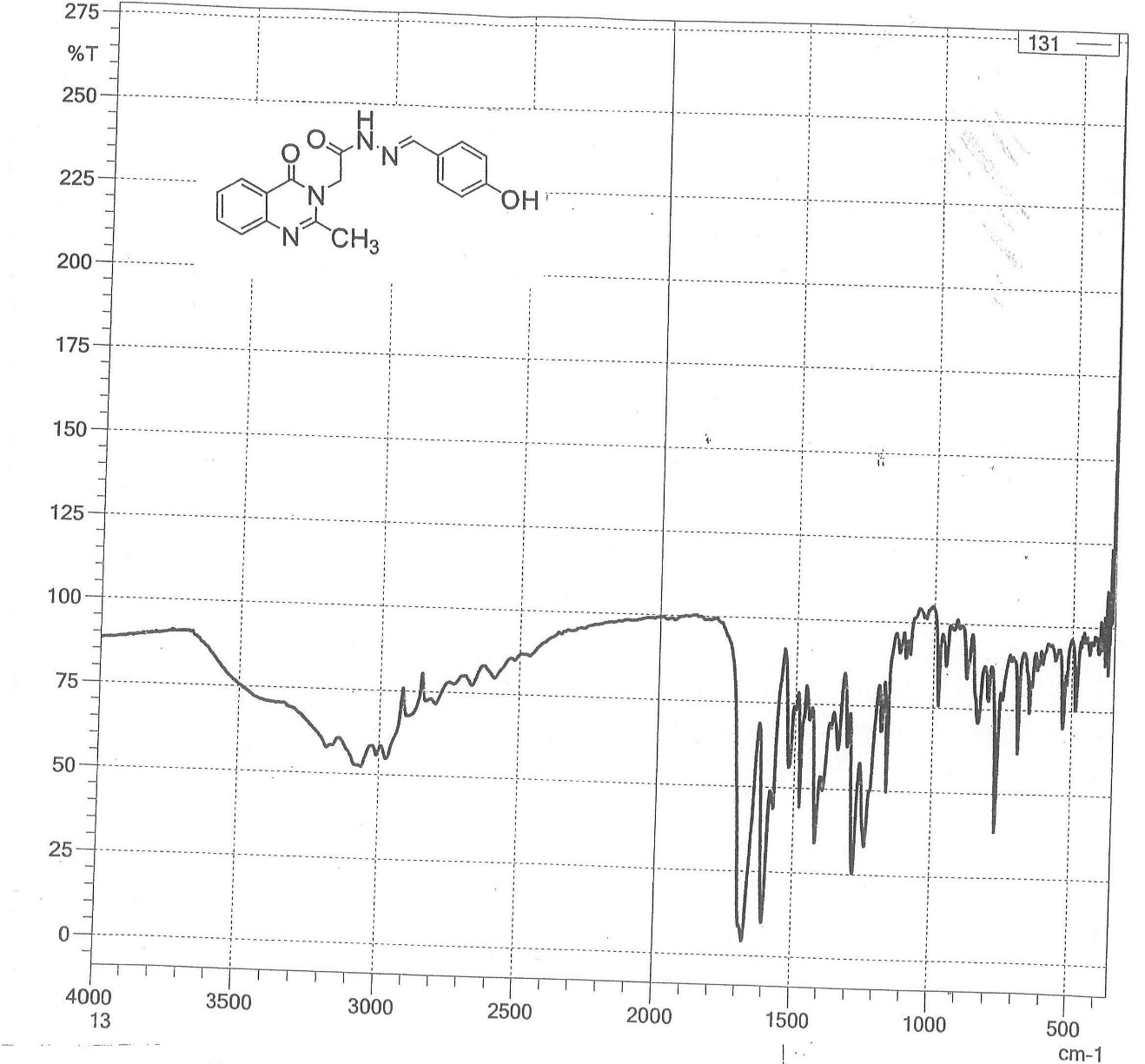


Figure S63. IR spectra of compound (12b)


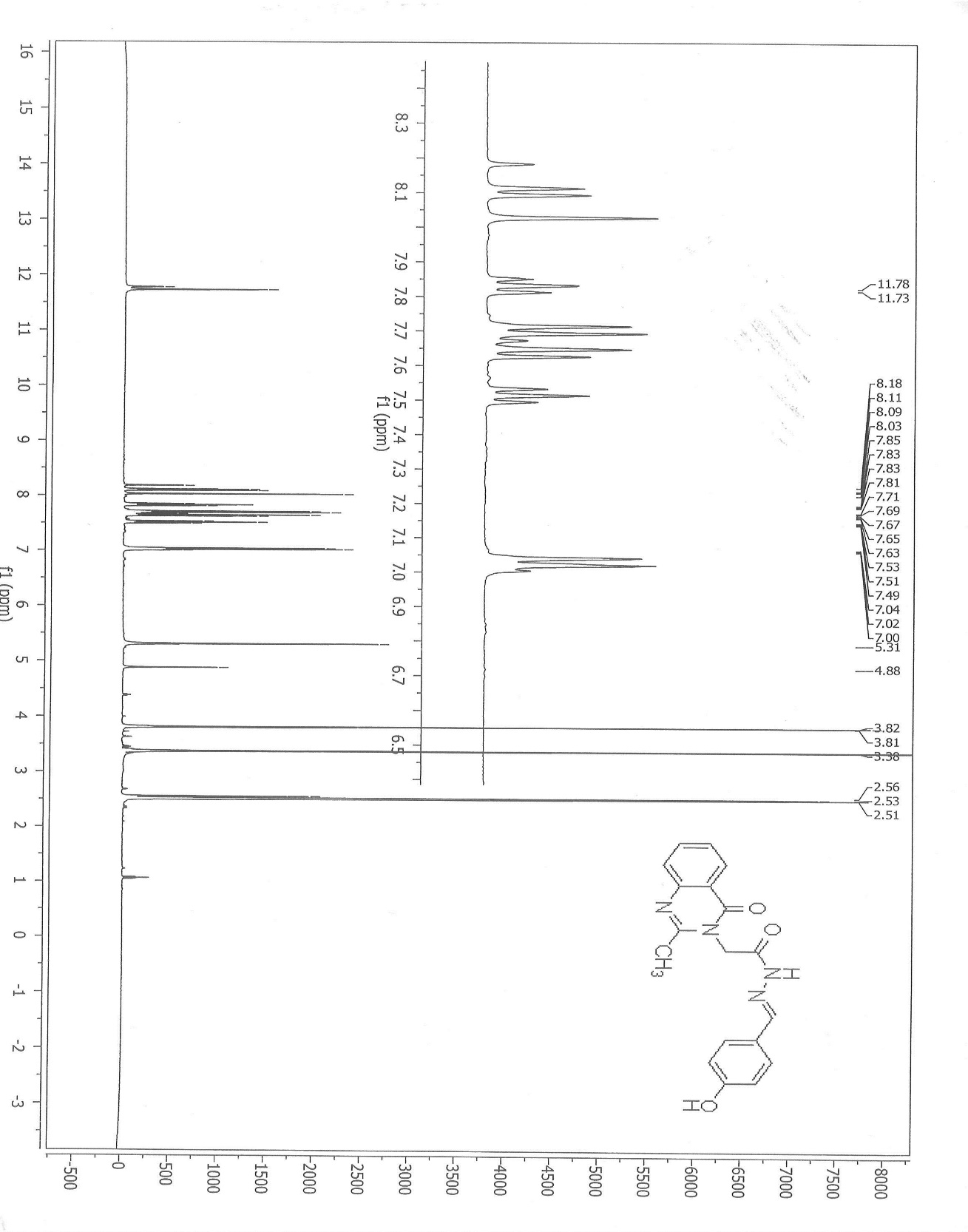


Figure S64. H^1^ spectra of compound (12b)


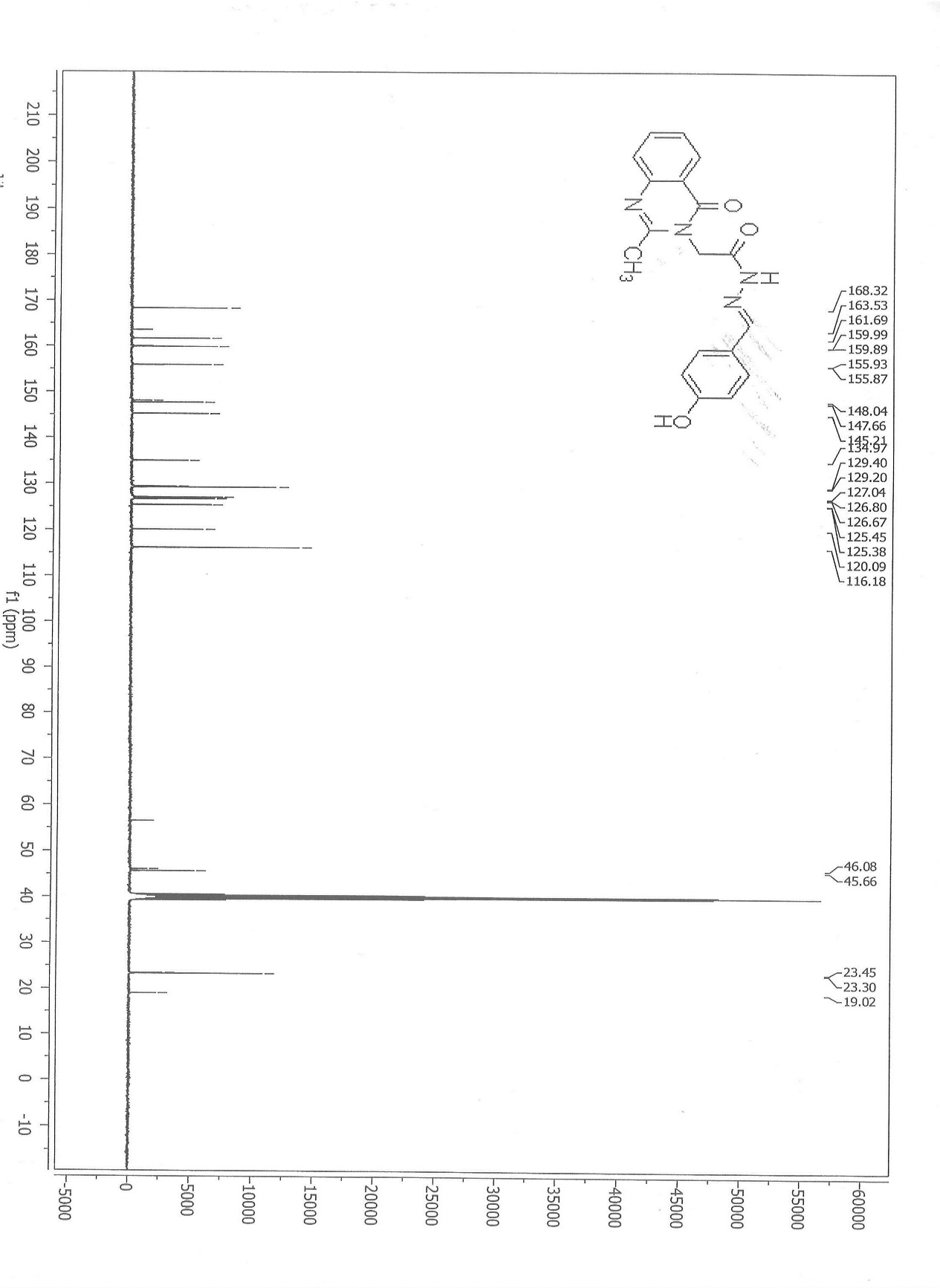


Figure S65. C^13^ spectra of compound (12b)


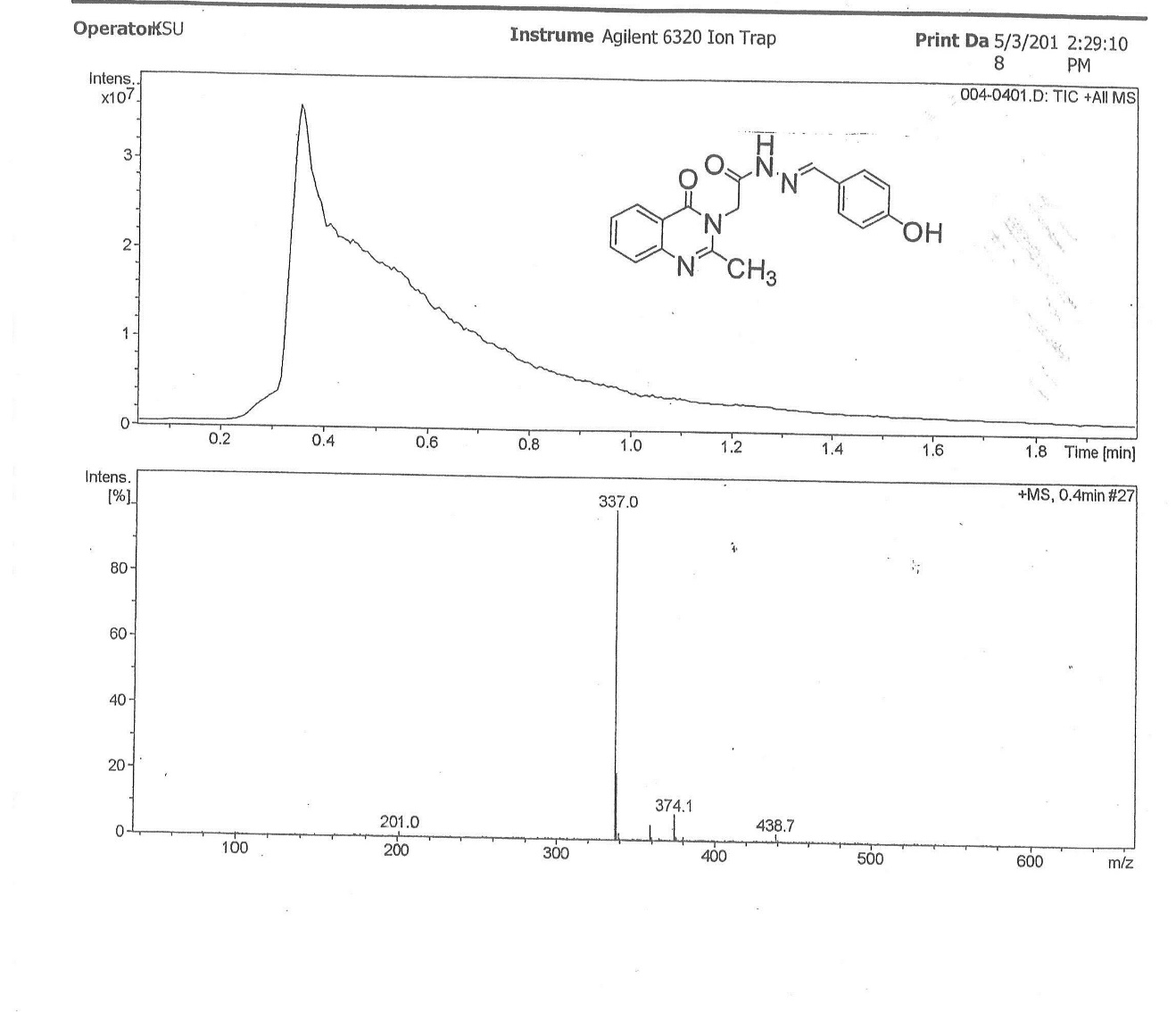


Figure S66. MS-ESI spectra of compound (12b)


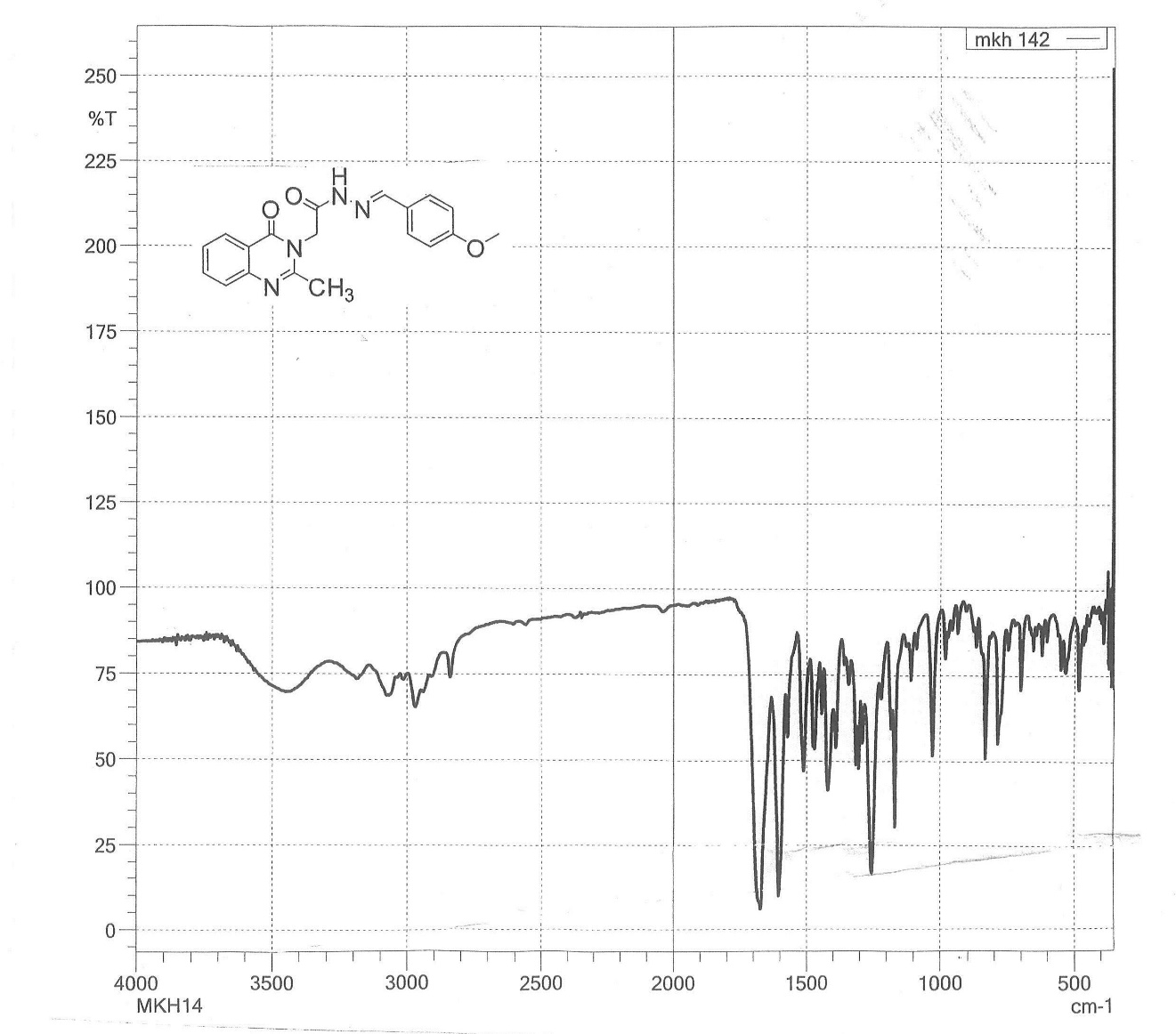


Figure S67. IR spectra of compound (12c)


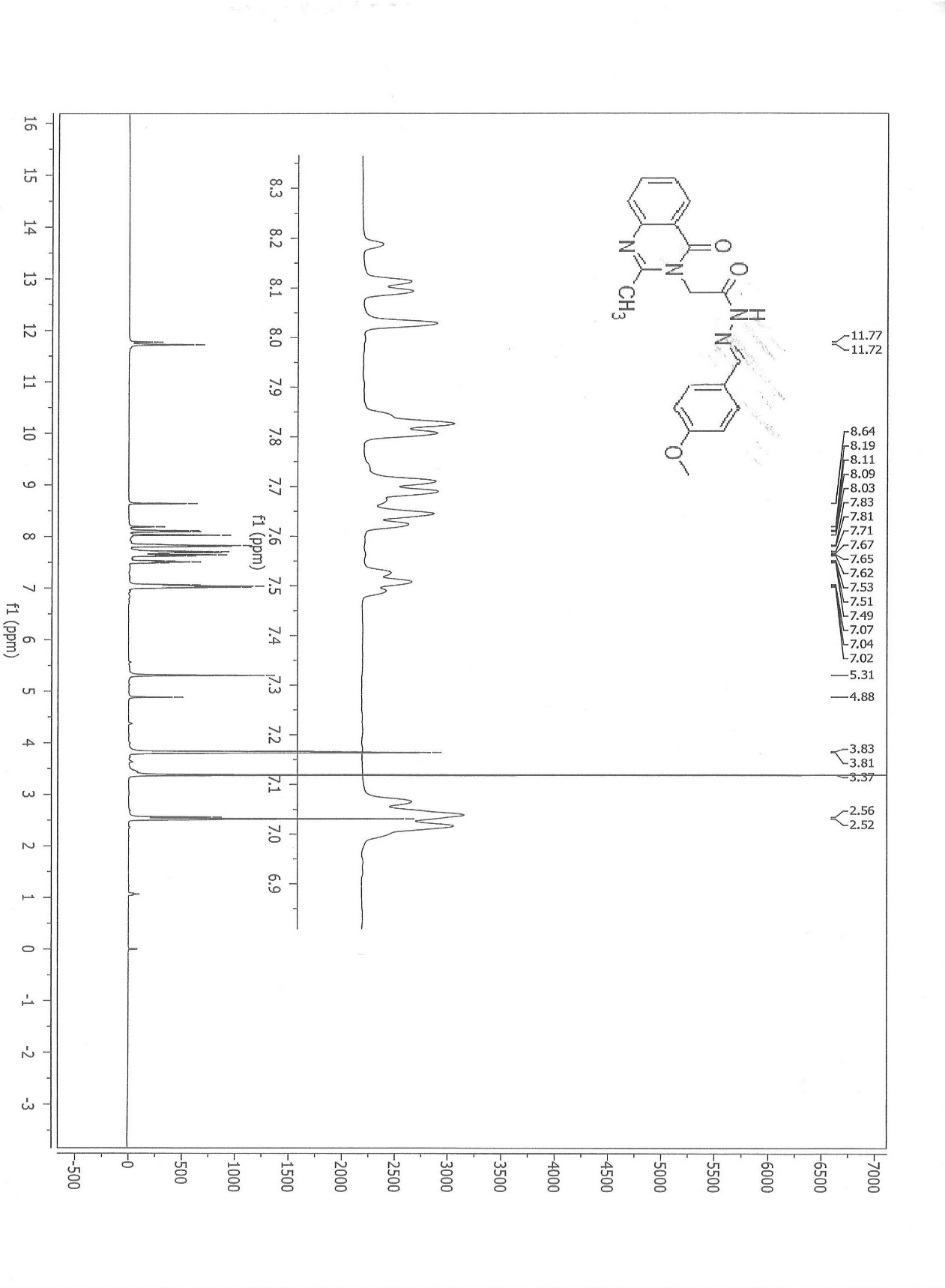


Figure S68. H^1^ spectra of compound (12c)


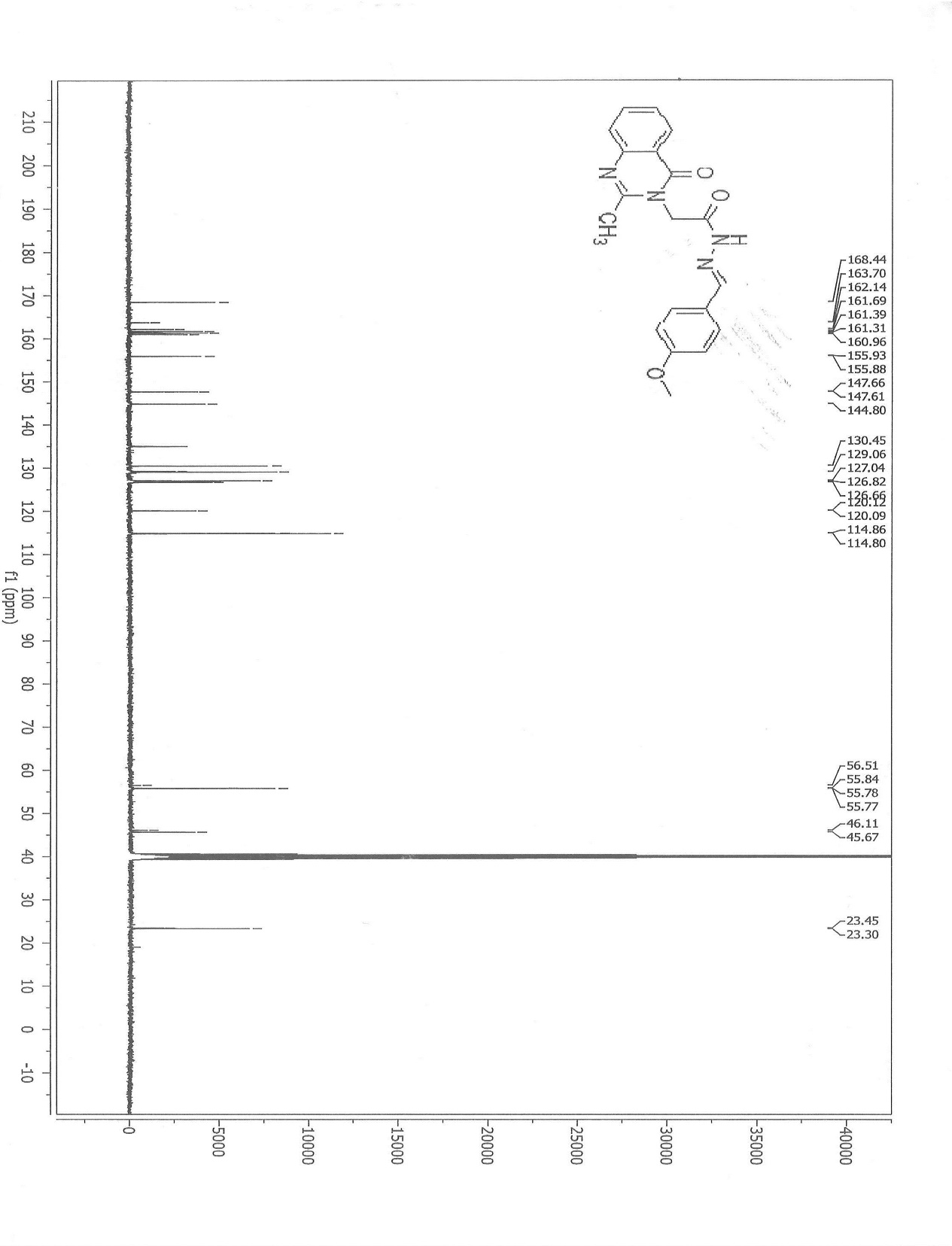


Figure S69. C^13^ spectra of compound (12c)


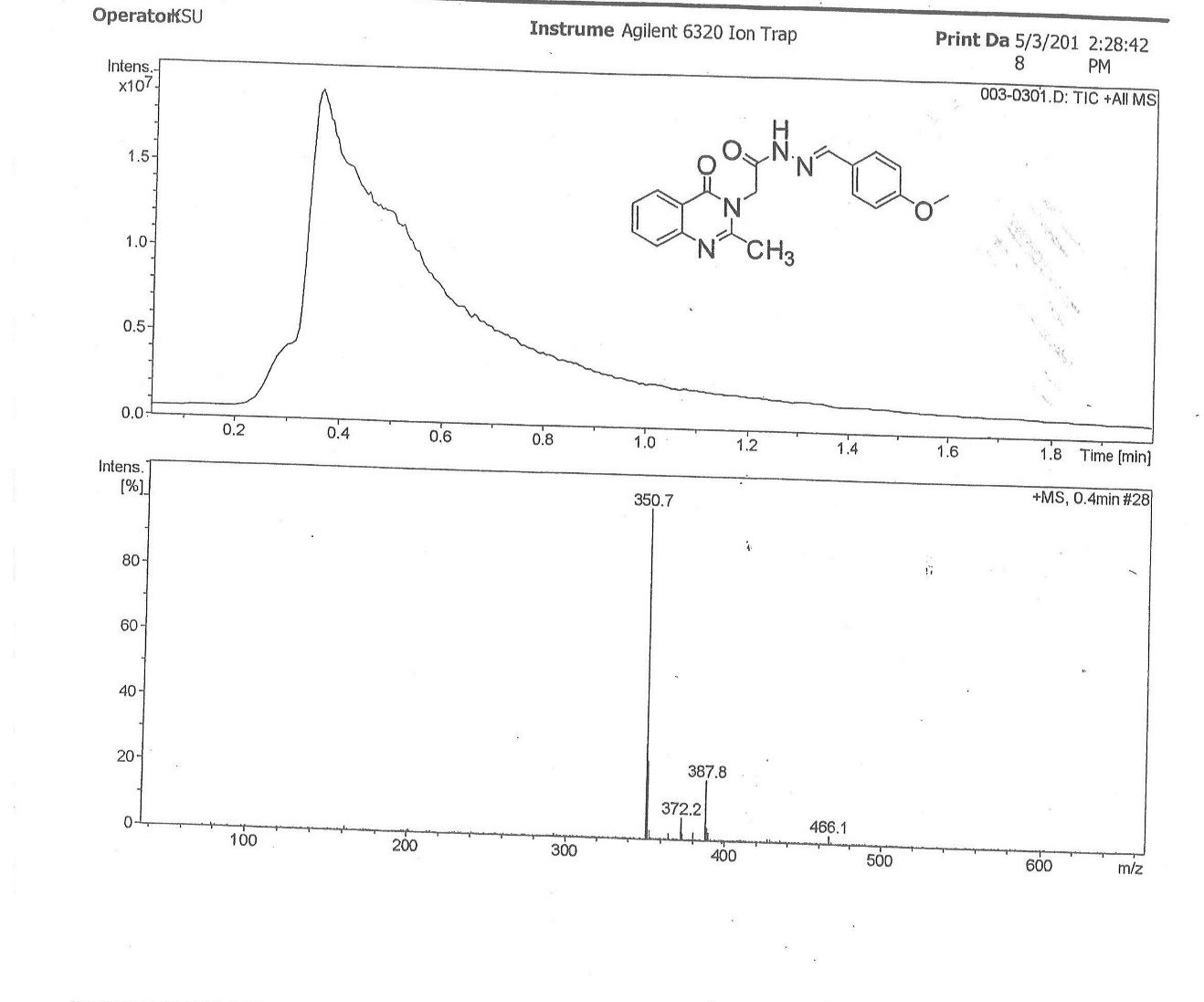


Figure S70. MS-ESI spectra of compound (12c)


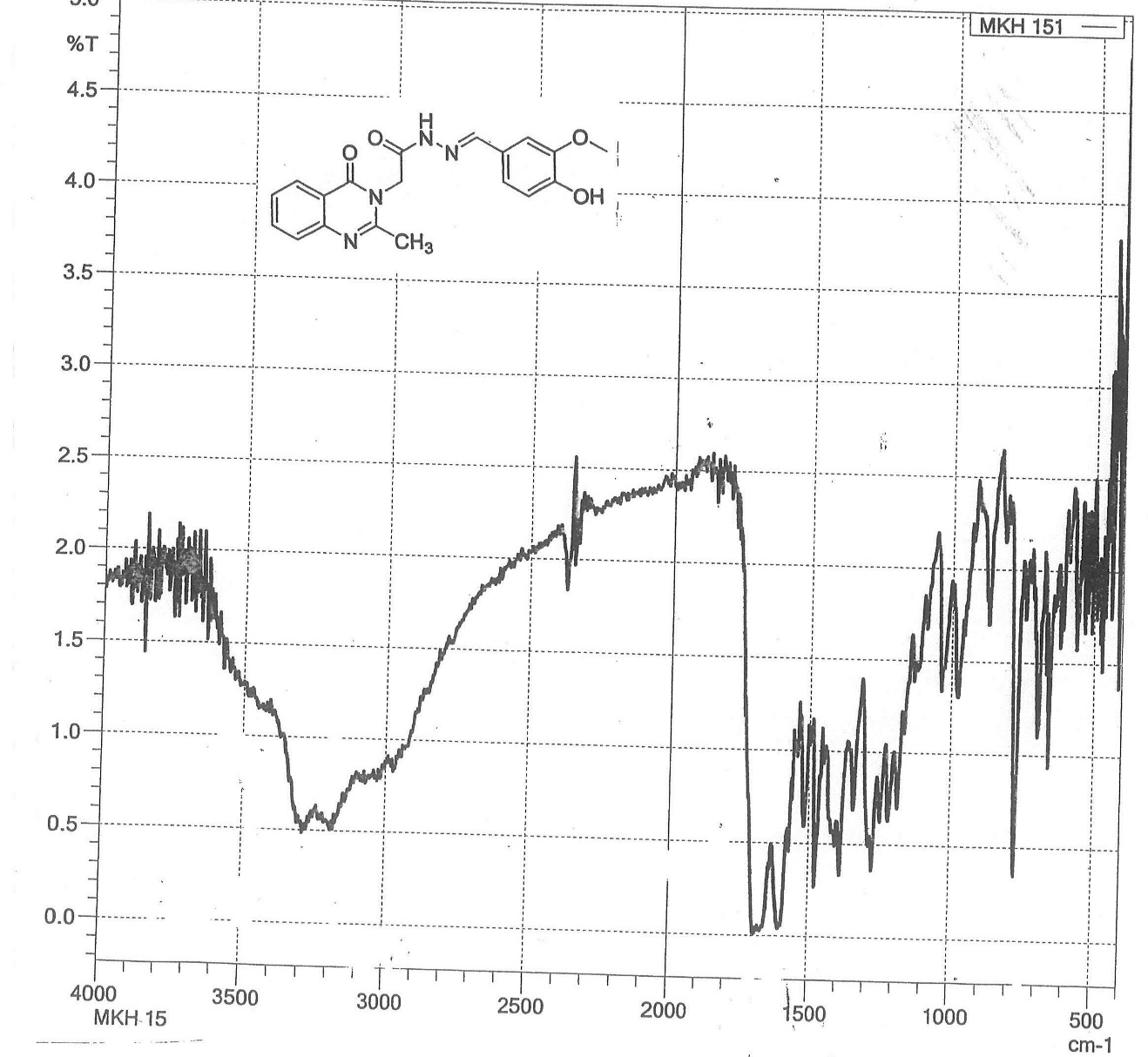


Figure S71. IR spectra of compound (12d)


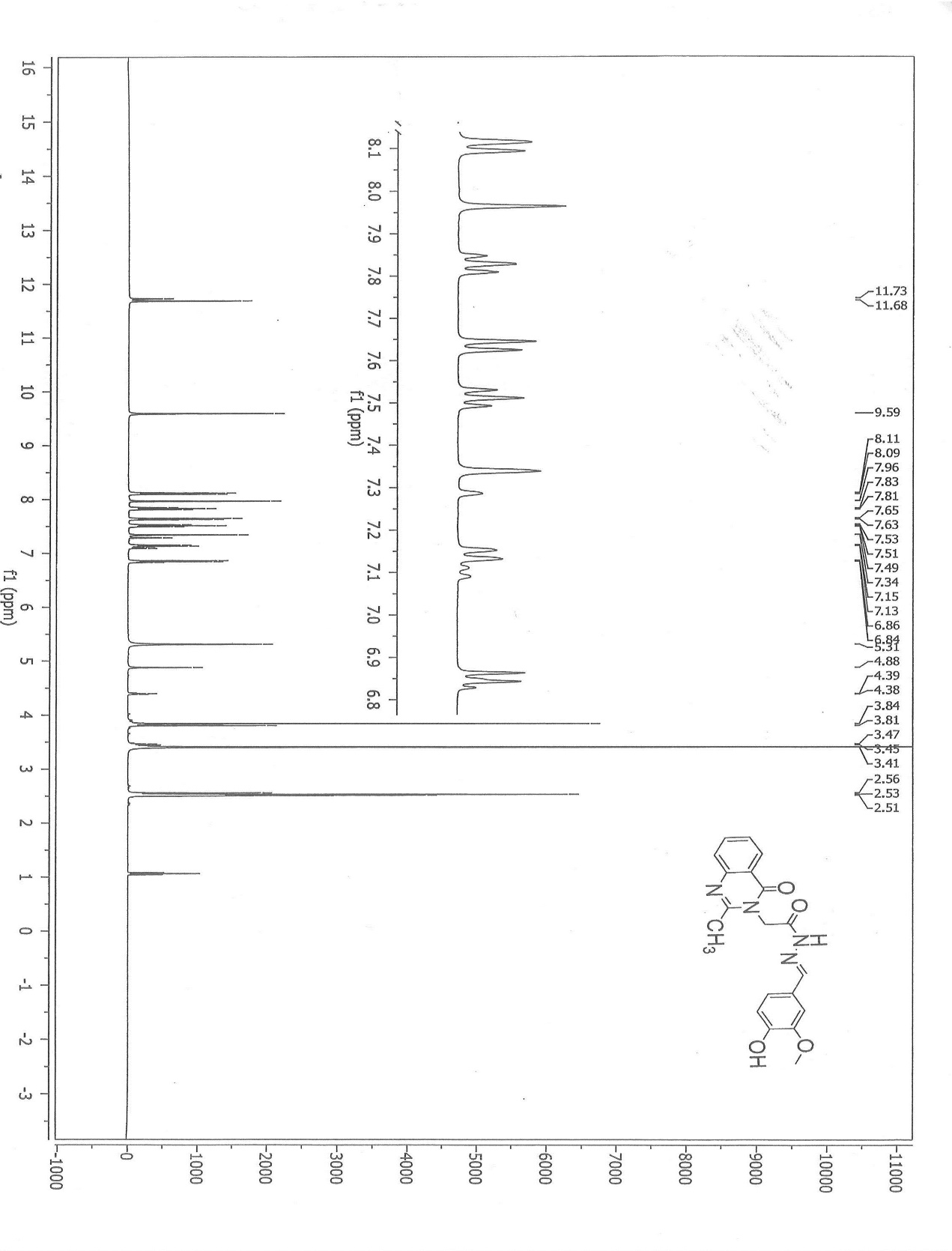


Figure S72. H^1^ spectra of compound (12d)


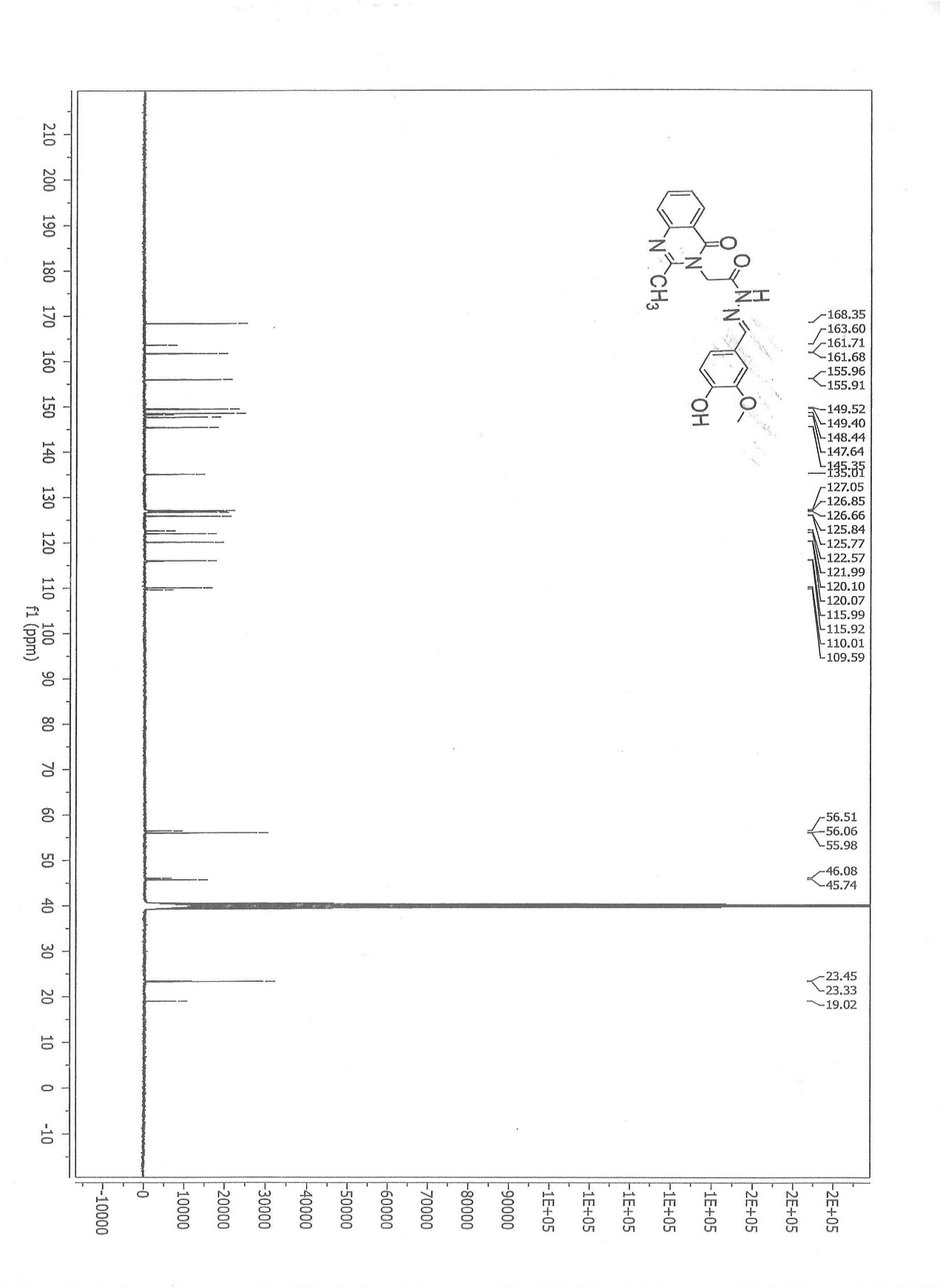


Figure S73. C^13^ spectra of compound (12d)


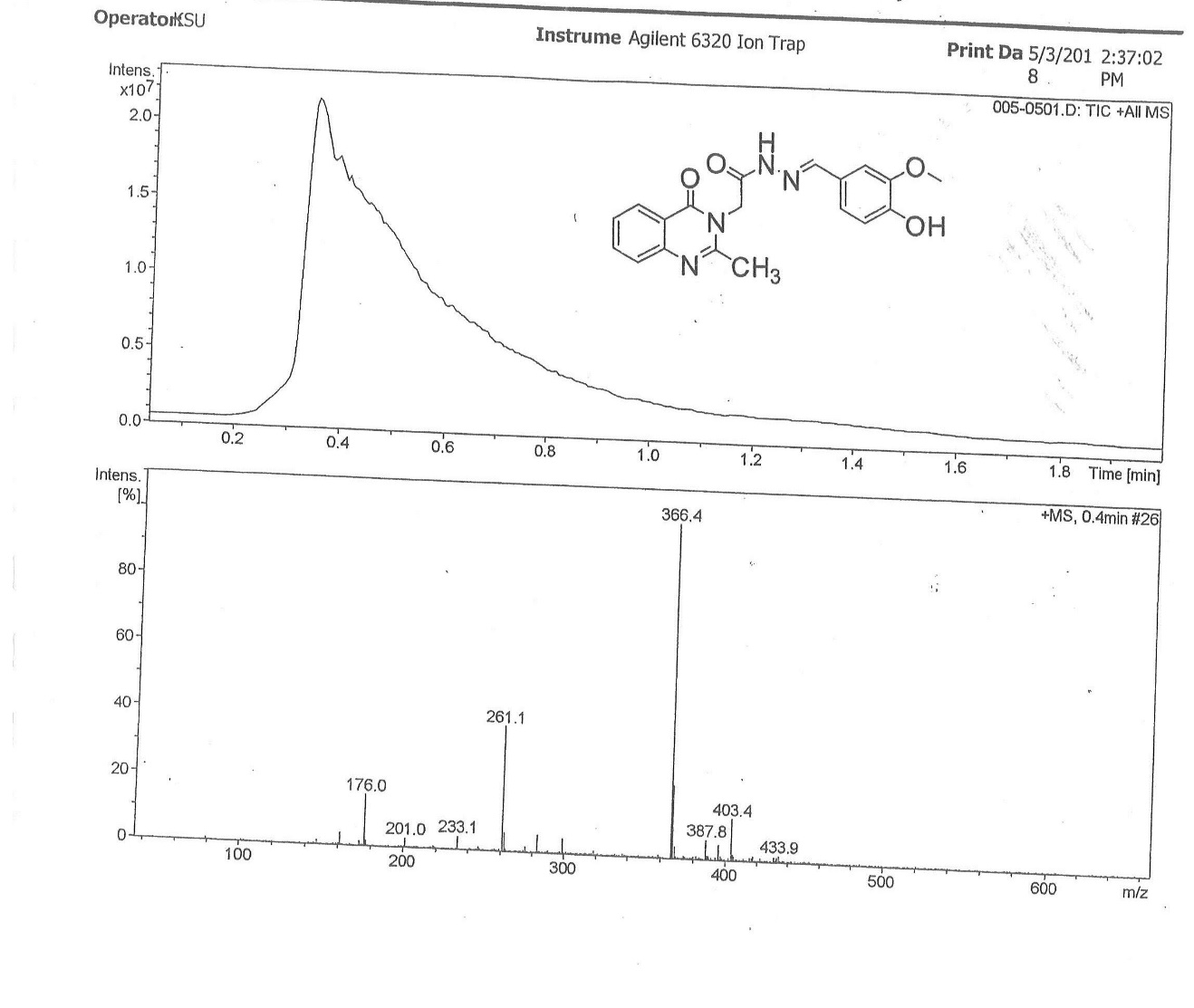


Figure S74. MS-ESI spectra of compound (12d)
